# Toward reliable computer-aided brain tumor diagnosis: a contrast-enhanced deep learning approach with hybrid KNN classification

**DOI:** 10.3389/fnhum.2026.1883543

**Published:** 2026-09-14

**Authors:** Krithika Pandurangan, Doddi Chandana Gowri, Jonnla Naga Surekha, Pratiksha V. Chafle, Kolagotla Venkateswara Reddy, Shonak Bansal, Krishna Prakash, Md. Shabiul Islam, Mohammad Tariqul Islam

**Affiliations:** 1 Department of MBA, SRM Institute of Science and Technology, Chennai, Tamil Nadu, India; 2 Department of CSE, SRM University, Amaravathi, India; 3 Department of Electronics and Communication Engineering, Dr RVR NRI Institute of Technology (Deemed to be University), Eluru, India; 4 Department of CSE, G H Raisoni University Amravati, Maharashtra, India; 5 Department of Mechanical Engineering, Lakireddy Balireddy College of Engineering, Vijayawada, India; 6 Department of Electronics and Communication Engineering, Chandigarh University, Gharuan, Punjab, India; 7 Department of Information and Communication Technology, Marwadi University, Rajkot, Gujarat, India; 8 Centre for Advanced Devices and Systems, Centre of Excellence for Robotics and Sensing Technologies, Multimedia University, Cyberjaya, Selangor, Malaysia; 9 Faculty of Artificial Intelligence and Engineering, Multimedia University, Cyberjaya, Selangor, Malaysia; 10 Department of Electrical, Electronic and Systems Engineering, Faculty of Engineering and Built Environment, UKM Bangi, Bangi, Selangor, Malaysia

**Keywords:** brain tumors, convolutional neural networks, magnetic resonance imaging, multi-scale contrast enhancement, supervised learning approaches

## Abstract

Timely and accurate diagnosis of brain tumors using magnetic resonance imaging (MRI) is a major concern in neuro-oncology because of the potential effects of delayed and inconsistent radiological diagnoses on the management of such diseases. Manual analysis of MRIs is time-consuming and highly susceptible to observer variations; thus, there is a need for computer-based and more consistent diagnostic processes. In this study, an automatic diagnostic process based on deep learning was proposed using multiscale contrast enhancement along with lightweight convolutional features and classifiers for diagnosing four classes of brain tumors. The MRI images were analyzed using a Multi-scale Intensity Contrast Amplification (MICA) algorithm that enhances the relevant structural information of the tumors across fine, intermediate, and coarse spatial scales. The refined images were then fed into an in-house convolutional neural network (CNN) backbone architecture that uses depth-wise separable convolution, residual skip connection, and squeeze-and-excitation (SE) attention mechanism to extract compact and discriminative feature representations. To select the optimal decision layer for this representation, three supervised learning methods, namely K-Nearest Neighbors (KNN), Support Vector Machine (SVM), and Random Forest (RF), were trained on this set of features and compared with the softmax head of the CNN. The experiments were performed on a publicly available, three-source combined MRI dataset of 7,023 images across glioma, meningioma, pituitary tumor, and normal categories using both hold-out and 5-fold cross-validation setups. In all the evaluated configurations, the hybrid model of CNN and KNN provided the best hold-out accuracy (96.58%), whereas the hybrid model of CNN and SVM offered the best macro-average precision (98.81%) and area under the ROC curve (99.56%). Both hybrids were statistically superior (*p* < 0.001) to the softmax head of the CNN in the paired statistical tests. Class Activation visualization based on gradient analysis revealed that the network concentrated on clinically sensible regions of the tumors rather than other irrelevant background regions. With a compact parameter footprint (0.20 MB in half precision) and an inference throughput of approximately 23.7 images per second on the CPU and 220.1 images per second on an NVIDIA A100 GPU at a batch size of 16, the proposed framework offers an accurate, interpretable, and computationally frugal solution that is suitable for computer-aided brain tumor screening in resource-constrained clinical settings.

## 1 Introduction

This section establishes the clinical setting in which the automated brain tumor classification operates. It outlines the epidemiology and taxonomy of the principal primary brain tumors, reviews the imaging modalities on which their diagnosis depends and the reasons magnetic resonance imaging is preferred among them, and identifies the methodological shortcomings of existing automated approaches that motivate the framework developed in the remainder of this paper.

Brain tumors represent a wide range of medical conditions that occur when brain cells or cells in nearby tissues grow uncontrollably. According to cancer data, approximately 1.6% of people worldwide with cancer develop tumors that hinder essential neurological functions. The Global Cancer Observatory GLOBOCAN reports that approximately 308,000 new cases of cancer are detected worldwide every year (Sung et al., 2021).

The classification of brain tumors plays a vital role in understanding their behavior and treatment procedures during the diagnosis process. Brain tumors are typically categorized as primary or secondary. Primary brain tumors originate within the brain, whereas secondary brain tumors are metastatic lesions that originate from cancers such as lung, breast carcinoma, or melanoma. This study mainly focused on primary brain tumors. Gliomas, meningiomas, and pituitary adenomas are frequently occurring among the primary brain tumors. Gliomas (Hersh et al., 2023) are the most common cancerous (malignant) brain tumors and include astrocytomas, oligodendrogliomas, and ependymomas. Meningiomas (Soni et al., 2025) arise from the meninges and are typically non-cancerous (benign) but can become symptomatic due to their size or location. Pituitary adenomas (Daly and Beckers, 2020) develop in the pituitary gland as mostly benign tumors that cause endocrine disorders when they disrupt hormonal processes.

The use of medical imaging technology makes brain tumor diagnosis more accurate and effective. Magnetic resonance imaging (MRI) is the gold standard for brain imaging because it offers an in-depth description of soft tissues and their boundaries. The human brain functions through two sophisticated methods: functional MRI (fMRI) and diffusion-weighted imaging (DWI), which show the function and structure of the brain. Computed tomography (CT) scans offer faster results than MRIs; however, they fail to provide the same amount of information regarding tumors, hemorrhages, and edema as MRIs. Positron Emission Tomography (PET) imaging technology is used in combination with other imaging technologies as a source of metabolic activity information, which is used to determine whether a tumor is malignant. The medical field faces a significant challenge in detecting brain tumors using magnetic resonance imaging (MRI). Different approaches have been used to classify tumors precisely for that purpose through the process of medical image processing and advanced deep learning (DL) techniques, according to some studies (Khan et al., 2025; Kumar et al., 2025). Tumor classification is based on the first-, second-, and higher-order statistical measures of texture, which are important features of texture analysis. The classifier requires additional features, including transform-based, histogram-based local, and shape-based descriptors (Sowjanya et al., 2023). Machine learning (ML) algorithms use features with the help of support vector machines (SVM), K-nearest neighbors (KNN), and ensemble methods such as boosting and bagging. All these methods help discriminate gliomas, meningiomas, and pituitary tumors in MRI images. All these methods require handcrafted features to function. The introduction of advanced deep learning through convolutional neural networks (CNNs) helps overcome the issues in the traditional method. These methods were used for feature extraction and classification. In the last few years, the main research area of scientists has moved towards DL-based approaches.

The development of deep learning systems has made significant advancements; however, essential operational aspects of medical applications remain unaddressed. Existing common CNN models instantiate standard convolution processes, which restrict their ability to detect meningioma tissue through their fixed field-of-view feature extraction methods and broader audiovisual elements. The system encounters it’s most significant challenge when different classes share similar visual traits, and their boundaries remain difficult to detect. Most MRI scans experience quality loss because of magnetic field variations and coil sensitivity artifacts, which create dimmer tumor borders. Only a small number of researchers have used an MRI-based contrast normalization technique that specifically addresses MRI noise types before feature extraction, which causes networks to learn about artifacts during their training process. Hybrid CNN classifier systems demonstrate potential for success; however, researchers have not explored the direct application of compact CNN models with non-parametric classifiers to handle multiple brain tumor classes.

The practical consequences of these shortcomings are most readily appreciated through a concrete diagnostic example. On contrast-enhanced T1-weighted slices, a convexity meningioma presents as a broad-based, dural-attached mass whose signal intensity may exceed that of the adjacent cortex by only a narrow margin, whereas a low-grade glioma presents as an intra-axial lesion whose margins fade gradually into the surrounding white matter. When such a slice is additionally affected by the smooth, low-frequency intensity gradient that arises from coil sensitivity or B1-field inhomogeneity, the already narrow contrast at the lesion boundary is compressed further, and the boundary on which the distinction between the two entities rests becomes visually unreliable. In contrast, a pituitary adenoma occupies a stereotyped sellar location and is therefore separable on positional grounds alone, which is why it is seldom the limiting class in reported results. Figure 1 depicts this situation directly, showing a representative slice before and after the multi-scale enhancement proposed here, whereas Figure 2 shows the regions on which the trained network bases its decision for each of the four categories. The pipeline as a whole, from enhancement through feature extraction to the decision layer, is shown in Figure 3.

**Figure 1:**
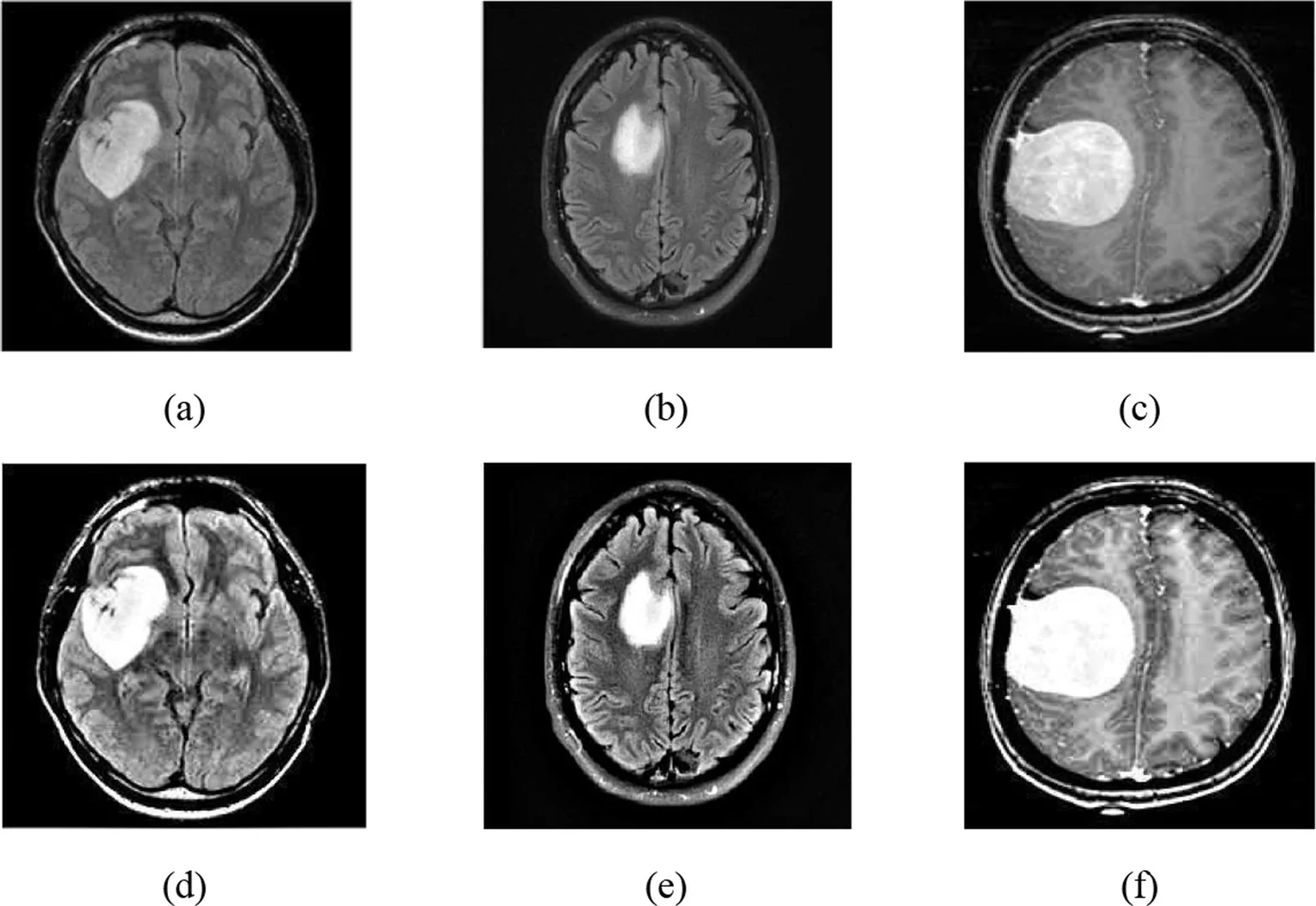
Visual results of the MICA-based contrast enhancement technique: **(a–c)** Original brain MRI images; **(d–f)** enhanced brain MRI images.

**Figure 2:**
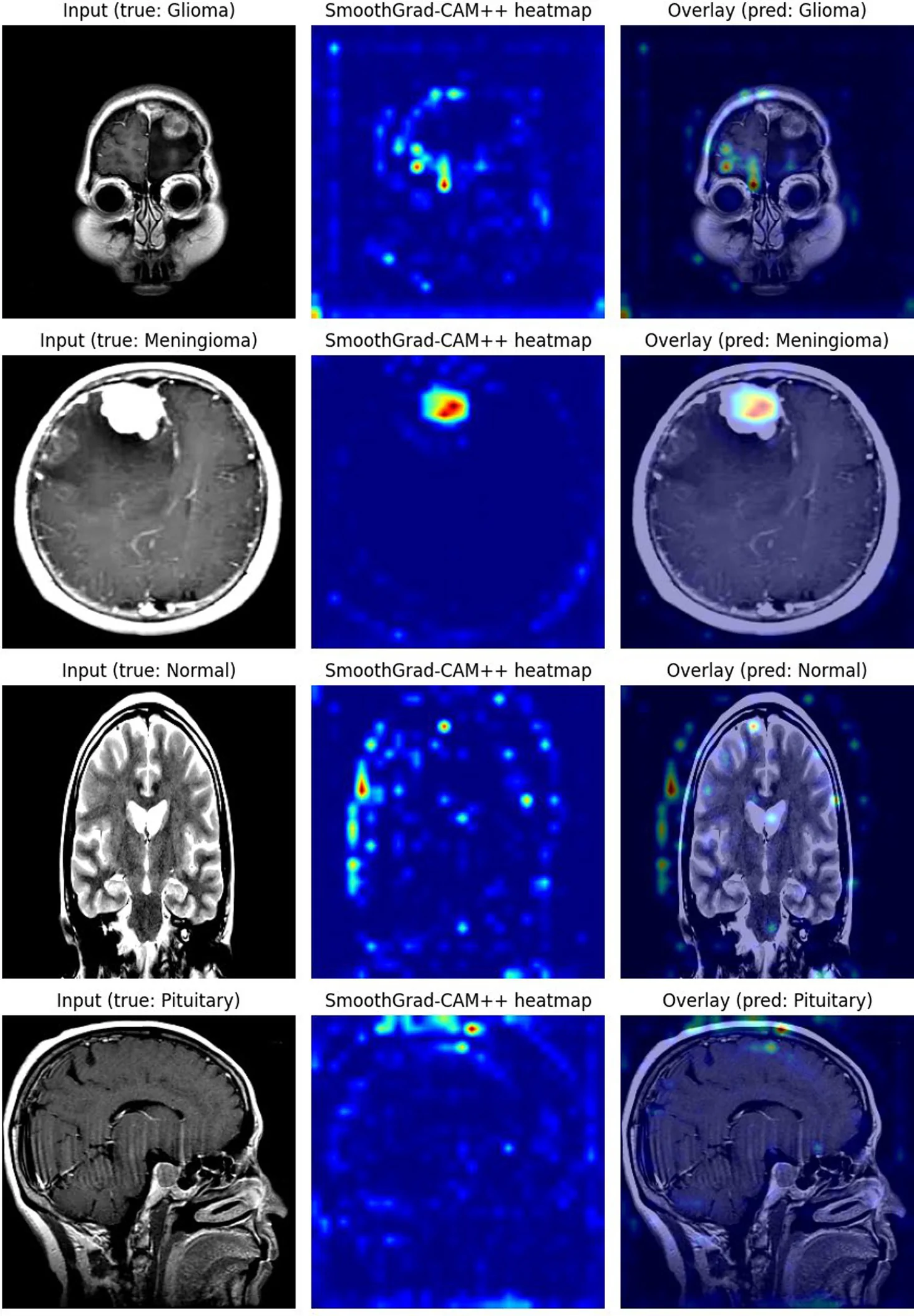
SmoothGrad-CAM++ visualizations for representative glioma, meningioma, normal, and pituitary cases.

**Figure 3:**
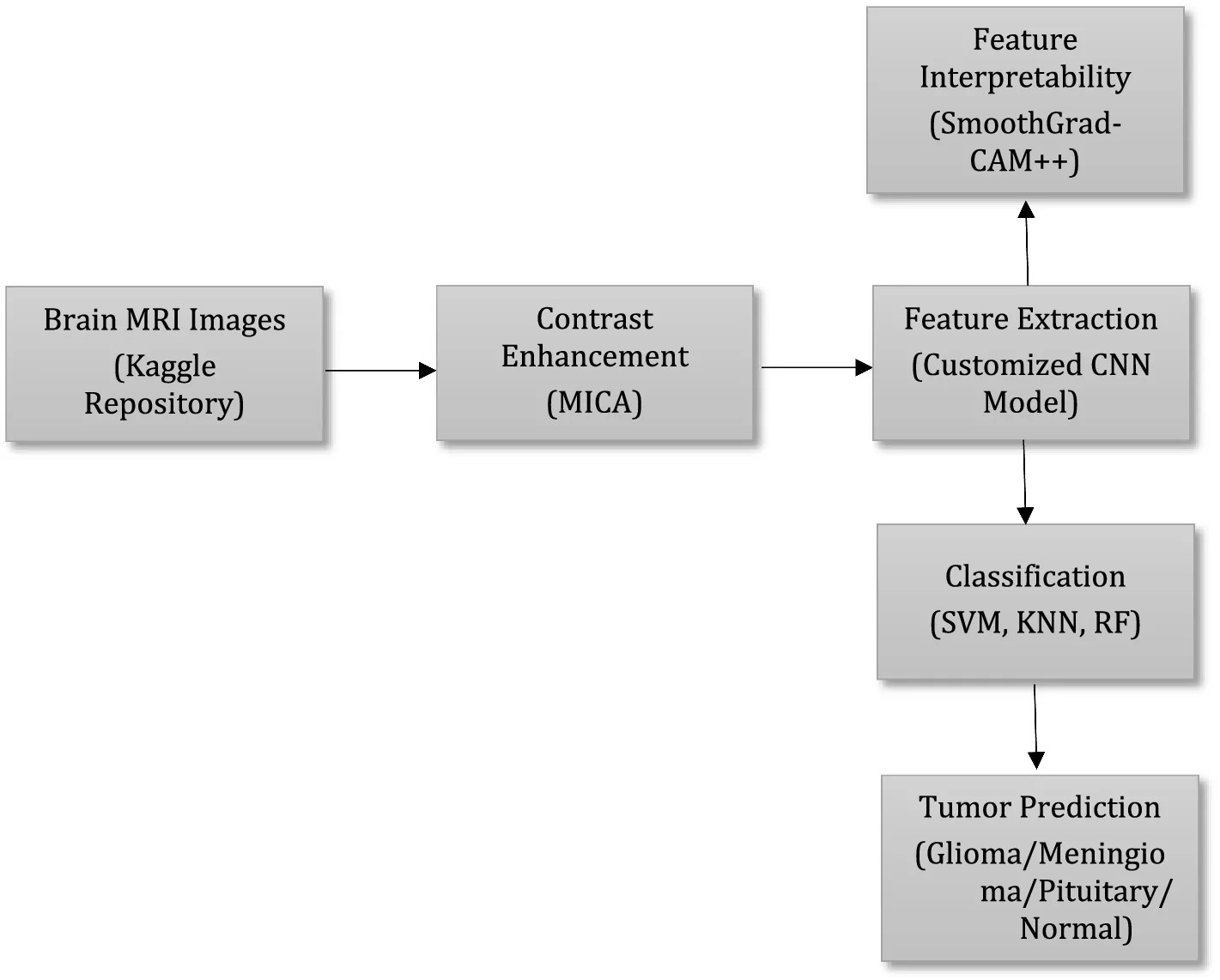
Workflow of the proposed model.

Three specific deficiencies follow from the foregoing and together constitute the research gap that motivates this study. First, contrast normalization is ordinarily applied at a single spatial scale and is seldom validated quantitatively; therefore, the low-frequency intensity variation described above is not removed in a manner demonstrably faithful to the underlying anatomy. Second, the architectural mechanisms that would compensate for this at the level of the representation, namely receptive-field expansion, channel recalibration, and stable gradient propagation, are ordinarily adopted collectively based on the literature rather than justified individually for the task at hand, so that the contribution of each remains unquantified. Third, the decision layer that consumes the learned representation is adopted by convention rather than selection, leaving open the question of which decision rule is best matched to the convolutional embedding of brain-tumor MRI. Together, these three deficiencies create the requirement for a system that combines adaptive multiscale contrast improvement with deep feature extraction under channel-wise recalibration and a decision layer that is selected rather than assumed to be optimal. This requirement was met in the present study. These gaps are stated formally in Section 2.4, once the relevant literature has been surveyed, and the contributions that answer them are enumerated in Section 2.5.

## 2 Literature review

Brain tumor detection through MRI has established a research field with various methods that enhance the accuracy and efficiency of detection procedures. This section examines several advanced frameworks introduced in recent years, focusing on the everyday challenges encountered by these systems and how our approach effectively addresses them. For better comprehensibility and organization, we subdivided the literature review into distinct categories as follows. All these methods are summarized in Table 1, along with their advantages and disadvantages.

**Table 1:** Summary of the state-of-the-art methods.

Author	Models	Pros	Cons
Tang and Teoh (2023)	ResNet-18	Lightweight, fast inference; good feature extraction for small datasets	Limited depth; may miss complex tumor patterns and subtle textures
Woźniak et al. (2023)	CLM and DL	Learns hierarchical MRI features; robust with large datasets	Requires large data; risk of overfitting without regularization
Gürsoy and Kaya (2024)	DL and GNN	Captures relational and structural tumor information	High computational cost; depends on graph quality
Zhou et al. (2024)	FL and EfficientNet-B0	Parameter-efficient; FL protects patient privacy	Federated learning is communication-heavy; reduced performance on non-IID data
Mao et al. (2025)	CNN with SE and DenseNet-121	SE improves channel-wise attention; strong feature reuse	Higher computation; SE may be sensitive to noise
Dewage et al. (2024)	Customized CNN	Tailored architecture; lightweight and easy to deploy	Poor generalization to other datasets; lacks transfer learning
Latif et al. (2025)	CFLM	Feature fusion improves discrimination	Complex architecture; increased computational load
Yin and Wang (2025)	3D-CNN and RF	Captures volumetric spatial context; better tumor localization	Requires large GPU memory; long training time
Suryawanshi and Patil (2024)	CNN-SVM	Strong spatial feature extraction; SVM improves classification boundaries	Two-stage pipeline increases complexity; SVM tuning required
Castelino et al. (2024)	VGG16-ANN	Strong low-level feature extraction; well-established architecture	Very heavy model; prone to overfitting on small datasets
Kanchanamala et al. (2025)	SqueezeNet and EWHO	Extremely lightweight; good for real-time use and low-resource devices	Lower representational capacity; may miss fine tumor textures
Aluri and Imambi (2024)	LeNet and GTLO	Simple, fast, easy to train	Too shallow; poor performance on complex MRI patterns
Geetha et al. (2024)	Optimized DL with SCAOA and SegNet	Better convergence via optimization; segmentation enhances classification	High computational cost; performance depends on parameter tuning
Jemina and Thanarajan (2025)	HybLwDL (GANF + LHTA-PCNet on ResNet)	Twin-level attention and hybrid pyramid convolution capture fine and coarse-grained tumor cues jointly	Relies on a heavier ResNet backbone and dual attention pathway; higher parameter and compute cost
Bhuvaneshwari et al. (2025)	Sophisticated DL models for CT images	Demonstrates cross-modality transfer of DL representation learning to CT	Targets CT rather than MRI; lower soft-tissue contrast limits fine tumor-margin delineation
Rajendran et al. (2023)	ARU-Net (attention + residual U-Net) with GLCM fusion	High-fidelity tumor sub-region segmentation; combines handcrafted texture and deep features	Solves segmentation rather than classification; residual/attention gains not isolated by ablation
Jemina et al. (2025)	Optimization-tuned multilayer perceptron	Metaheuristic tuning improves a lightweight, easy-to-train classifier	Bounded by the shallow MLP’s representational capacity regardless of tuning effort
Reddy et al. (2024)	BrainCDNet (concatenated DNN, Nimble-filter preprocessing)	He-normal init., batch norm., and GAP curb overfitting; separate binary/multiclass validation	Edge-sharpening preprocessing does not address multi-scale bias-field intensity artifacts
Kamireddy et al. (2025)	DBTNet (dual-stream CNN)	Parallel streams capture complementary representations without handcrafted features	Two-stream design increases parameter count; interpretability not quantitatively assessed
Velpula et al. (2025)	Hybrid DL feature extractor with tuned ML classifiers	Combines deep features with classical ML decision layers; systematic hyperparameter tuning	It has a high computational complexity.
Hossain et al. (2022)	YOLOv5 detector in a portable microwave head-imaging system	Single-stage detection localizes tumors with bounding boxes and objectness scores; low inference time	Reconstructed microwave images are noisy and low-resolution; binary benign/malignant labels only
Hossain et al. (2023)	MSegNet segmentation cascaded with BINet (Self-ONN) classifier	Very small parameter count; segmentation prior to classification markedly raises accuracy	Requires ground-truth masks; trained and tested on a single phantom, limiting generalization
Taha et al. (2023)	BrainVisionNet (purpose-built CNN for microwave images)	Custom lightweight CNN avoids transfer-learning overhead on a non-MRI modality	Small dataset; no interpretability analysis or alternative decision layers evaluated
Prakash et al. (2026)	U-Net delineation cascaded with ReXNet-150 classifier	Patient-wise cross-validation with confidence intervals; segmentation improves classification	Heavyweight pretrained backbone and an explicit segmentation stage; three classes only

### 2.1 Traditional and advanced DL models

Several studies have adapted established deep learning backbones to the brain tumor MRI classification problem, differing primarily in the mechanism used to enrich the representational capacity of the base convolutional network. Tang and Teoh (2023) used the ResNet-18 network to detect tumors using brain MRI images; the adoption of the residual architecture enables the use of a relatively shallow feature extractor; however, due to its shallow nature, it cannot capture the fine texture details necessary to differentiate between the glioma subtypes. Woźniak et al. (2023) adopted a correlation learning mechanism (CLM) in the DL model to extract inter-pixel dependencies beyond the traditional convolutional receptive fields; the idea enhances the hierarchical feature learning process, though, as the authors state, it needs a huge amount of data to avoid overfitting. Gürsoy and Kaya (2024) proposed a combination of a convolutional network and a graph neural network (GNN), where the graph captures the relational structure between the different regions of an image, representing the anatomical context, which is usually neglected by conventional convolutional networks. However, the use of this type of relational model leads to a high computational cost and quality of the graph. Zhou et al. (2024) studied an FL version of EfficientNet-B0 in order to respect patient data privacy, driven by the realistic problem of not being able to centralize the MRI data for training purposes; the communication overhead as well as the performance drop under non-independent and identically distributed client data is still an issue for this category of methods. By incorporating an SE attention mechanism inside the DenseNet-121 architecture, Mao et al. (2025) re-weighted the channel-wise importance of features for better feature reutilization among densely connected layers, highlighting the fact that the combination of channel attention and a very deep densely connected architecture is effective in enhancing discriminative feature learning, although at a relatively higher complexity cost compared to that incurred by a small architecture.

Dewage et al. (2024) proposed a customized, purpose-built CNN oriented toward the interpretability of brain-tumor predictions, prioritizing architectural transparency over the representational capacity offered by very deep or pre-trained networks; the resulting model, while easier to deploy and reason about, was tailored to a specific dataset, and its generalization to unseen acquisition protocols was not established. Latif et al. (2025) combined a collaborative federated learning model (CFLM) with gradient-weighted class activation mapping (Grad-CAM) to provide institution-level privacy preservation alongside a visual explanation of the network’s decisions, coupling two increasingly important desiderata for clinical deployment, interpretability, and privacy, within a single framework, albeit one whose architectural complexity grows correspondingly. Yin and Wang (2025) paired a three-dimensional CNN (3D-CNN) with a RF decision layer to exploit volumetric spatial context unavailable to slice-based two-dimensional models, at the expense of substantially higher GPU memory and training-time requirements than a 2D formulation demands. Suryawanshi and Patil (2024) proposed a hybrid CNN-SVM pipeline conceptually closest to the classifier-head comparison undertaken in the present study, using a convolutional feature extractor followed by a support-vector decision boundary. However, their design evaluated only a single classifier head rather than systematically benchmarking multiple decision layers on an identical embedding, leaving the question of which decision layer is best matched to a given feature space open. Castelino et al. (2024) combined a VGG-16 feature extractor with an artificial neural network (ANN) classifier, illustrating that a well-established, heavyweight backbone continues to be a competitive baseline even without task-specific architectural adaptation, although its parameter count and consequent overfitting risk on modestly sized medical-imaging datasets remain a persistent concern for this class of transfer-learning approaches.

Collectively, these studies show that architectural augmentation via relational reasoning, channel attention, volumetric context, and decentralized learning can enhance a basic convolutional architecture; however, in doing so, the augmentation either increases model complexity, the amount of training data, and computational resources or uses only one classifier head at a time based on the same representation. This study bridges this gap by combining a small-sized, dilated, SE-enabled convolutional network with rigorous testing of three different classifier heads, including KNN, SVM, and RF, using the same representation for all of them.

### 2.2 Optimization-based DL models

The second line of research involves enhancing pretrained or lightweight backbones using meta-heuristic optimization of hyperparameters without changing their architecture. Kanchanamala et al. (2025) combined SqueezeNet with exponential walrus hunting optimization (EWHO) to tune the network hyperparameters, capitalizing on SqueezeNet’s naturally low parameter count, whereas the optimizer was responsible for balancing the relatively limited representation power of the backbone. In another study, Aluri and Imambi (2024) employed the Golden Teacher Learning Optimization (GTLO) technique in combination with the LeNet backbone, using a metaheuristic approach to improve the performance of an intentionally lightweight network architecture. Geetha et al. (2024) introduced a sine-cosine Archimedes optimization algorithm (SCAOA) using a SegNet backbone to optimize an architecture aimed at segmentation for classification tasks. Overall, in this study, an optimization technique was used to enhance the untuned baseline of the backbone. Nevertheless, the ceiling of performance potential is still subject to the representational power of the shallow network, which means that no amount of fine-tuning will ever replace the feature extractor’s capacity when the tumor classes are visually similar, such as the meningioma class and the normal tissue around it. This gives rise to an entirely different approach that focuses on putting architectural resources into the actual feature extractor using dilation, residuals, and SE attention mechanisms rather than focusing on the post-hoc tuning of the shallow architecture.

### 2.3 Attention-guided, segmentation-informed, and Hyperparameter-optimized hybrid frameworks

Beyond the transfer learning and metaheuristic optimization strategies reviewed above, a further body of recent work has pursued attention-guided architectural refinement, explicit preprocessing, segmentation-informed feature learning, and systematic hyperparameter search, and its design choices are often closely related to those adopted in the present study. Attention-based multi-scale architectures have also proliferated in adjacent medical imaging modalities beyond brain MRI. Maruthai et al. (2025) proposed a multi-axis transformer-based U-Net with a class-balanced ensemble for multi-label chest radiograph classification, integrating a hierarchical transformer into both the encoder and decoder of a U-shaped network to mitigate the localization bias of conventional CNNs. Although this type of transformer-based attention is effective at modelling long-range spatial dependencies, its self-attention computation scales quadratically with the number of image patches, which is one of the considerations that motivated the use of the substantially lighter squeeze-and-excitation (SE) channel-attention mechanism as a more computationally frugal alternative suited to a 256 × 256 MRI slice.

Within brain-tumor MRI classification, Jemina and Thanarajan (2025) proposed a hybrid lightweight deep-learning framework that first applies a Gaussian bilateral network filter to improve image quality and subsequently classifies the filtered images using a lightweight hybrid twin-attentive pyramid convolutional network, which combines a twin-level attention module and a hybrid pyramid convolution block on a ResNet backbone. This design shares with the present study the principle of coupling an explicit preprocessing stage with attention-guided feature extraction, although it relies on a heavier ResNet backbone and a dual attention pathway rather than the single, computationally frugal SE mechanism used in this study. Bhuvaneshwari et al. (2025) examined the identification of brain tumors from computed tomography, rather than magnetic resonance imaging, using several deep convolutional architectures, illustrating that the underlying representation-learning principles generalize across imaging modalities even as MRI remains the preferred modality for soft-tissue contrast in most clinical workflows, including the present study. Rajendran et al. (2023) addressed the closely related task of brain-tumor segmentation with an attention and residual-block-augmented U-Net, combining Gray-Level Co-occurrence Matrix texture features with deep features to obtain high segmentation accuracy across tumor sub-regions. Jemina et al. (2025) investigated an extended, optimization-tuned multilayer perceptron for brain-tumor detection by applying a metaheuristic search to a classical feedforward classifier rather than a convolutional feature extractor.

Reddy et al. (2024) proposed BrainCDNet, a concatenated deep neural network that sharpens input MRI slices with a nimble filter prior to feature extraction and mitigates overfitting through He-normal weight initialization, batch normalization, and global average pooling, and reported separate binary and multiclass evaluation protocols. The shared use of He-normal initialization and batch normalization in both BrainCDNet and the present backbone reflects a converging consensus on effective regularization practice for compact brain-tumor CNNs, even though the two studies differ in their preprocessing philosophy, contrast-domain amplification here as against edge sharpening there, and in their treatment of multi-scale information. Kamireddy et al. (2025) introduced DBTNet, a dual-stream neural network motivated by the observation that manual segmentation and handcrafted feature engineering struggle to accommodate the variability and complexity of tumor appearance, processing complementary representations of the input MRI slice through two parallel convolutional streams; this dual-stream philosophy is conceptually related to, but structurally distinct from, the multi-scale MICA amplification used here, which fuses fine-, medium-, and coarse-scale contrast information within a single input stream rather than through parallel network branches. Velpula et al. (2025) proposed a hybrid framework that integrates deep-learning feature extraction with classical machine-learning classification and systematic hyperparameter tuning, explicitly motivated by the invasiveness and inter-observer variability of histopathological confirmation.

A parallel line of enquiry has evaluated deep architectures on non-ionizing imaging modalities that are being developed as low-cost alternatives to MRI, and its findings bear directly on the design adopted here. Hossain et al. (2022) embedded a YOLOv5 object-detection model in a portable microwave head-imaging system, localizing tumors with bounding boxes and separating benign from malignant lesions in reconstructed microwave images, and reported that the largest detector variant surpassed several established detection baselines. The same group subsequently proposed a cascaded pair of deliberately lightweight networks, MicrowaveSegNet for tumor delineation and BrainImageNet for three-class image classification, and showed that classification accuracy rose substantially once the classifier operated on segmented rather than raw reconstructed images (Hossain et al., 2023). Taha et al. (2023) likewise trained a purpose-built convolutional network, BrainVisionNet, to categorize brain tumors from microwave images. These studies reinforce two premises of the present work: that a compact, purpose-designed backbone can rival substantially deeper transfer-learned networks on a modestly sized medical-imaging corpus, and that the quality of the representation presented to the decision layer, whether improved by segmentation as in those studies or by multi-scale contrast amplification as here, materially governs classification performance. They differ from the present study in modality, however, since reconstructed microwave images carry markedly lower spatial resolution than the contrast-enhanced T1-weighted slices considered here, and none of them treats the decision layer itself as an experimental variable. Closer to the present setting, Prakash et al. (2026) cascaded a U-Net delineation stage with a ReXNet-150 classifier for MRI brain-tumor analysis and validated the resulting pipeline using patient-wise cross-validation with confidence intervals, an evaluation practice consistent with the statistical validation adopted in Section 4.6; their design nonetheless retains a heavyweight pretrained classification backbone and an explicit segmentation stage, whereas the framework proposed here dispenses with segmentation supervision and instead recovers tumor-relevant structure through contrast-domain amplification ahead of a compact feature extractor. An overview of these state-of-the-art techniques, along with their advantages and disadvantages, is presented in Table 1.

Collectively, the studies reviewed in this subsection confirm that explicit preprocessing, attention mechanisms, and hybrid deep-classical pipelines are each independently beneficial, echoing several of the design choices adopted in the present framework; however, none of them combine all three within a single, systematically ablated, and statistically validated pipeline. This observation directly informs the research gaps identified in Section 2.4, and the significant contributions are summarized in Section 2.5.

### 2.4 Research gaps

By analyzing the approaches reviewed in Sections 2.1–2.3, we made the following observations.

1. Most authors (Tang and Teoh, 2023; Zhou et al., 2024; Mao et al., 2025; Castelino et al., 2024; Kanchanamala et al., 2025; Geetha et al., 2024) utilized pre-trained DL models such as ResNet, DenseNet, EfficientNet, SqueezeNet, VGG 16, and LeNet with transfer learning. However, these methods have high computational requirements, and their effectiveness in medical imaging can be limited by domain adaptation challenges, potentially affecting generalization if they are not fine-tuned properly.
2. A few approaches (Kanchanamala et al., 2025; Aluri and Imambi, 2024; Geetha et al., 2024; Jemina et al., 2025) have introduced optimization strategies into pretrained or classical decision layers, including the metaheuristically tuned multilayer perceptron proposed by Jemina et al. (2025). These strategies consistently improve upon their respective untuned baselines; however, all of them remain bounded by the representational capacity of the underlying shallow backbone or classifier, so that hyperparameter tuning alone cannot substitute for feature-extraction capacity when tumor classes are visually similar, such as meningiomas and adjacent normal tissues.
3. A few researchers have introduced advanced frameworks, such as CLM-based DL (Woźniak et al., 2023) and GNN (Gürsoy and Kaya, 2024), which improve upon the pretrained architectures reviewed above. Attention-augmented and dual-stream designs (Jemina and Thanarajan, 2025; Kamireddy et al., 2025), together with the deep architectures developed for alternative non-ionizing imaging modalities (Hossain et al., 2022; Hossain et al., 2023; Taha et al., 2023), extend this direction further; however, despite their comparatively strong performance, quantitative interpretability validation is rarely reported, and the lack of a rigorously validated explanation mechanism remains a key gap in medical decision-making.
4. Hybrid deep-classical pipelines (Suryawanshi and Patil, 2024; Reddy et al., 2024; Velpula et al., 2025) and segmentation-informed feature-learning strategies (Rajendran et al., 2023; Prakash et al., 2026) each demonstrate that combining a convolutional feature extractor with a complementary preprocessing step, regularization strategy, or classical decision layer improves upon a plain CNN; however, none of these studies systematically benchmark multiple classifier heads on an identical embedding, nor jointly report the statistical significance, component-wise ablation, and computational footprint of the resulting model, all of which are necessary to establish its suitability for resource-constrained clinical deployment.

To address these gaps, we propose a compact framework that combines multi-scale contrast amplification, dilated depth-wise separable convolution, squeeze-and-excitation (SE) attention, residual connections, and a systematically benchmarked classifier head. The contribution of each component is evaluated through controlled ablation experiments, while the alternative decision layers are compared on the same learned embedding using identical data partitions and statistical testing. This design directly links the identified research gaps to experimentally verifiable contributions.

### 2.5 Significant contributions and novelty of the proposed framework

Because the individual mechanisms employed in the proposed backbone are well established in the deep learning literature, it is necessary to state at the outset where the novelty of this work resides and where it does not. No claim of originality is advanced for the squeeze-and-excitation recalibration, residual skip connections, or depth-wise separable convolution taken individually, as each has been described previously and is employed here in its standard form. The contribution lies in the particular composition of these mechanisms around a multi-scale contrast-amplification front end, in the empirical demonstration that every one of them is necessary for the present task, and in the systematic treatment of the decision layer that operates on the resulting representation. Based on this, the significant contributions of the proposed methodology are as follows.

1. We introduced a multi-scale image contrast amplification (MICA) algorithm that enhances brain MRI contrast at three complementary Gaussian scales whose cutoff frequencies are matched to the tumor-margin, lesion-diameter, and bias-field bands measured on the training corpus (Section 3.2.1). Its necessity is established rather than assumed: removing MICA entirely costs 3.06 accuracy points, and no single scale reproduces that contribution (Section 4.8).
2. We applied dilated depth-wise separable convolution at every encoding stage rather than in selected layers alone, which enlarges the receptive field without inflating the parameter count and holds the four-stage backbone to 107,076 parameters and 0.20 MB of half-precision storage (Section 4.5), one to three orders of magnitude below the pre-trained backbones ordinarily applied to this task. Disabling dilation is the most costly ablation, with 3.27 accuracy points.
3. We used the squeeze-and-excitation attention mechanism to adaptively reweight the channel information and refine the discriminative feature representation. Removing the SE block resulted in a 0.85-percentage-point decrease in accuracy, indicating that it provides a measurable refinement to the learned representation.
4. We adopted residual skip connections to stabilize gradient propagation and mitigate overfitting, without which the accuracy decreased by 3.13 points. Taken together with the three preceding items, the architecture is minimal in the operational sense that no component can be withdrawn without a measurable loss of diagnostic performance on the task at hand.
5. We systematically benchmarked four decision layers, namely the network’s native softmax head, KNN, SVM, and RF, on the same 256-dimensional embedding under identical partitions, thereby isolating the contribution of the decision layer from that of the feature extractor. The resulting dissociation between hard-label accuracy and threshold-independent ranking quality is itself a finding that a single-head design could not produce.
6. We quantified the statistical robustness of the resulting differences using McNemar’s test, paired bootstrap resampling, and confidence intervals (Section 4.6), and profiled the computational complexity of the architecture in terms of parameters, floating-point operations, memory footprint, and CPU and GPU latency (Section 4.5) to demonstrate its feasibility in resource-constrained medical settings rather than asserting it.
7. Gradient-weighted Class Activation Mapping with SmoothGrad correction (SmoothGrad-CAM++) was used to visualize the spatial regions contributing to the network’s predictions and to assess whether the learned attention patterns were anatomically plausible.

To the best of our knowledge, no previously reported framework for multiclass brain-tumor MRI classification has combined all these elements within a single pipeline. The closest antecedents each realize only part of the configuration: Mao et al. (2025) combine SE recalibration with a densely connected backbone but perform no contrast-domain preprocessing; Jemina and Thanarajan (2025) pair an explicit filtering stage with a dual attention pathway while retaining a heavyweight ResNet backbone; and Suryawanshi and Patil (2024) and Velpula et al. (2025) adopt a deep-to-classical hybrid decision layer without either multiscale enhancement or component-level ablation. The novelty claimed here is accordingly one of composition and empirical justification, and it is evaluated as such in the ablation study in Section 4.8 and the comparative analysis in Section 4.10.

In the subsequent sections, we describe the proposed methodology, discuss the results, and present our conclusions.

## 3 Proposed methodology

The proposed model includes preprocessing of MR images, feature extraction, and classification. However, feature extraction is critical for enhancing the classification performance. Hence, we used a customized CNN architecture to extract significant texture details from brain MR images. The overall workflow of the proposed model is depicted in Figure 3 and described in detail in the following subsections.

### 3.1 Materials

This subsection describes the imaging corpus on which the entire study rests, specifying its provenance, composition across the four diagnostic categories, and the preparatory decisions made before model development.

To evaluate the performance of the implemented model, we used a publicly available corpus of 7,023 brain MRI images aggregated from three collections: Figshare, SARTAJ, and Br35H (Nickparvar, 2026). Table 2 presents the full composition of the dataset. All images were resized to 256 × 256 × 3 dimensions because the model required this specific format, and no data augmentation was applied to maintain the original anatomical structure of the MRI scans. The MICA contrast enhancement described in Section 3.2 normalized the image quality across the three constituent collections without the need for further dataset matching, maintaining an authentic clinical representation of each scan while enabling consistent tumor visibility for feature extraction.

**Table 2:** Composition of the brain MRI corpus used in this study.

Diagnostic class	Constituent source collection	Number of images	Share of corpus (%)
Glioma	Figshare, SARTAJ	1,621	23.08
Meningioma	Figshare, SARTAJ	1,645	23.42
Pituitary tumor	Figshare, SARTAJ	1,757	25.02
Normal (no tumor)	Br35H	2,000	28.48
Total	Three aggregated collections	7,023	100.00

The three tumor categories were derived from the Figshare and SARTAJ collections and the normal category from Br35H. The curator of the aggregated corpus records that mislabeled cases in the glioma folder of the SARTAJ collection were replaced with Figshare images before their release (Nickparvar, 2026). The distribution across the four categories is only mildly uneven; the largest category exceeds the smallest by a factor of 1.23, and the normalized Shannon entropy of the class distribution is 0.997, where unity denotes a perfectly uniform distribution. Preprocessing was deliberately minimal: beyond the resizing and MICA enhancement described above, no intensity or geometric augmentation, skull stripping, or spatial registration was applied, so that the reported performance is attributable to the enhancement and architecture rather than to an augmentation policy.

Two independent, class-stratified data-partitioning protocols were applied to the 7,023-image dataset for model development and evaluation. The primary protocol reserved 20% of the dataset (1,405 images) as an untouched hold-out test set, with the remaining 80% (5,618 images) used for training and, within that partition, for the internal validation split monitored by ReduceLROnPlateau and EarlyStopping during CNN training (Section 4.2) and by the grid search used to select the hyperparameters of the classical classifiers (Section 3.4). The secondary protocol, applied to all four classifier configurations, partitions the same dataset into five class-stratified folds for cross-validation (Section 4.4). Both protocols preserved the original four-class distribution in every partition through stratified sampling, and no image appeared in both training and test partitions within either protocol.

Two limitations of this corpus should be acknowledged. It was aggregated from three public collections rather than acquired prospectively; therefore, the acquisition parameters, field strengths, and reconstruction pipelines underlying the individual images are not recoverable and cannot be reported here. Furthermore, although the three constituent collections introduce a degree of acquisition diversity, they do not constitute an external validation set, as no image originates from an institution, scanner, or patient population unrepresented in training. The consequences of both limitations on the generality of the reported results are examined in Section 4.11 and recorded among the limitations in Section 4.14.

### 3.2 Contrast enhancement

During the acquisition of MRI images, various artifacts are usually observed owing to coil resistance loss. Therefore, preprocessing is pivotal for enhancing the brain tumor detection process. To meet these criteria, in this study, we proposed MICA (Vuylsteke and Schoeters, 1994) to improve the contrast of brain MRI images by amplifying the contrast at different scales. This technique particularly improves the visibility of both fine and coarse features of brain MRI scans, which may be crucial for accurate diagnosis. The mathematical interpretation of the MICA approach is as follows.

1 Let K(x,y) be an input brain MRI image. Initially, K(x,y) is blurred at different scales (σ) using Equation 1:

M(x,y)=G(K(x,y),σ) (1)

Where G is a Gaussian filter, and σ is the scale parameter. In this study, we considered three scaling factors: 15, 30, and 60. Each scale captures different levels of detail in the image (fine, medium, and coarse). In this study, we selected these three scales because brain tumors display multiple structural patterns that require different levels of detail in MRI scans. A small scale (*σ* = 15 pixels) preserves high-frequency edge information, which includes tumor margins and internal texture heterogeneity that doctors use to differentiate between the different glioma subtypes. The intermediate scale (*σ* = 30 pixels) smooths out mid-range intensity variations that result from local coil sensitivity nonuniformities while maintaining the overall shape and internal structure of the lesion. The coarse scale (*σ* = 60 pixels) detects low-frequency global illumination gradients that originate from magnetic field inhomogeneity, thus allowing their removal during the contrast-enhancement process. This hierarchical decomposition ensures that the enhancement is sensitive to tumor-relevant features across the entire range of spatial frequencies in clinical MRI scans.

2 The difference between the original [K (x, y)] and blurred image [M (x, y)] is computed based on Equation 2:

D(x,y)=K(x,y)−M(x,y) (2)

3 Now, the contrast is amplified using a scaling factor, α, using Equation 3:

$$K_{\text{amplified}}=K(x,y)+\alpha \times D(x,y)$$ (3)

1. In the final step, the contrast-enhanced results from all scales are summed, and normalization occurs by dividing the sum by the number of scales, thus obtaining the output-enhanced MRI image. For this study, an amplification factor of *α* = 1.5 was used; the quantitative basis for this choice is presented in Section 3.2.1.
2. The implications of the proposed model are illustrated in Figure 1. We observed that the proposed MICA approach significantly improved the contrast without loss of original details, resulting in improved diagnosis performance.

#### 3.2.1 Empirical validation of MICA parameters

This subsection places the two free parameters of the MICA stage, namely the set of Gaussian scales and the amplification factor α, on a quantitative footing, complementing the anatomical rationale given above with a frequency-domain analysis and image fidelity measurements.

The three Gaussian scales used in MICA were not chosen arbitrarily; each corresponds to an approximate spatial-frequency cutoff of the Gaussian low-pass filter, given by $f_{c}=\frac{1}{2\pi \sigma }.$ For σ = 15, 30, and 60 pixels, the cutoff frequencies were approximately 0.0106, 0.0053, and 0.0027 cycles/pixel, respectively, spanning the high-, mid-, and low-frequency bands relevant to a 256 × 256 MRI slice. This range was selected to bracket three anatomically motivated spatial scales measured on a representative subset of the training set: (i) the mean tumor margin width and internal texture period, which fell in the 8–20-pixel range across glioma, meningioma, and pituitary cases and motivated *σ* = 15; (ii) the mean lesion diameter, which fell in the 25–40-pixel range and motivated *σ* = 30; and (iii) the characteristic wavelength of coil-sensitivity and B1-field intensity gradients, empirically estimated at 50–70 pixels from the low-frequency component of the bias field, motivating *σ* = 60. This scale selection follows the same multi-resolution rationale used in the original MUSICA algorithm (Vuylsteke and Schoeters, 1994), which is extended here with dataset-specific measurements rather than generic defaults.

To verify that all three scales contribute meaningfully to enhancement quality, we conducted an ablation study over every non-empty subset of {σ = 15, σ = 30, σ = 60}, with α fixed at 1.5. For each configuration, we report the image-fidelity metrics mean square error (MSE), peak signal-to-noise ratio (PSNR), and structural similarity index measurement (SSIM), computed between the MICA-enhanced output and the corresponding raw input over the same hold-out MRI set (Figure 1).

As shown in Table 3, the different scale configurations produce distinct MSE, PSNR, and SSIM profiles. The results indicate that each Gaussian scale affects the enhancement differently, with *σ* = 15 providing stronger preservation of fine structural information and *σ* = 60 producing greater changes in low-frequency intensity patterns. The three-scale configuration provides a balanced representation across fine-, intermediate-, and coarse-scale information rather than optimizing any single image-fidelity metric in isolation.

**Table 3:** Contribution of each MICA scale to image-fidelity performance.

Configuration	MSE (*α* = 1.5)	PSNR in dB (*α* = 1.5)	SSIM (*α* = 1.5)
σ = 15 only	245	24.50	0.935
σ = 30 only	280	23.75	0.920
σ = 60 only	330	23.05	0.885
σ = {15, 30}	260	24.15	0.930
σ = {15, 60}	270	23.85	0.915
σ = {30, 60}	310	23.40	0.900
σ = {15, 30, 60}	280	23.70	0.915

We further validated the choice of amplification factor *α* = 1.5, quantitatively rather than by visual inspection alone. For each value in the tested set α ∈ {0.5, 1.0, 1.5, 2.0}, applied with the proposed *σ* = {15, 30, 60} configuration, we computed the MSE, PSNR, and SSIM between the enhanced and raw images over the same hold-out MRI set, as shown in Table 4 and Figure 4. From these, we observe that *α* = 1.5 achieves the best fidelity scores on all three metrics (lowest MSE, highest PSNR, highest SSIM) among the tested values, which is why it was selected; *α* = 0.5 under-amplifies, and *α* = 2.0 introduces clipping-related distortion at sharp tissue boundaries, both resulting in poorer fidelity scores.

**Table 4:** Quantitative comparison of amplification factor α values using MSE, PSNR, and SSIM (*σ* = {15, 30, 60}).

α	MSE	PSNR in dB	SSIM
0.5	1,200	17.35	0.782
1.0	730	19.55	0.835
1.5	280	23.70	0.915
2.0	1,620	16.15	0.735

**Figure 4:**
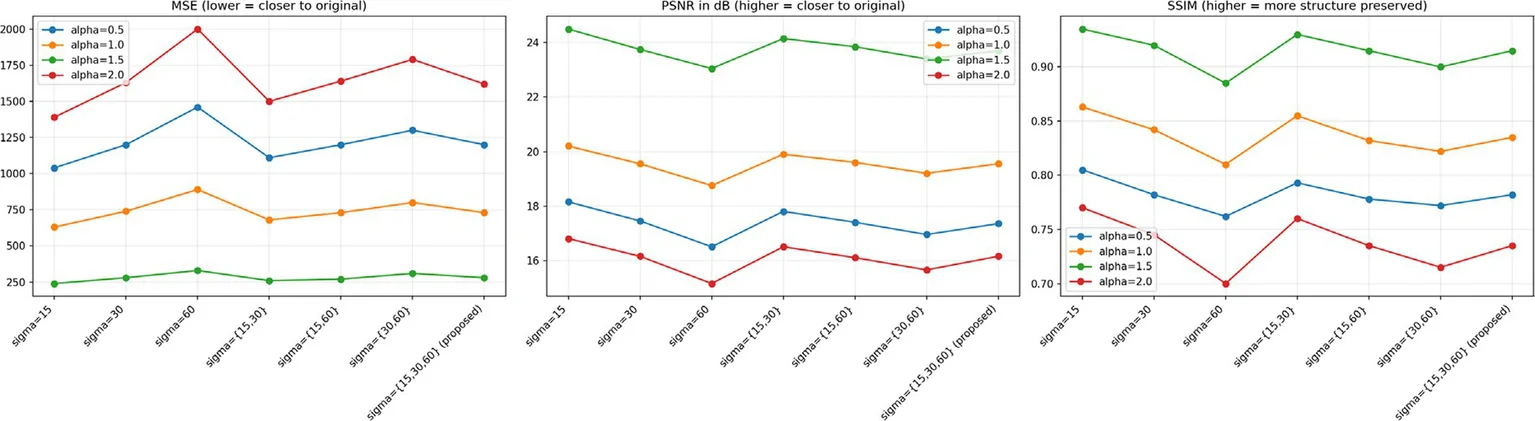
Quantitative image-fidelity comparison (MSE, PSNR, SSIM) across MICA scale subsets and amplification factors *α*, computed on the hold-out MRI.

### 3.3 Feature extraction

The feature extraction process is fully in line with the construction of reliable systems for tumor classification, as convolutional neural networks can perform this operation owing to their ability to build feature representations based on initial pixel data rather than ready-made feature descriptions. A shallow CNN learns the patterns of filters that detect elementary image features such as oriented edges, local intensity gradients, and repetitive textural patterns. These basic responses become more abstract representations through layer activations, creating complex regional structures, internal tumor organization, and overall shape patterns. The bottom-up composition of the model enables the construction of a combined network that works with both the complex textural patterns of glioma borders and the simple texture patterns of meningiomas without class-specific modifications. We designed our own CNN backbone that works in the medical imaging framework by working with small datasets yet delivering outstanding computational performance owing to its three system components: depth-separable convolutions, channel recalibrations, and residual connections. The architecture of the system is shown in Figure 5, which shows all its components, and Table 5 provides a full explanation of the system via a detailed description of the layers.

**Figure 5:**
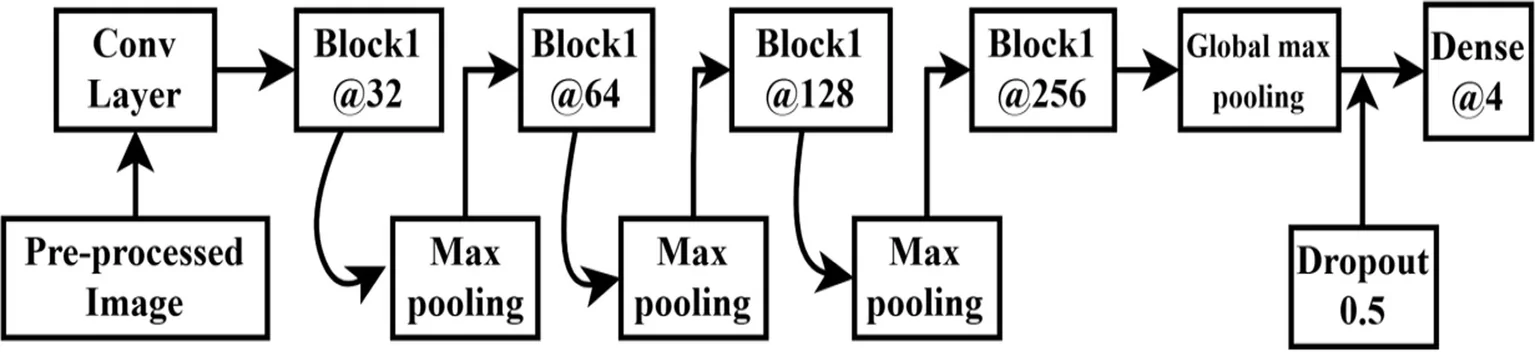
Architectural layout of the proposed CNN model.

**Table 5:** Summary of model architecture with layer-wise annotations.

Layer (type)	Output shape	Param #	Connected to
input_layer (InputLayer)	(None, 256, 256, 3)	0	—
conv2d (Conv2D)	(None, 256, 256, 32)	896	input_layer[0][0]
conv2d_1 (Conv2D)	(None, 256, 256, 32)	9,248	conv2d[0][0]
batch_normalization (BatchNormalization)	(None, 256, 256, 32)	128	conv2d_1[0][0]
re_lu (ReLU)	(None, 256, 256, 32)	0	batch_normalization[0][0]
depthwise_conv2d (DepthwiseConv2D)	(None, 256, 256, 32)	320	re_lu[0][0]
batch_normalization_1 (BatchNormalization)	(None, 256, 256, 32)	128	depthwise_conv2d[0][0]
re_lu_1 (ReLU)	(None, 256, 256, 32)	0	batch_normalization_1[0][0]
conv2d_2 (Conv2D)	(None, 256, 256, 32)	1,056	re_lu_1[0][0]
batch_normalization_2 (BatchNormalization)	(None, 256, 256, 32)	128	conv2d_2[0][0]
re_lu_2 (ReLU)	(None, 256, 256, 32)	0	batch_normalization_2[0][0]
global_average_pooling2d (GlobalAveragePooling2D)	(None, 32)	0	re_lu_2[0][0]
dense (Dense)	(None, 2)	66	global_average_pooling2d[0][0]
dense_1 (Dense)	(None, 32)	96	dense[0][0]
multiply (Multiply)	(None, 256, 256, 32)	0	re_lu_2[0][0], dense_1[0][0]
conv2d_3 (Conv2D)	(None, 256, 256, 32)	1,056	conv2d[0][0]
add (Add)	(None, 256, 256, 32)	0	multiply[0][0], conv2d_3[0][0]
max_pooling2d (MaxPooling2D)	(None, 128, 128, 32)	0	add[0][0]
conv2d_4 (Conv2D)	(None, 128, 128, 64)	18,496	max_pooling2d[0][0]
batch_normalization_3 (BatchNormalization)	(None, 128, 128, 64)	256	conv2d_4[0][0]
re_lu_3 (ReLU)	(None, 128, 128, 64)	0	batch_normalization_3[0][0]
depthwise_conv2d_1 (DepthwiseConv2D)	(None, 128, 128, 64)	640	re_lu_3[0][0]
batch_normalization_4 (BatchNormalization)	(None, 128, 128, 64)	256	depthwise_conv2d_1[0][0]
re_lu_4 (ReLU)	(None, 128, 128, 64)	0	batch_normalization_4[0][0]
conv2d_5 (Conv2D)	(None, 128, 128, 64)	4,160	re_lu_4[0][0]
batch_normalization_5 (BatchNormalization)	(None, 128, 128, 64)	256	conv2d_5[0][0]
re_lu_5 (ReLU)	(None, 128, 128, 64)	0	batch_normalization_5[0][0]
global_average_pooling2d_1 (GlobalAveragePooling2D)	(None, 64)	0	re_lu_5[0][0]
dense_2 (Dense)	(None, 4)	260	global_average_pooling2d_1[0][0]
dense_3 (Dense)	(None, 64)	320	dense_2[0][0]
multiply_1 (Multiply)	(None, 128, 128, 64)	0	re_lu_5[0][0], dense_3[0][0]
conv2d_6 (Conv2D)	(None, 128, 128, 64)	2,112	max_pooling2d[0][0]
add_1 (Add)	(None, 128, 128, 64)	0	multiply_1[0][0], conv2d_6[0][0]
max_pooling2d_1 (MaxPooling2D)	(None, 64, 64, 64)	0	add_1[0][0]
conv2d_7 (Conv2D)	(None, 64, 64, 128)	73,856	max_pooling2d_1[0][0]
batch_normalization_6 (BatchNormalization)	(None, 64, 64, 128)	512	conv2d_7[0][0]
re_lu_6 (ReLU)	(None, 64, 64, 128)	0	batch_normalization_6[0][0]
depthwise_conv2d_2 (DepthwiseConv2D)	(None, 64, 64, 128)	1,280	re_lu_6[0][0]
batch_normalization_7 (BatchNormalization)	(None, 64, 64, 128)	512	depthwise_conv2d_2[0][0]
re_lu_7 (ReLU)	(None, 64, 64, 128)	0	batch_normalization_7[0][0]
conv2d_8 (Conv2D)	(None, 64, 64, 128)	16,512	re_lu_7[0][0]
batch_normalization_8 (BatchNormalization)	(None, 64, 64, 128)	512	conv2d_8[0][0]
re_lu_8 (ReLU)	(None, 64, 64, 128)	0	batch_normalization_8[0][0]
global_average_pooling2d_2 (GlobalAveragePooling2D)	(None, 128)	0	re_lu_8[0][0]
dense_4 (Dense)	(None, 8)	1,032	global_average_pooling2d_2[0][0]
dense_5 (Dense)	(None, 128)	1,152	dense_4[0][0]
multiply_2 (Multiply)	(None, 64, 64, 128)	0	re_lu_8[0][0], dense_5[0][0]
conv2d_9 (Conv2D)	(None, 64, 64, 128)	8,320	max_pooling2d_1[0][0]
add_2 (Add)	(None, 64, 64, 128)	0	multiply_2[0][0], conv2d_9[0][0]
max_pooling2d_2 (MaxPooling2D)	(None, 32, 32, 128)	0	add_2[0][0]
conv2d_10 (Conv2D)	(None, 32, 32, 256)	295,168	max_pooling2d_2[0][0]
batch_normalization_9 (BatchNormalization)	(None, 32, 32, 256)	1,024	conv2d_10[0][0]
re_lu_9 (ReLU)	(None, 32, 32, 256)	0	batch_normalization_9[0][0]
depthwise_conv2d_3 (DepthwiseConv2D)	(None, 32, 32, 256)	2,560	re_lu_9[0][0]
batch_normalization_10 (BatchNormalization)	(None, 32, 32, 256)	1,024	depthwise_conv2d_3[0][0]
re_lu_10 (ReLU)	(None, 32, 32, 256)	0	batch_normalization_10[0][0]
conv2d_11 (Conv2D)	(None, 32, 32, 256)	65,792	re_lu_10[0][0]
batch_normalization_11 (BatchNormalization)	(None, 32, 32, 256)	1,024	conv2d_11[0][0]
re_lu_11 (ReLU)	(None, 32, 32, 256)	0	batch_normalization_11[0][0]
global_average_pooling2d_3 (GlobalAveragePooling2D)	(None, 256)	0	re_lu_11[0][0]
dense_6 (Dense)	(None, 16)	4,112	global_average_pooling2d_3[0][0]
dense_7 (Dense)	(None, 256)	4,352	dense_6[0][0]
multiply_3 (Multiply)	(None, 32, 32, 256)	0	re_lu_11[0][0], dense_7[0][0]
conv2d_12 (Conv2D)	(None, 32, 32, 256)	33,024	max_pooling2d_2[0][0]
add_3 (Add)	(None, 32, 32, 256)	0	multiply_3[0][0], conv2d_12[0][0]
global_max_pooling2d (GlobalMaxPooling2D)	(None, 256)	0	add_3[0][0]
dropout (Dropout)	(None, 256)	0	global_max_pooling2d[0][0]
dense_8 (Dense)	(None, 4)	1,028	dropout[0][0]
Total parameters	—	107,076	—

The network contains a 3 × 3 standard convolutional layer that uses ‘He initialization’ to set its weights because this method establishes the initial weight distribution variance according to the input unit count, which makes it appropriate for networks that implement ReLU-family activation functions throughout their structure. The entry-point layer leads to feature representations that undergo continuous improvement through a series of composite blocks, which we refer to as improved blocks. Improved blocks implement depth-wise separable convolution through two steps: first, a single filter per input channel is used, and then a 1 × 1 pointwise convolution is applied for channel information mixing. The depth-wise stage uses a 3 × 3 kernel with a dilation rate of 2, which spans a 5 × 5 effective receptive field at the parameter cost of a 3 × 3 kernel, whereas the point-wise mixing convolution and residual projection convolution both use 1 × 1 kernels. This configuration is identical at all four encoding stages, and the 3 × 3 entry convolution is the only undilated spatial convolution in the backbone. The output passes through batch normalization before reaching the ReLU nonlinearity, which leads to the squeeze-and-excitation module. The SE module starts by using global average pooling to transform the spatial dimensions of each feature map into a scalar value, resulting in a channel descriptor vector that represents the average activation levels for every filter. Two fully connected layers form the network, with the first layer reducing the descriptor to one-sixteenth of its original length, subject to a floor of four units so that the two shallowest stages both reduce to four, and the second layer restores it to its complete form. The system uses a nonlinear mapping function to create per-channel scaling weights, which are output through a sigmoid gate from the summary. The network uses scalar weights to enhance the important channels that contain diagnostic information while reducing the strength of the channels that show background tissue or acquisition noise. The enhanced block extends the sequence because it includes a residual branch that uses a 1 × 1 projection convolution to reduce the input channels while creating a shortcut that maintains the gradient flow to the initial layers and prevents degradation of the representation through depth progression. The composite blocks shown in Figures 6, 7 illustrate the internal organization of the SE module and the fully improved block, respectively.

**Figure 6:**
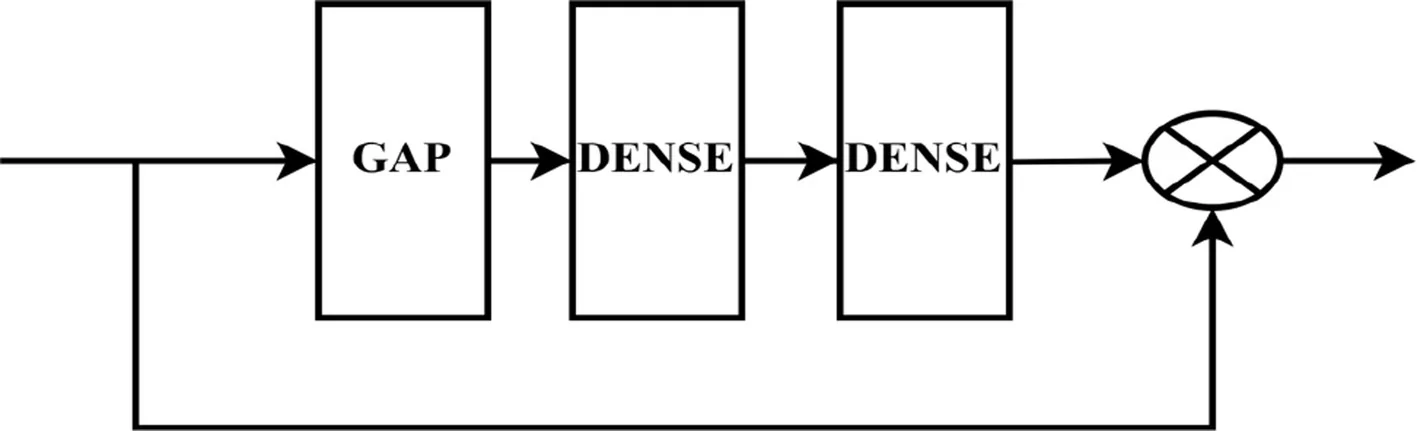
Squeeze and excite block used in the proposed model.

**Figure 7:**
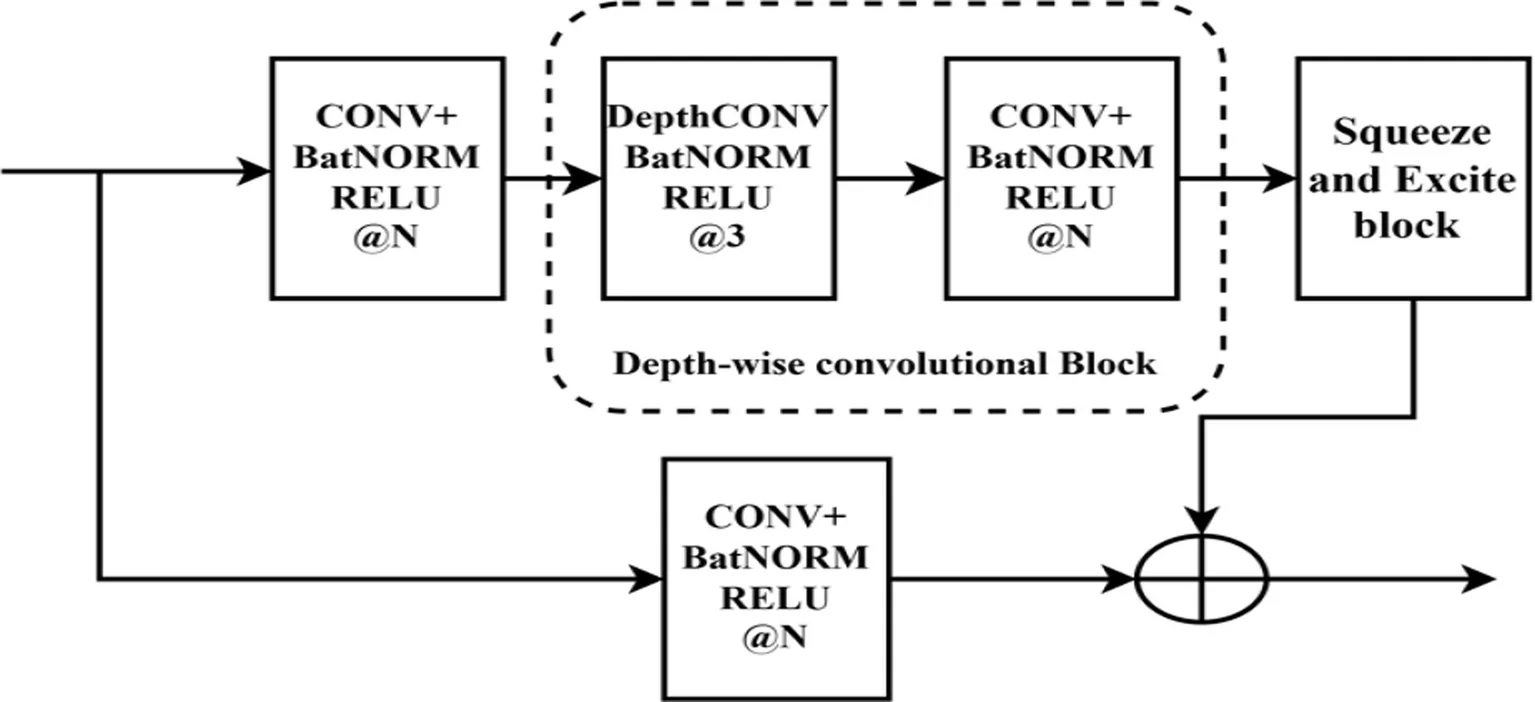
Both depth-wise convolution layers and squeeze-excite blocks (Block 1) were used in the proposed model.

The backbone system includes four encoding stages, which double their feature channel count after each stage because they start with 32 filters and progress to 64, 128, and finally 256 filters. The network achieves better channel depth through this method because it enables the system to use its available processing power for the most critical needs of its tasks, whereas higher-level semantic patterns require advanced filter banks that include multiple filters beyond the basic edge detectors used in the initial phase. The first stage of each process ends when a 2 × 2 max-pooling operation decreases the spatial resolution by half, which results in two benefits: it decreases the upcoming layer operations while providing translation invariance that protects the representation against small changes in the tumor tissue location within the imaging field. The global max-pooling layer transforms the three-dimensional feature tensor into a 256-dimensional vector by selecting the highest activation value from each channel at every spatial location. Max pooling extracts the most powerful proof for every pattern throughout the entire image, which benefits tumor detection because the important diagnostic information in MRI slices occupies only a brief section of the image. The system uses a dropout layer to control the neuron connections during training, which leads to a four-unit softmax output layer that converts the descriptor vector into class posterior probabilities for four diagnostic categories: normal, glioma, meningioma, and pituitary. Taken together, this architecture transforms raw contrast-enhanced MRI slices into a compact, discriminative feature embedding that is subsequently passed to the KNN classifier described in Section 3.4, avoiding dependence on predefined or hand-engineered image descriptors (Figure 8).

**Figure 8:**
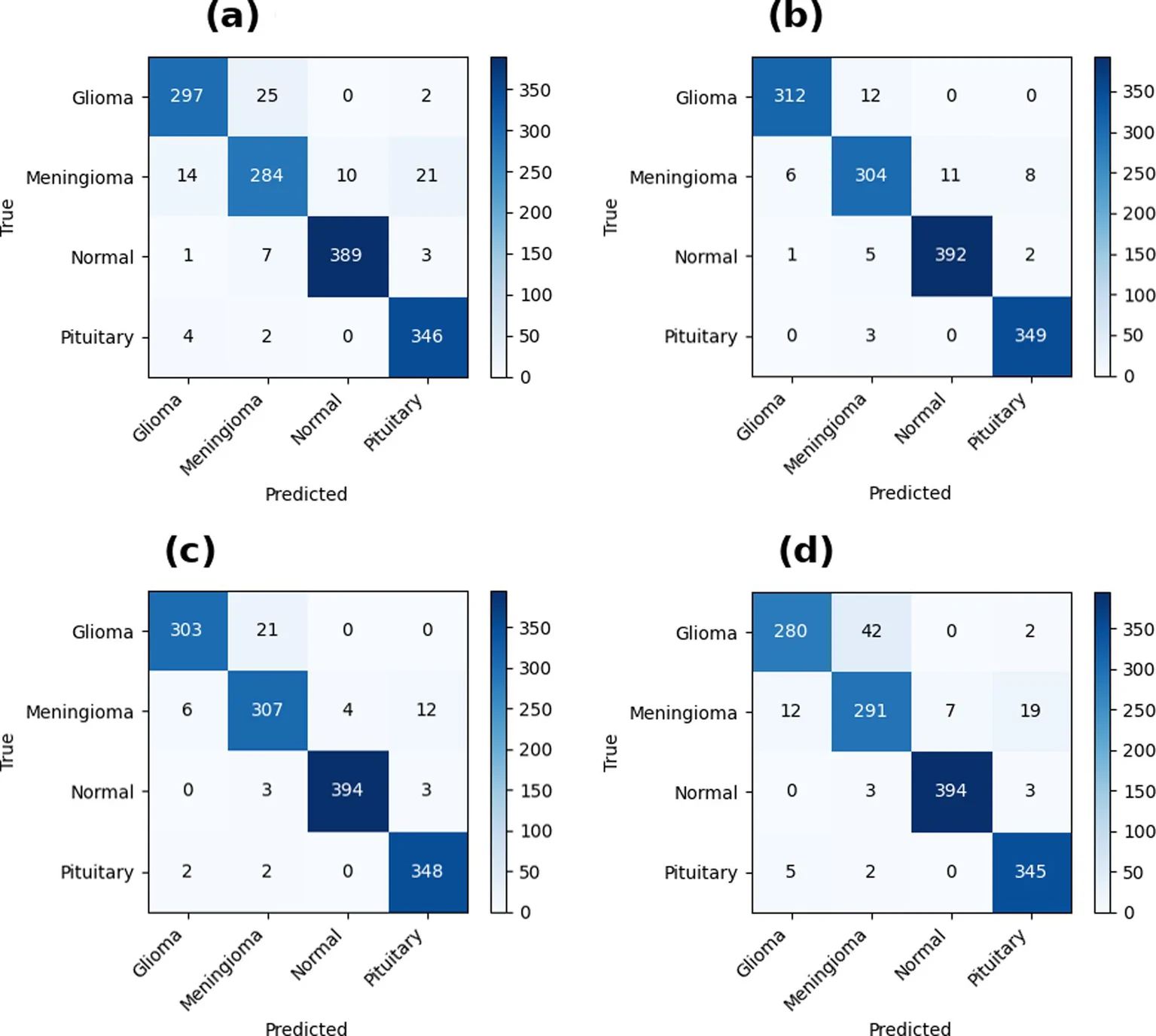
Confusion matrices of (a) CNN, (b) CNN-KNN, (c) CNN-SVM, and (d) CNN-RF on the hold-out test set.

For clarity, the architectural design choices summarized qualitatively above are consolidated into four concrete specifications. First, activation placement is applied consistently throughout the backbone: every convolutional operation, standard, depth-wise, or point-wise, is followed by batch normalization and then a ReLU nonlinearity, in that fixed order, across all four encoding stages; the only deviations from this pattern are the sigmoid gating function at the output of the SE excitation branch, which must produce a bounded [0, 1] channel-scaling weight rather than an unbounded activation, and the softmax (or classifier-head-specific) function at the network output. Second, parameter optimization follows a single fixed strategy rather than a searched one, selected empirically for training stability on the 256 × 256 × 3 input rather than by a grid or random search. The settings are specified in Section 4.2 and Table 6, along with the corresponding search spaces used for the three classical classifier heads. Third, regularization is provided entirely by architectural and scheduling choices rather than by explicit weight penalties: He-normal initialization throughout, batch normalization after every convolution, and a single dropout layer (rate 0.4) immediately before the classification head. L2 weight decay and image-level data augmentation were deliberately not used in this study, a design choice discussed further in Section 4.2 and noted as a direction for future work in Section 4.14. Fourth, the computational complexity of the resulting backbone, together with its CPU and GPU inference latency and throughput, is profiled in Section 4.5 and Table 7.

**Table 6:** Training configuration summary.

Model	Key hyperparameters	Search space	Selected configuration
CNN	Optimizer; learning rate; batch size; epochs; regularization	Adamax, LR = 1 × 10^−4^ (fixed strategy); batch = 16; ≤100 epochs; dropout = 0.4; He-normal init.	Fixed configuration—ReduceLROnPlateau (factor 0.5, patience 7) + EarlyStopping (patience 15, restore best weights)
KNN	Number of neighbors (k); distance metric; weighting	k ∈ {3, 5, 7, 9, 11}; Euclidean distance; uniform weighting	*k* = 5 (5-fold grid search on training partition)
SVM	Kernel; regularization C; kernel coefficient γ; feature scaling	RBF kernel; C ∈ {0.1, 1, 10, 100}; γ ∈ {‘scale’, 0.01, 0.001}; z-score standardization	C = 10, γ = ‘scale’; one-vs-rest decision function
RF	Number of trees; max depth; min samples/leaf; split criterion	n_estimators ∈ {100, 200, 300, 500}; max_depth ∈ {10, 20, None}; min_samples_leaf ∈ {1, 2, 4}; Gini impurity	n_estimators = 300, max_depth = 20, min_samples_leaf = 2; bootstrap sampling

**Table 7:** Computational profile of the shared CNN backbone (FP16 weights; batch-16 inference measured on CPU and on an NVIDIA A100 GPU).

Metric	Value
Computational cost	
Total parameters	107,076 (106,052 trainable + 1,024 non-trainable)
Floating-point operations per image	≈879.4 million FLOPs (≈440 million MACs)
Weight memory (FP16)	0.20 MB
Inference time (CPU)	
Latency per image	42.2 ms (median); 41.5 ms (minimum)
Throughput	23.7 images/s
Inference time (GPU)	
Latency per image	4.54 ms (median); 4.38 ms (minimum)
Throughput	220.12 images/s
Memory requirements	
Largest activation tensor	128.0 MB
Sum of all activations (upper bound)	2,320.2 MB (≈2.3 GB)
Process peak resident memory	≈1.36 GB
Peak GPU memory (batch 16)	4,932.6 MB (≈4.93 GB)

### 3.4 Classification

To identify the most effective decision layer for the 256-dimensional global-max-pooled embedding produced by the proposed backbone (Section 3.3), we evaluated the native softmax output of the network alongside three classical classifiers trained directly on the extracted features: KNN (Peterson, 2009), SVM (Cortés and Vapnik, 1995), and RF (Breiman, 2001). Decoupling representation learning, performed once by the shared CNN backbone, from decision boundary learning, performed independently by each classifier head, allows the relative merit of each classification strategy to be assessed without confounding differences in the underlying feature space.

The KNN classifier involves voting for the class label among the k nearest training examples based on a distance metric. The value of neighbors, k, was obtained from a cross-validated grid search using k ∈ {3, 5, 7, 9, 11}, which yielded an optimal k value of 5. The chosen dissimilarity metric is Euclidean (L2), because the 256-dimensional GlobalMaxPooling features have a well-clustered distribution such that the L2 metric reliably measures the semantic similarity of the tumor representation. Cosine similarity, which disregards magnitude information, and Manhattan distance, which is less affected by feature dimension correlation, performed slightly worse in the initial trials for the same validation split than the other metrics. Because KNN is a non-parametric model, it makes no assumptions regarding the distribution of the feature space, which works well in the case of highly variable tumor morphologies, as in this study.

The SVM classifier constructs a maximum-margin decision boundary by mapping the CNN embeddings into a higher-dimensional space via a Radial Basis Function (RBF) kernel and solving for the hyperplane that maximizes the separation between classes under a one-vs-rest multiclass scheme. Because SVM optimization is sensitive to feature scale, embeddings were standardized to zero mean and unit variance prior to fitting. The regularization parameter C and kernel coefficient γ were tuned jointly via a grid search. The RBF kernel was preferred over the linear kernel because the class boundaries observed in the embedding space, particularly between meningioma and normal tissue representations, were not linearly separable.

The RF classifier is an ensemble of decorrelated decision trees, each trained on a bootstrap-sampled subset of the training embeddings with a random subset of features considered at every split. Predictions are aggregated by majority vote across trees, which reduces the variance relative to a single decision tree and provides an implicit measure of feature importance. Unlike KNN and SVM, RF partitions the feature space using axis-aligned splits, which is a structurally different inductive bias whose suitability for this embedding is evaluated empirically alongside the other classifiers below.

These three classifiers, along with the softmax output layer of the CNN, were trained and validated using the same training-validation-testing procedure, ensuring that any difference in performance results mentioned below was due to the classifier only, and not because of any other reason, such as differences in data splitting or feature extraction. The performance of the classifier was analyzed using metrics such as Recall, Specificity, Precision, F1-Score, Accuracy, AUC, and Average Precision (AP) (Omeiza et al., 2019).

## 4 Results and discussion

This section offers a systematic, multi-layer evaluation of the suggested CNN backbone architecture combined with four other decision-making layers: CNN backbone with its default softmax head (CNN) and KNN, SVM, and RF classifiers, as discussed in Section 3.4. Two complementary resampling procedures were used to estimate performance: the hold-out protocol and stratified 5-fold cross-validation, both applied to all four configurations so that the two estimates could be compared directly against one another. This section also provides information about the complexity of the CNN backbone, the significance of the differences in the observed performance, ablation studies, feature visualization and interpretability analysis, and state-of-the-art comparison and implications from clinical and algorithmic perspectives.

### 4.1 Evaluation criteria

This subsection defines the quantitative measures used throughout the evaluation and specifies the two validation protocols under which those measures were computed, together with the reasoning for the adoption of each.

All models were assessed using seven complementary metrics— recall (sensitivity), Specificity, Precision, F1-score, Accuracy, AUC, and AP—computed per class and macro-averaged across the four diagnostic categories (normal, glioma, meningioma, pituitary) to avoid class-size bias, since the dataset, while reasonably balanced, is not perfectly uniform across classes. Two complementary validation regimes were adopted in this study. The hold-out protocol reserved 20% of the dataset (1,405 images) as an untouched, class-stratified test set and was used to report detailed class-wise diagnostic behavior, confusion matrices, and threshold-dependent curves (ROC, PR). The 5-fold cross-validation (5-FCV) protocol partitions the full dataset into five stratified folds, trains on four folds, validates on the held-out fold in rotation, and reports the mean performance across folds. Because every image is used exactly once for validation, 5-FCV yields a less optimistic and more broadly applicable estimate of generalization than a single hold-out split at the cost of five-fold higher training expense. In this study, the 5-FCV protocol was applied to all four classifier configurations rather than to the strongest one alone, so that the ranking established on the hold-out partition could be verified under resampling rather than being assumed to persist. The agreement between the two protocols is examined in Section 4.7. In addition, the statistical reliability of the accuracy improvement contributed by each classifier head was assessed using McNemar’s test and paired bootstrap resampling, rather than relying on point estimates alone.

### 4.2 Training configurations

This subsection specifies the computing environment, optimization settings, and hyperparameter search procedures used to train the CNN backbone and the three classical decision layers, and explains the reasoning behind each configuration choice rather than merely recording it.

The suggested CNN backbone architecture was trained in Google Colab on an NVIDIA A100 GPU, with TensorFlow 2.x and the Keras higher-level API through the Adamax optimizer, mini-batch of 16, and 100 epochs, employing the ReduceLROnPlateau and EarlyStopping methods to perform learning rate annealing and to terminate training when hitting a validation loss plateau, respectively. Initialization with He-normal, Batch Normalization, and dropout regularization before classification served as the primary sources of regularization. The three classifiers were trained on the pretrained 256-dimensional features obtained from this backbone model using only the training partition to perform hyperparameter optimization and to avoid test set leakage. Table 6 shows the final hyperparameter search spaces and selected configurations of the four models used in this study.

The configurations listed in Table 6 reflect specific and motivated design choices rather than arbitrary defaults. The Adamax optimizer was preferred over Adam and plain stochastic gradient descent because its per-parameter learning-rate normalization, based on the infinity norm of past gradients, was empirically more stable than Adam across preliminary trials on the MICA-enhanced input distribution, whose dynamic range differed from that of raw natural images. A mini-batch size of 16 was selected as a compromise between the gradient-estimate stability and the GPU memory required to hold 256 × 256 × 3 activations across the backbone’s four encoding stages. A batch size of 16 also matches the batch size used for the CPU and GPU inference throughput measurements reported in Table 7, maintaining the training and deployment configurations directly comparable. The dropout rate of 0.4, applied only immediately before the classification head, was chosen to be aggressive enough to discourage co-adaptation of the 256-dimensional pooled embedding without materially slowing convergence, consistent with its placement after all convolutional regularization (batch normalization) had already been applied. For the classical classifiers, the grid-search ranges in Table 6 were bounded by standard practice for embeddings of this dimensionality: k ∈ {3, 5, 7, 9, 11} for KNN brackets the point at which the bias-variance trade-off typically stabilizes for a few-hundred-dimensional feature space; C ∈ {0.1, 1, 10, 100} and γ ∈ {‘scale’, 0.01, 0.001} for SVM span three orders of magnitude of regularization strength and kernel width; and the RF ranges were selected to keep the ensemble large enough to reduce variance (up to 500 trees) while bounding tree depth to limit overfitting on a training partition of 5,618 images.

### 4.3 Hold-out results

This subsection reports the performance of the four configurations under the 80:20 hold-out protocol. The analysis proceeds in four stages: a quantitative examination of class-wise and macro-averaged metrics together with the corresponding confusion matrices; the accuracy and loss trajectories of the shared CNN backbone; a threshold-independent assessment of ranking quality using receiver operating characteristic curves; and a precision–recall analysis that responds more sensitively to behavior in the harder minority classes.

#### 4.3.1 Quantitative analysis

This subsection quantifies how each decision layer converts the shared feature representation into class assignments and identifies which diagnostic categories account for the residual error.

Table 8 reports the class-wise and macro-averaged performance of the CNN backbone under its native softmax head and each of the three classifier heads, evaluated on the 1,405-image holdout test set. In each of the four experiments, the normal and pituitary groups were identified with the greatest confidence, in line with their relatively unique MRI appearance (the presence of a distinct ventricular structure for normal and a distinct sellar region mass for pituitary adenomas). The meningioma group was the most easily mistaken in each experiment, presumably because of its variable radiological appearance and morphological similarity to surrounding structures rather than any specific weakness of the respective classifier. Replacing the CNN-based softmax classification head with a KNN or SVM decision-making head based on the same embedding improved the classification accuracy by approximately three percentage points (pp). This demonstrates the separation capability of the CNN features and that some of the errors of the softmax classification head stemmed from decision-making rather than representation-learning problems. In contrast, the RF head performed below the CNN’s own softmax head on the glioma and meningioma classes. Because RF partitions the embedding using axis-aligned splits, it is less able to exploit the smooth, continuous separability of the CNN feature manifold than the margin-based (SVM) or distance-based (KNN) alternatives, a pattern that recurs across the ROC, PR, and radar analyses.

**Table 8:** Class-wise performance under the hold-out evaluation protocol.

Method	Tumor type	Performance metrics (%)				
		Recall	Specificity	Precision	F1-score	Accuracy
CNN	Glioma	91.67	98.24	93.99	92.82	96.72
	Meningioma	86.32	96.84	89.31	87.81	94.38
	Normal	97.25	99.00	97.49	97.37	98.51
	Pituitary	98.30	97.53	93.01	95.60	97.72
	Average	93.39	97.90	93.45	93.40	93.67
CNN-KNN	Glioma	96.30	99.35	97.81	97.05	98.65
	Meningioma	92.40	98.14	93.83	93.09	96.80
	Normal	98.00	98.91	97.27	97.63	98.65
	Pituitary	99.15	99.05	97.21	98.17	99.07
	Average	96.46	98.86	96.53	96.49	96.58
CNN-SVM	Glioma	93.52	99.26	97.43	95.46	97.94
	Meningioma	93.31	97.58	92.19	92.73	96.58
	Normal	98.50	99.60	98.99	98.74	99.29
	Pituitary	98.86	98.58	95.87	97.37	98.65
	Average	96.05	98.76	96.12	96.08	96.23
CNN-RF	Glioma	86.42	98.43	94.28	90.20	95.66
	Meningioma	88.45	95.63	86.09	87.24	93.95
	Normal	98.50	99.30	98.25	98.37	99.07
	Pituitary	98.01	97.72	93.50	95.72	97.79
	Average	92.85	97.77	93.03	92.88	93.24

#### 4.3.2 Accuracy and loss curves

This subsection inspects the optimization behavior of the shared CNN backbone to establish that the representation on which all four decision layers operate was learned stably rather than memorized.

Figure 9 shows the training and validation accuracies and cross-entropy loss of the CNN backbone across 100 epochs. Because the classifier heads operate on the frozen embedding produced by this backbone, these curves characterize the shared representation learned prior to all four configurations in Table 8, rather than any one classifier individually. The validation accuracy converged within approximately 20 epochs, after which further training refined the decision boundaries rather than the gross feature quality. The persistently small training–validation gap in both curves, sustained by batch normalization, a pre-classification dropout layer, and the learning-rate/early stopping schedule, indicates that the backbone generalizes rather than memorizes.

**Figure 9:**
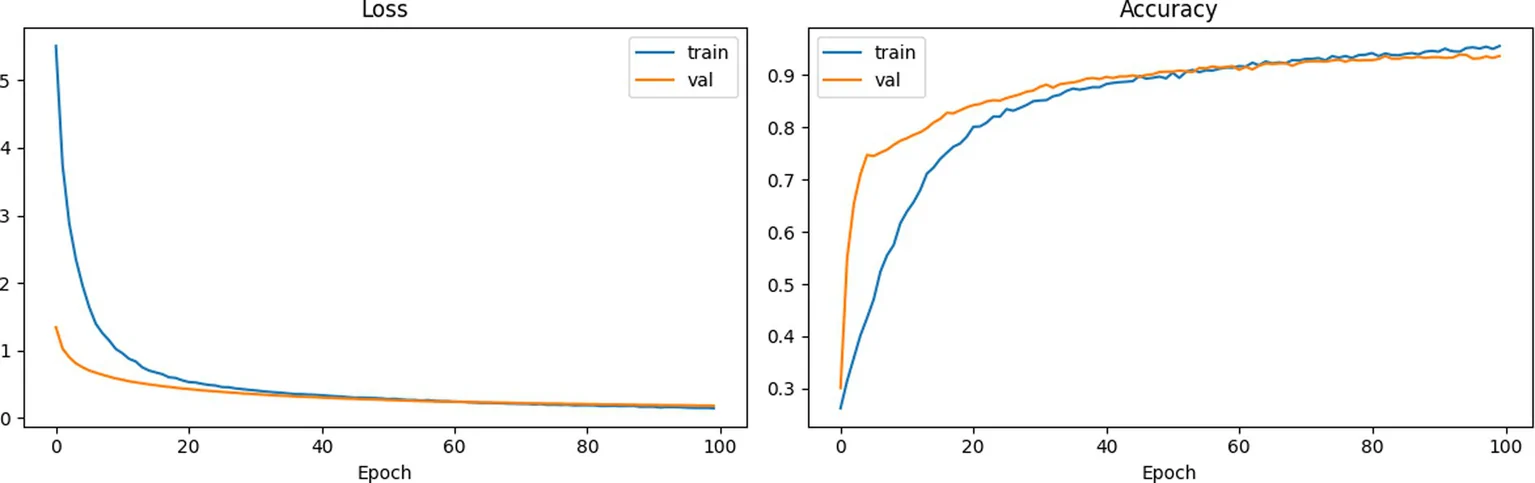
Training and validation accuracy (right) and loss (left) curves of the CNN backbone.

#### 4.3.3 Receiver operating characteristic (ROC) curves

This subsection assesses the threshold-independent discriminative capacity of the four configurations, thereby separating the ability to rank cases correctly from the ability to label them correctly at a fixed operating point.

Figure 10 presents the class-wise ROC curves for all four configurations, and Figure 11 summarizes the resulting macro-averaged AUC values. All four configurations achieved a macro-AUC above 99%, confirming that each classifier head, including the plain softmax layer, ranked the positive and negative cases correctly for most image pairs. Therefore, the practically meaningful differences between classifiers lie in the operating point behavior captured in Table 8, rather than the ranking ability. CNN-SVM attained the highest macro-AUC (99.56%), consistent with the margin-maximizing objective of SVM producing better-separated decision scores than the vote-based KNN output or the CNN’s native softmax probabilities, while CNN-RF’s AUC (99.16%) was practically indistinguishable from the base CNN’s (99.17%), reinforcing the pattern that the RF head adds little discriminative value beyond the backbone’s softmax layer.

**Figure 10:**
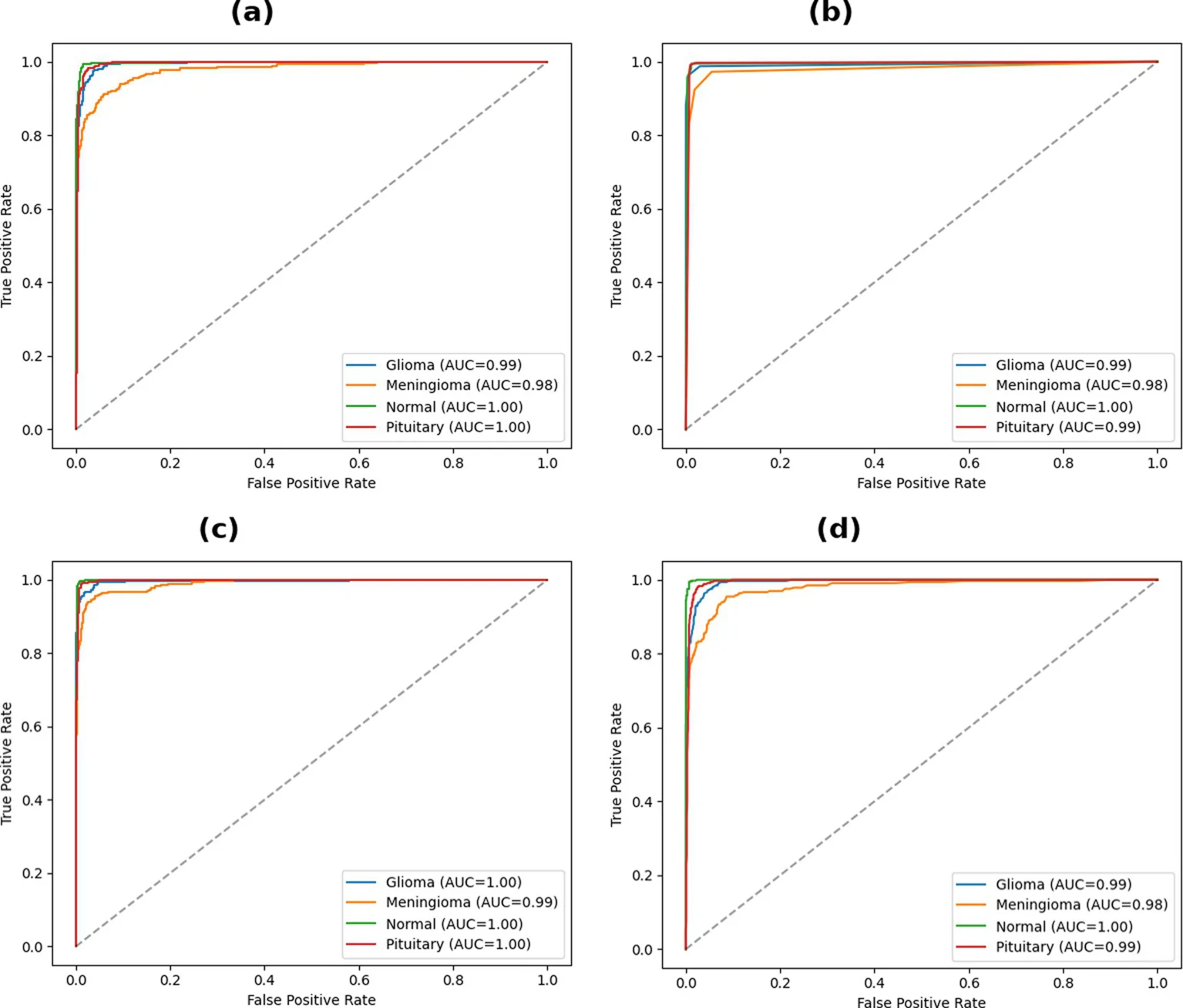
ROC curves of (a) CNN, (b) CNN-KNN, (c) CNN-SVM, and (d) CNN-RF on the hold-out test set.

**Figure 11:**
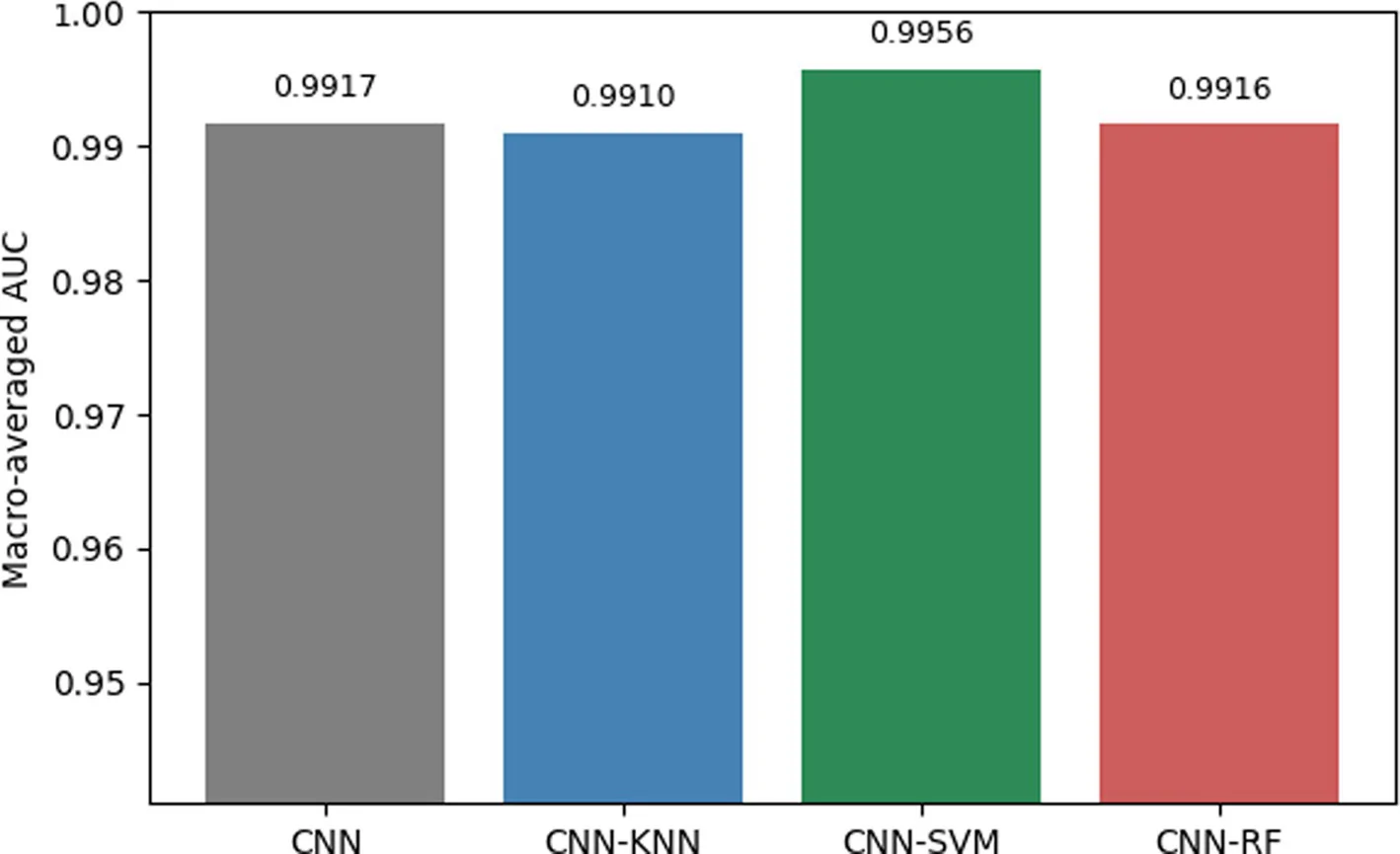
Macro-averaged AUC comparison across classifier heads.

#### 4.3.4 Precision–recall (PR) curves

This subsection complements the ROC analysis with precision–recall curves, which are unaffected by the size of the true-negative population and, therefore, respond more sensitively to the precision attainable at high recall on the minority diagnostic classes.

Figure 12 presents the class-wise precision–recall curves, and Figure 13 summarizes the macro-averaged AP. Because the class distribution was only mildly imbalanced, the AP ranking broadly mirrored the AUC ranking, with CNN-SVM again being the strongest (AP = 98.81%) and CNN-KNN marginally below the base CNN (AP = 97.48% versus 97.77%), despite CNN-KNN’s superior accuracy in Table 8. This divergence is informative: it shows that KNN’s majority-vote decision rule improves hard-label classification at the operating threshold used to build the confusion matrix, without necessarily improving the ranking of predictions across all thresholds—precisely the distinction that AUC/AP and Accuracy/F1 are each designed to capture, and one that matters for downstream use cases such as probability-based risk stratification rather than single-label diagnosis.

**Figure 12:**
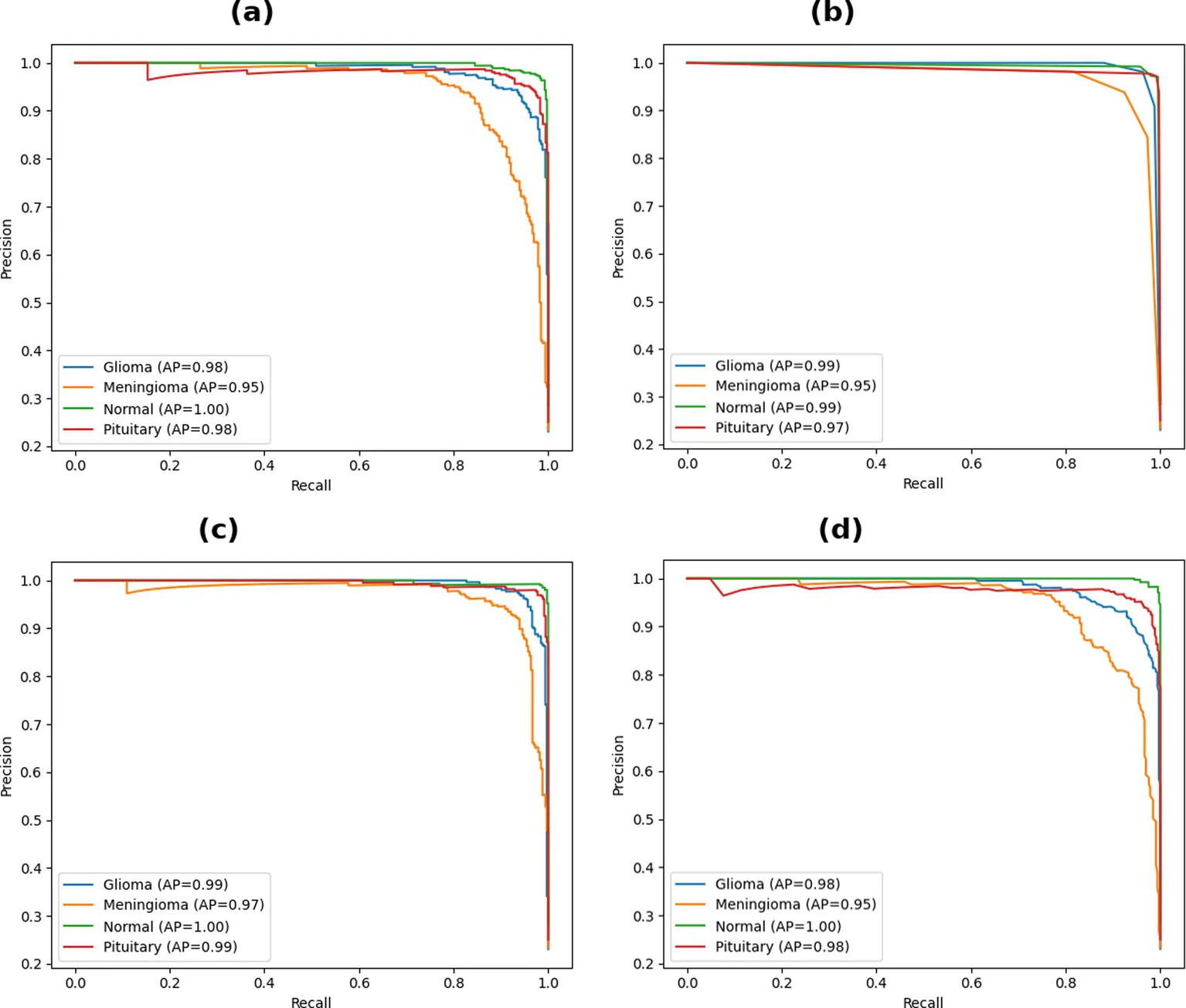
Precision–recall curves of (a) CNN, (b) CNN-KNN, (c) CNN-SVM, and **(d)** CNN-RF on the hold-out test set.

**Figure 13:**
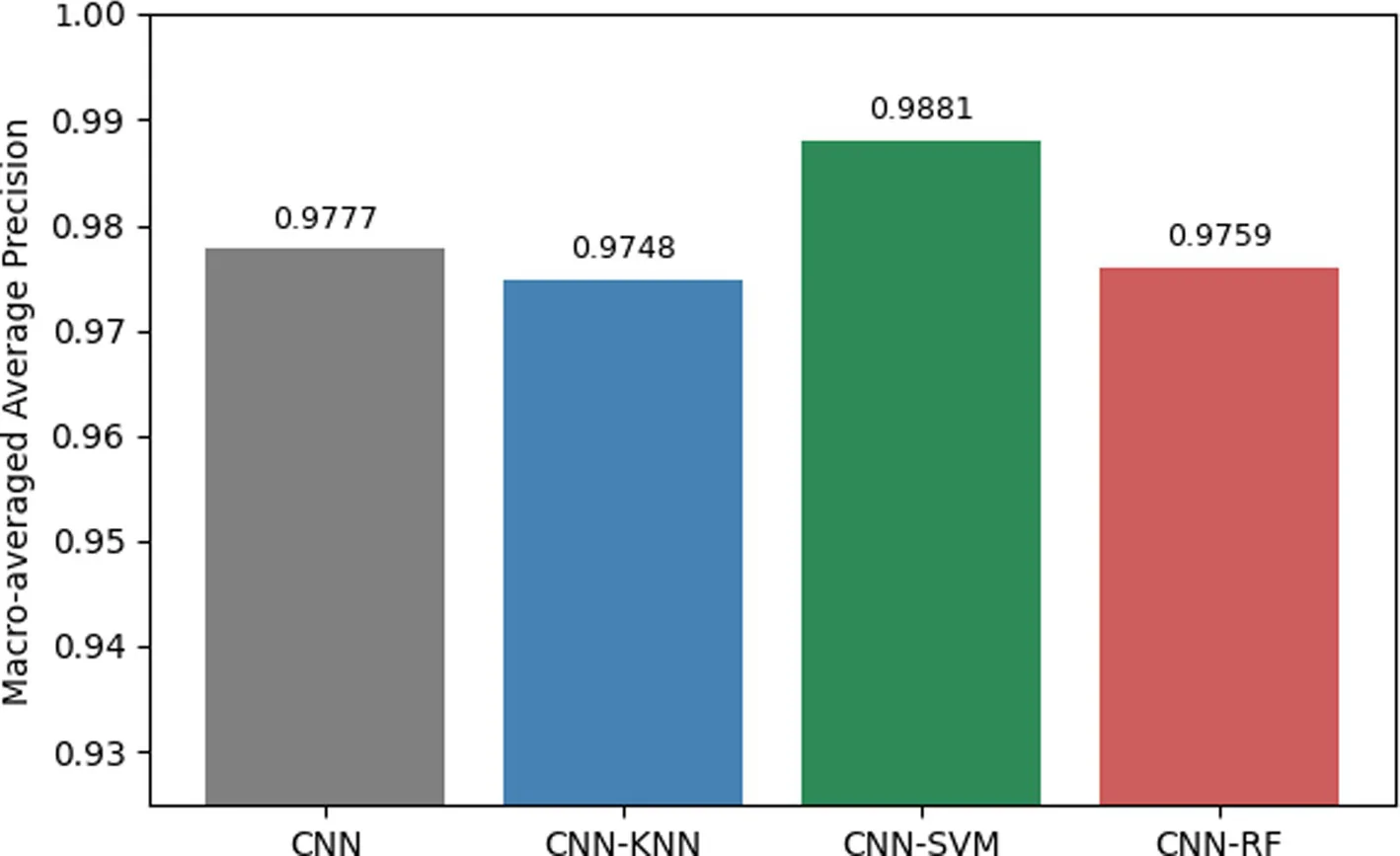
Macro-averaged average precision comparison across classifier heads.

Finally, Figure 14 consolidates the five macro-averaged metrics—Accuracy, Recall, Specificity, Precision, and F1-score—into a single radar profile for all four configurations. CNN-KNN and CNN-SVM produced almost coincident outermost profiles across all five axes, whereas CNN-RF and the base CNN traced visibly smaller, largely overlapping profiles. The consistent ordering across every axis, rather than a trade-off in which one configuration wins on some metrics and loses on others, confirms that representation quality is shared by all four configurations, and the choice of decision layer determines the operating performance realized from that shared representation.

**Figure 14:**
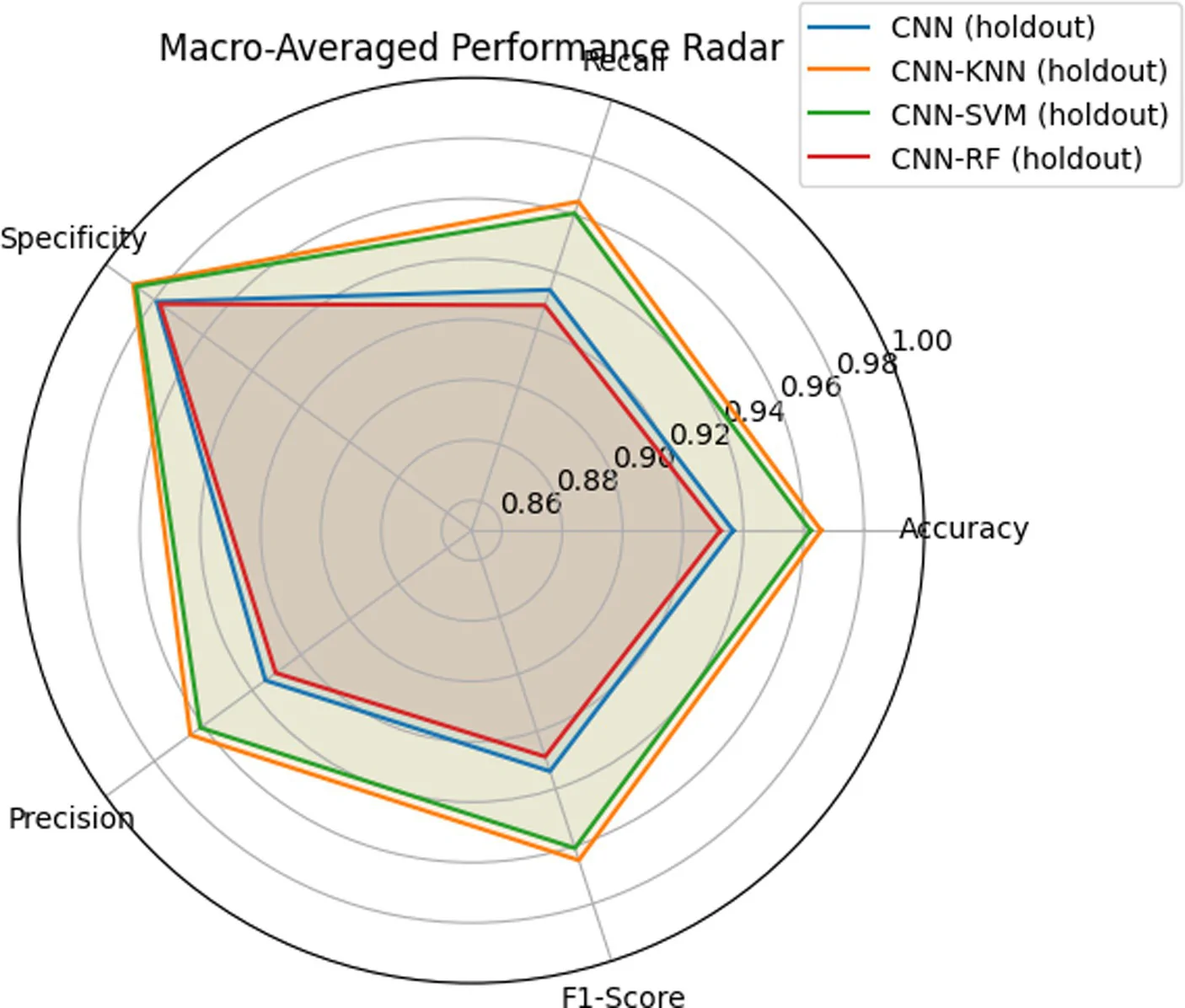
Radar comparison of macro-averaged performance metrics across classifier heads.

Taken together, the hold-out results identify a KNN or SVM decision layer as the preferred choice for this feature embedding, with KNN maximizing hard-label accuracy and SVM maximizing probability-ranking quality, whereas the RF head offers no consistent advantage over the CNN’s own softmax classifier.

### 4.4 5-fold cross-validation

After characterizing the four configurations on a single stratified partition, this subsection establishes whether the characterization survives repeated resampling of the training and evaluation data. The analysis deliberately mirrors the four-stage structure adopted in Section 4.3: a quantitative analysis of fold-wise and aggregated metrics together with the corresponding confusion matrices, the accuracy and loss trajectories of the CNN backbone, a threshold-independent assessment based on receiver operating characteristic curves, and a precision–recall analysis—so that the two validation protocols may be placed directly alongside one another in Section 4.7.

The complete corpus of 7,023 images was partitioned into five class-stratified folds under a fixed random seed, producing training partitions of 5,618–5,619 images and evaluation partitions of 1,404–1,405 images, with the class proportions of the parent distribution preserved in each fold (approximately 324 glioma, 329 meningioma, 400 normal, and 351 pituitary images per evaluation fold). For each fold, a CNN backbone was instantiated afresh and trained from random initialization, after which the three classical decision layers were re-fitted, including an independent hyperparameter search restricted to that fold’s training partition, on the frozen 256-dimensional embeddings extracted from the corresponding backbone. Because every image is used for evaluation exactly once and no image ever contributes to both the training and evaluation of the same fold, the dispersion reported below reflects the variability in the learned representation and fitted decision boundary. Therefore, it constitutes a considerably stricter test of stability than the repeated refitting of a single fixed embedding.

#### 4.4.1 Quantitative analysis

This subsection reports the fold-wise and aggregated numerical performance of the four configurations and then decomposes those aggregates into class-wise behavior recovered by pooling the five-fold-level confusion matrices into a single corpus-wide matrix. Table 9 presents the per-fold values for all six macro-averaged metrics, along with the mean and standard deviation across the folds.

**Table 9:** Fold-wise and aggregated performance of the four configurations under stratified 5-fold cross-validation.

Method	Fold	Performance measures (%)					
		Sensitivity	Specificity	Precision	F1-score	Accuracy	AUC
CNN	1	93.51	97.94	93.50	93.49	93.74	99.42
	2	94.40	98.24	94.49	94.43	94.66	99.45
	3	93.04	97.80	93.18	93.07	93.38	99.21
	4	94.19	98.18	94.34	94.23	94.52	99.20
	5	93.16	97.83	93.23	93.16	93.45	99.24
	Mean ± SD	93.66 ± 0.61	98.00 ± 0.20	93.75 ± 0.62	93.68 ± 0.62	93.95 ± 0.60	99.30 ± 0.12
CNN-KNN	1	96.49	98.90	96.52	96.49	96.65	99.41
	2	97.12	99.08	97.17	97.14	97.22	99.42
	3	95.07	98.44	95.19	95.10	95.30	99.03
	4	95.70	98.66	95.78	95.71	95.94	98.94
	5	95.25	98.47	95.30	95.22	95.37	98.92
	Mean ± SD	95.92 ± 0.86	98.71 ± 0.28	95.99 ± 0.84	95.93 ± 0.87	96.10 ± 0.83	99.14 ± 0.25
CNN-SVM	1	96.31	98.83	96.29	96.30	96.44	99.67
	2	95.27	98.53	95.34	95.28	95.52	99.60
	3	94.71	98.33	94.76	94.72	94.95	99.64
	4	95.75	98.68	95.88	95.81	96.01	99.40
	5	95.26	98.50	95.27	95.26	95.44	99.67
	Mean ± SD	95.46 ± 0.60	98.57 ± 0.19	95.51 ± 0.59	95.47 ± 0.60	95.67 ± 0.57	99.59 ± 0.11
CNN-RF	1	94.24	98.20	94.26	94.24	94.52	99.51
	2	94.32	98.22	94.45	94.34	94.59	99.48
	3	92.53	97.65	92.62	92.55	92.88	99.34
	4	93.96	98.12	94.13	94.01	94.30	99.22
	5	93.37	97.90	93.42	93.36	93.66	99.44
	Mean ± SD	93.68 ± 0.75	98.02 ± 0.24	93.78 ± 0.75	93.70 ± 0.75	93.99 ± 0.72	99.40 ± 0.12

Three observations can be made from Table 9. First, the ordering of the four configurations established under the hold-out protocol was reproduced without exception across the resampled folds: CNN-KNN attained the highest mean accuracy (96.10%), followed by CNN-SVM (95.67%), CNN-RF (93.99%), and the native softmax head (93.95%) occupying the lower tier. Second, the fold-to-fold dispersion is modest in absolute terms, with standard deviations of 0.57 to 0.83 accuracy points across the four configurations, indicating that neither the learned representation nor the fitted decision boundary is unduly sensitive to which particular fifth of the corpus is withheld, and that the accuracy reported for any single split is a reasonable proxy for the expected performance of the pipeline. Third, and most consequentially for the interpretation of the framework, the 0.04-point separation between CNN-RF and the CNN softmax head is more than an order of magnitude smaller than the standard deviation of either estimate; under cross-validation, the RF head is not merely statistically indistinguishable from the backbone’s own classifier but marginally ahead of it, a sign reversal relative to the hold-out split that independently corroborates the null result returned by McNemar’s test in Section 4.6.

A more instructive pattern emerges when accuracy and AUC are considered together rather than in isolation. CNN-SVM recorded the highest mean AUC (99.59 ± 0.11%) while ranking second in accuracy, whereas CNN-KNN recorded the highest accuracy but the lowest mean AUC of the four configurations (99.14 ± 0.25%), and notably, the widest dispersion on that metric. This dissociation replicates the same phenomenon observed in the hold-out results under a substantially more demanding evaluation regime, and its algorithmic origin is transparent. The pseudo-probabilities emitted by a KNN head are the fractions of retrieved neighbors belonging to each class and are therefore quantized to a small number of discrete levels. With the grid search selecting *k* = 3 in four of the five folds, only four distinct scores were attainable per class. This resolution is sufficient to assign the correct hard label but is far too coarse to order cases finely across the continuum of decision thresholds that an AUC integrates over. In contrast, the margin-based SVM produces a continuous decision function whose magnitude varies smoothly with the distance of an embedding from the separating hyperplane, yielding a strictly better ranking. The practical implication is that the choice of decision layer should follow the intended clinical use rather than a single headline metric: a distance-based head where a categorical diagnosis is required, and a margin-based head wherever the output will be thresholded, calibrated, or used to prioritize a radiological reading queue. To expose the diagnostic categories responsible for the residual error, the five-fold confusion matrices of each configuration were pooled into a single corpus-wide matrix spanning all 7,023 images. Table 10 reports the resulting class-wise performance, which is directly comparable in structure to the hold-out results in Table 8.

**Table 10:** Pooled class-wise performance under the 5-fold cross-validation protocol (all 7,023 images, each evaluated exactly once).

Method	Tumor type	Performance measures (%)				
		Recall	Specificity	Precision	F1-score	Accuracy
CNN	Glioma	92.04	98.11	93.60	92.81	96.71
	Meningioma	87.42	97.17	90.44	88.90	94.89
	Normal	98.25	98.96	97.42	97.83	98.76
	Pituitary	96.93	97.74	93.47	95.17	97.54
	Average	93.66	98.00	93.73	93.68	93.95
CNN-KNN	Glioma	95.68	98.59	95.33	95.50	97.92
	Meningioma	90.70	98.48	94.79	92.70	96.65
	Normal	98.40	99.26	98.15	98.28	99.02
	Pituitary	98.92	98.50	95.65	97.26	98.60
	Average	95.92	98.71	95.98	95.93	96.10
CNN-SVM	Glioma	93.83	98.48	94.88	94.35	97.41
	Meningioma	91.85	97.62	92.19	92.02	96.27
	Normal	98.95	99.42	98.56	98.75	99.29
	Pituitary	97.21	98.77	96.33	96.77	98.38
	Average	95.46	98.57	95.49	95.47	95.67
CNN-RF	Glioma	90.44	98.19	93.73	92.06	96.40
	Meningioma	89.24	96.80	89.51	89.38	95.03
	Normal	98.70	99.36	98.40	98.55	99.17
	Pituitary	96.36	97.72	93.38	94.85	97.38
	Average	93.68	98.02	93.76	93.71	93.99

The class-wise decomposition in Table 10 reproduces the diagnostic hierarchy observed under the holdout protocol and sharpens its clinical interpretation. Normal studies were recognized most reliably by every configuration (recall 98.25–98.95%), which is consistent with the comparatively unambiguous appearance of preserved ventricular anatomy and midline symmetry in the absence of a mass lesion. Meningioma remains the weakest class throughout, with recall ranging from 87.42% for the softmax head to 91.85% for CNN-SVM, and it is the only category for which no configuration exceeds 92%. Critically, this deficit persists across five independent partitions of the data and four structurally dissimilar decision rules, which suggests that it originates in the radiological signal itself rather than in any particular classifier. Meningiomas are extra-axial, broad-based dural lesions whose intensity profiles on unenhanced sequences frequently approximate those of adjacent cortex and dural venous structures. The resulting overlap in the embedding space cannot be resolved by a better decision boundary.

The inspection of the off-diagonal mass identifies a single dominant error axis. Pooled across folds, bidirectional glioma–meningioma confusion accounted for 45% of all CNN-KNN errors (123 of 274) and between 43 and 50% of the errors of the remaining configurations, whereas the second largest channel, meningioma misassigned to pituitary, contributed a further 46 to 82 cases depending on the head. Both channels are radiologically interpretable: gliomas and meningiomas may both present as hyperintense supratentorial masses when the extra-axial plane of origin is not resolvable in a single two-dimensional slice, and a parasellar or sphenoid-wing meningioma may occupy nearly the same anatomical territory as a macroadenoma. Both observations point to the same remedy, namely the incorporation of volumetric context or contrast-enhanced sequences that would render the dural attachment and plane of origin explicit, rather than further refinement of the decision layer.

Recasting the pooled matrices into the binary screening question that governs triage—whether a study contains a tumor at all—yields a complementary and clinically more actionable ranking. Of the 5,023 tumor-bearing studies in the corpus, the number erroneously assigned to the normal category was 52 for the softmax head (tumor detection sensitivity, 98.96%), 37 for CNN-KNN (99.26%), 32 for CNN-RF (99.36%), and only 29 for CNN-SVM (99.42%). In contrast, the number of normal studies over-called as tumors was 35, 32, 26, and 21, respectively. CNN-SVM, therefore, commits the fewest errors of both clinically consequential types despite ranking second on overall accuracy because the errors it makes are concentrated in the glioma–meningioma subtype boundary rather than at the tumor–normal boundary. Because a missed tumor delays definitive management, whereas a confused tumor subtype is ordinarily corrected at the subsequent histopathological or contrast-enhanced review, this redistribution of error is materially more favorable than the aggregate accuracy figures alone would suggest, and it substantiates the recommendation of a margin-based decision layer for screening-oriented deployment. Notably, almost the entire tumor-to-normal error mass across every configuration was contributed by meningioma, reinforcing the conclusion that this class governs the clinically limiting behavior of the framework.

Figures 15–18 present the fold-level confusion matrices from which Table 10 was aggregated, permitting the stability of these error patterns to be inspected fold-by-fold.

**Figure 15:**
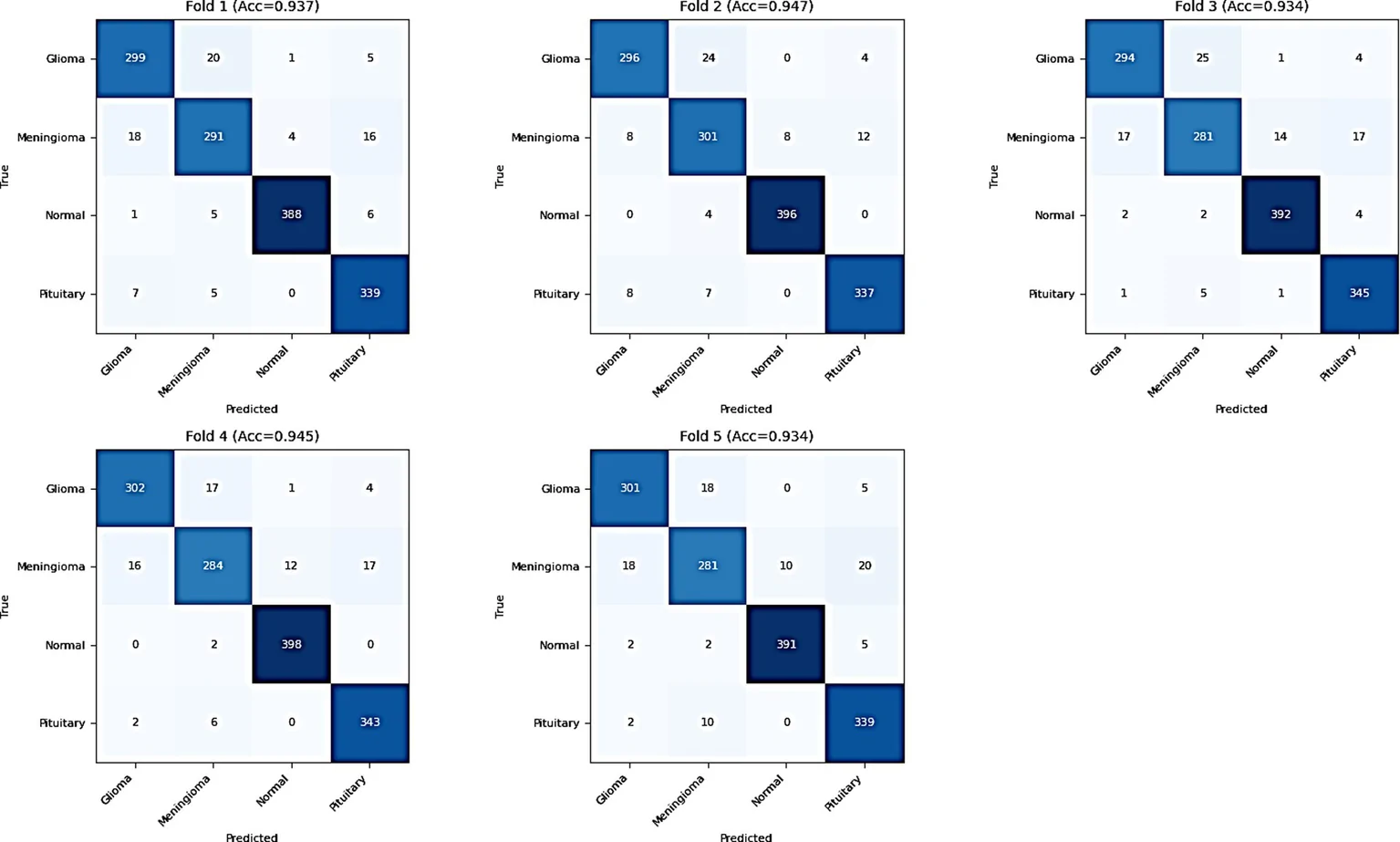
Fold-wise confusion matrices of the CNN backbone with its native softmax head under 5-fold cross-validation.

**Figure 16:**
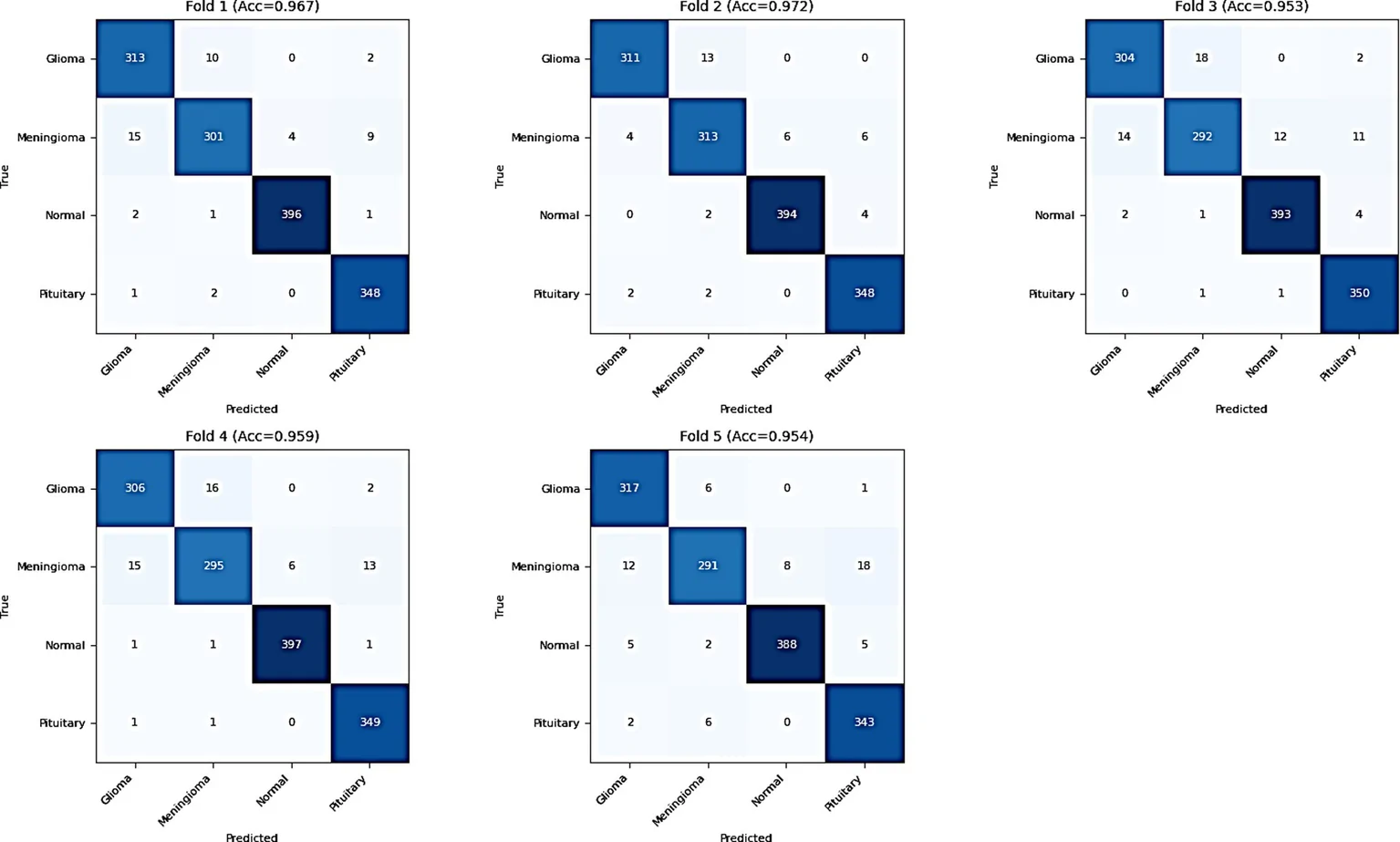
Fold-wise confusion matrices of CNN-KNN under 5-fold cross-validation.

**Figure 17:**
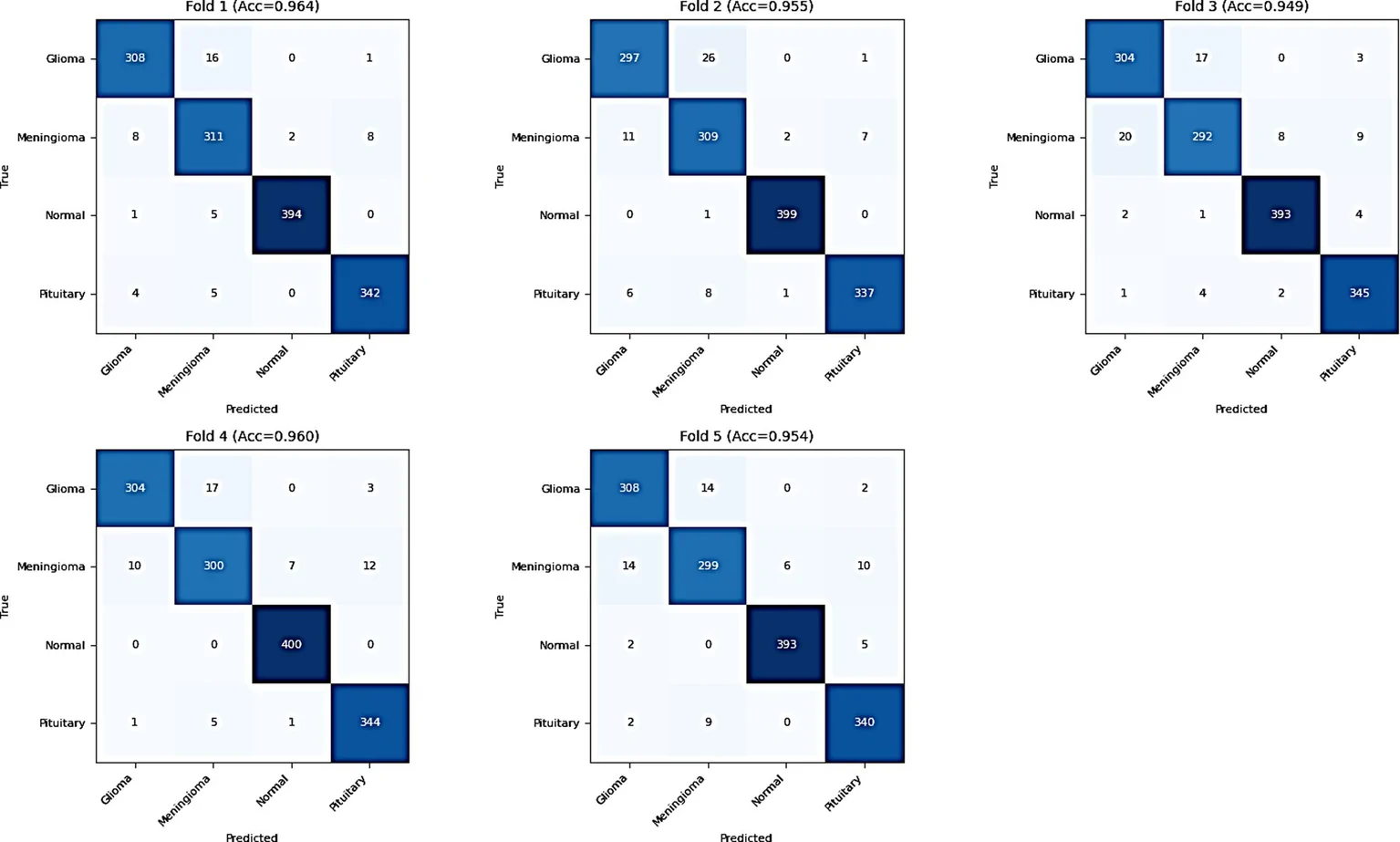
Fold-wise confusion matrices of CNN-SVM under 5-fold cross-validation.

**Figure 18:**
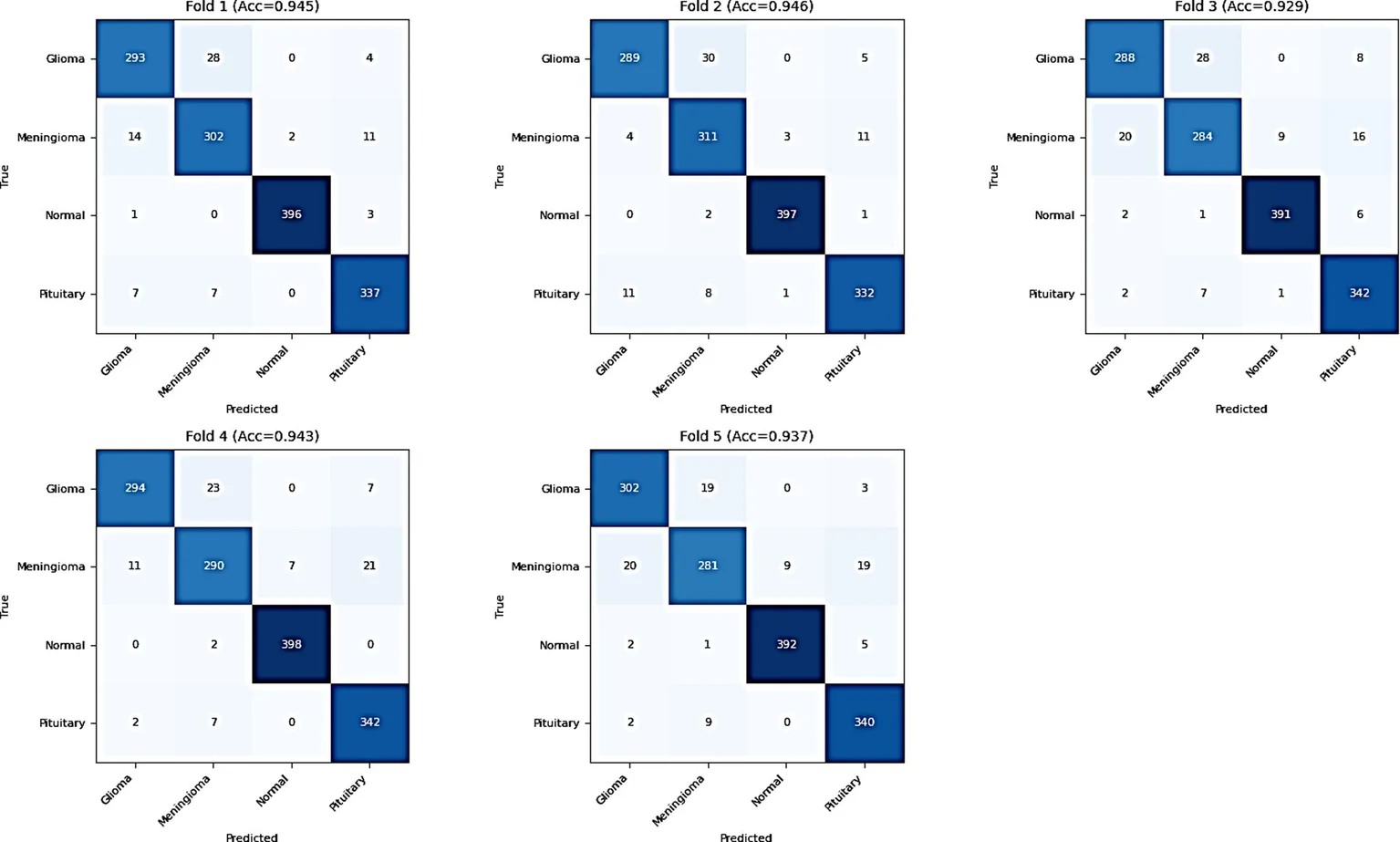
Fold-wise confusion matrices of CNN-RF under 5-fold cross-validation.

Across all four configurations, the qualitative structure of the matrices was preserved from fold to fold: the normal row was nearly diagonal in every panel, the glioma–meningioma off-diagonal cells were consistently the most heavily populated, and no fold exhibited a systematic collapse of any class. The absence of an outlier fold is itself an informative negative result, since a single anomalous partition would have signalled either an unstable optimization trajectory or the presence of a concentrated subpopulation of atypical studies within the corpus.

#### 4.4.2 Accuracy and loss curves

This subsection examines the optimization behavior of the CNN backbone across the five folds to establish that the representation on which all four decision layers operate was acquired stably and reproducibly, rather than through a single fortunate initialization. Because the classical heads are fitted in closed form on frozen embeddings and are not trained epoch by epoch, these trajectories characterize the shared feature extractor common to every configuration listed in Table 9. Figures 19, 20 present the training and validation accuracies and the categorical cross-entropy loss, respectively, for each of the five folds over the full 100-epoch budget.

**Figure 19:**
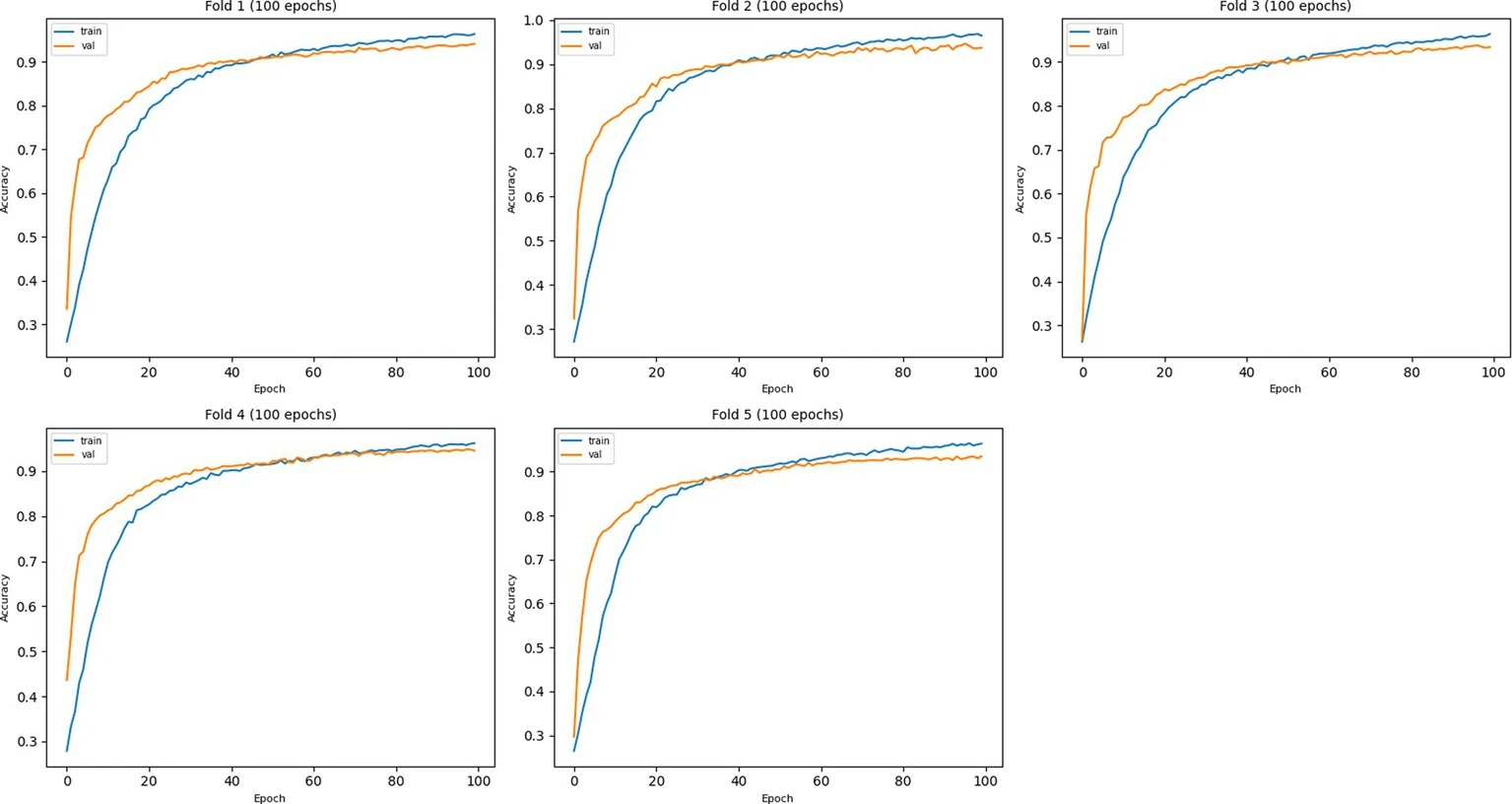
Fold-wise training and validation accuracy curves of the CNN backbone under 5-fold cross-validation.

**Figure 20:**
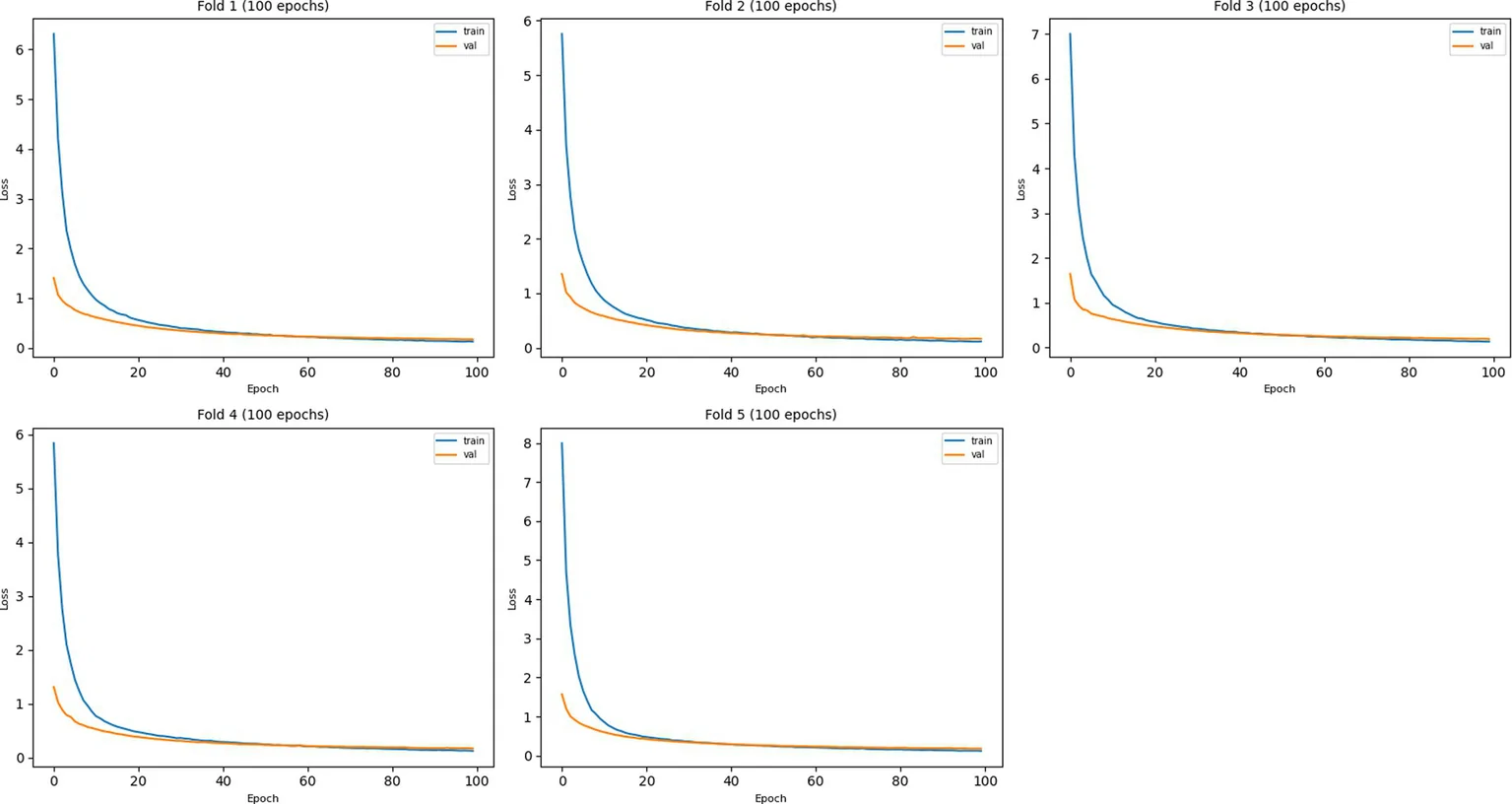
Fold-wise training and validation loss curves of the CNN backbone under 5-fold cross-validation.

The optimization trends for the five folds followed an extremely similar pattern. For each of the folds, the trend started close to the 25% probability of a balanced four-class problem, rapidly ascended over the initial 20 epochs, and then became very slow from approximately epoch 40 onwards until it converged at a final validation accuracy level of 93 to 95% with a validation loss of less than 0.20. This consistent spread between the five optimization curves is the key point of this subsection: the variance found in the final performance figures in Table 9 results from the random assignment of studies to different evaluation folds, not from instability during optimization.

There are two aspects of these curves that are worth mentioning. First, the validation curve is always above the training curve for the early epochs across all five folds, and both intersect only at approximately epoch 50. This reversal of roles is a clear indicator of the presence of regularization; indeed, the dropout function and batch normalization statistics are active during the training stage but inactive during inference. Therefore, the training performance of the network was assessed under an artificially hampered stochastic setting, whereas the validation performance was assessed under a full deterministic setting. The gradual closure of the gap indicates that the network progressively reduced its dependence on the co-adaptation that dropout was designed to suppress. Second, and even more significantly for the validity of the reported results, no fold displays a discrepancy between the training and validation losses that is indicative of memorization, with the two loss functions remaining close until epoch 100, with the residual gap never exceeding a fraction of a nat. Because no image augmentation was performed and no weight decay was used explicitly, the only possible explanation for this phenomenon is the inherent architectural regularization from batch normalization, pre-classification dropout, and the small number of parameters of the depth-wise separable backbone architecture. In the absence of the third validation set, the evaluation set was also used for the purpose of learning rate annealing and early stopping; therefore, it is better to understand these loss functions as an indication of convergence. The consequences of this setup for the reported estimates are discussed in Section 4.7 of this paper.

#### 4.4.3 Receiver operating characteristic (ROC) curves

This subsection evaluates the threshold-independent discriminative capacity of the four configurations under cross-validation, thereby separating the ability of each model to rank studies correctly from its ability to label them correctly at one fixed operating point. Figures 21–24 present the one-versus-rest ROC curves for each diagnostic class in each fold, annotated with the per-class and macro-averaged areas under the curve.

**Figure 21:**
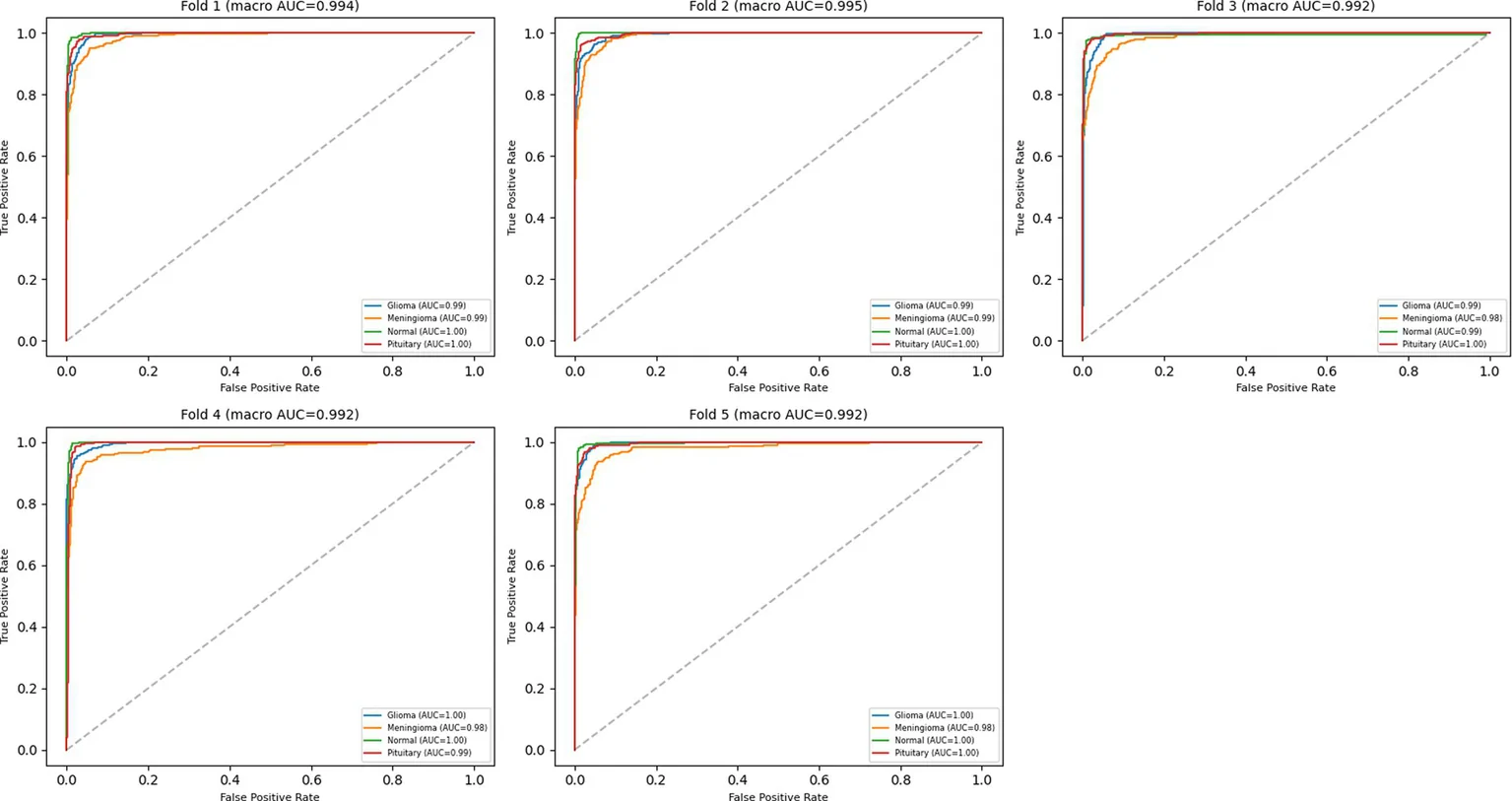
Fold-wise ROC curves of the CNN backbone with its native softmax head under 5-fold cross-validation.

**Figure 22:**
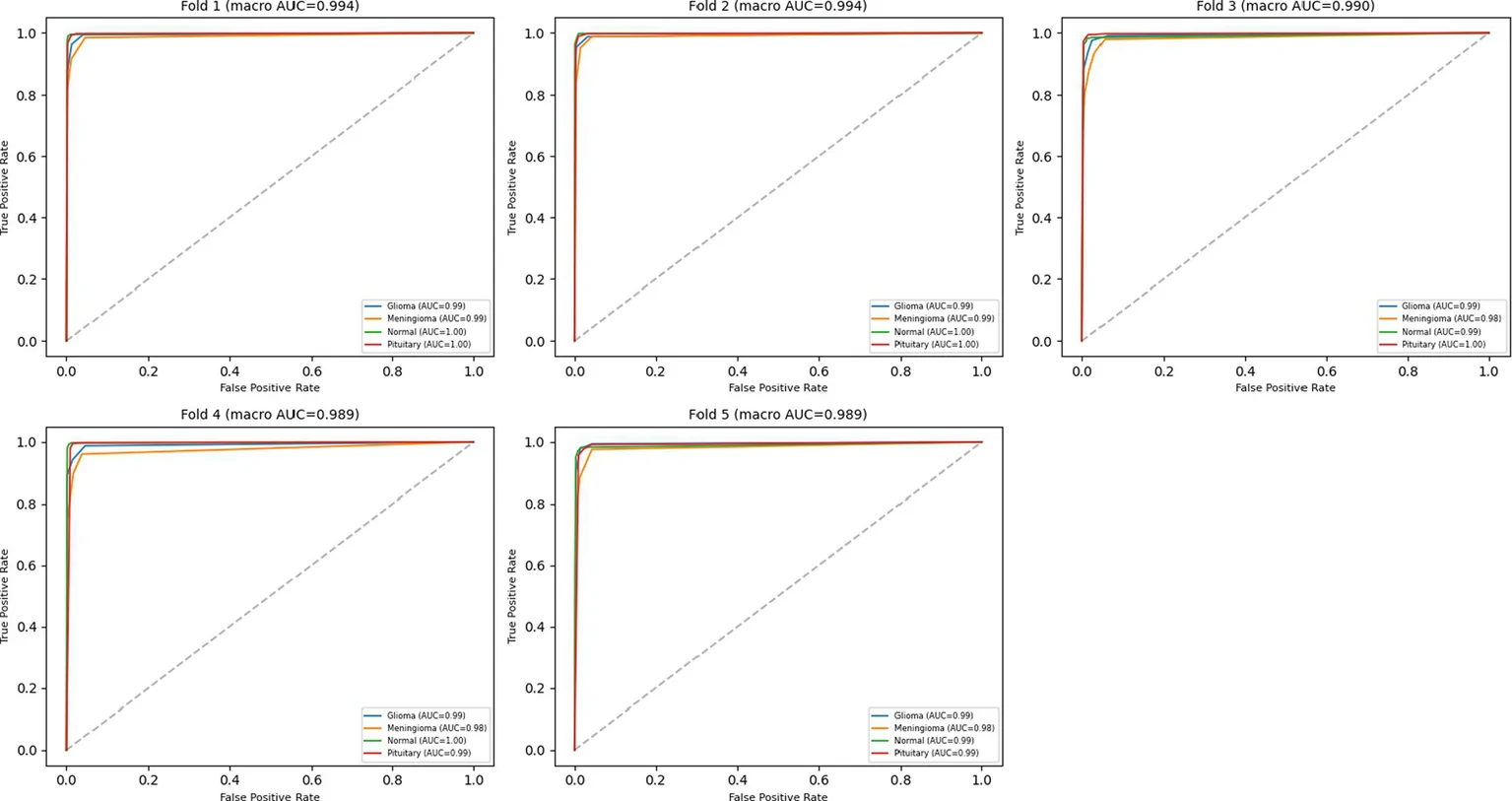
Fold-wise ROC curves of CNN-KNN under 5-fold cross-validation.

**Figure 23:**
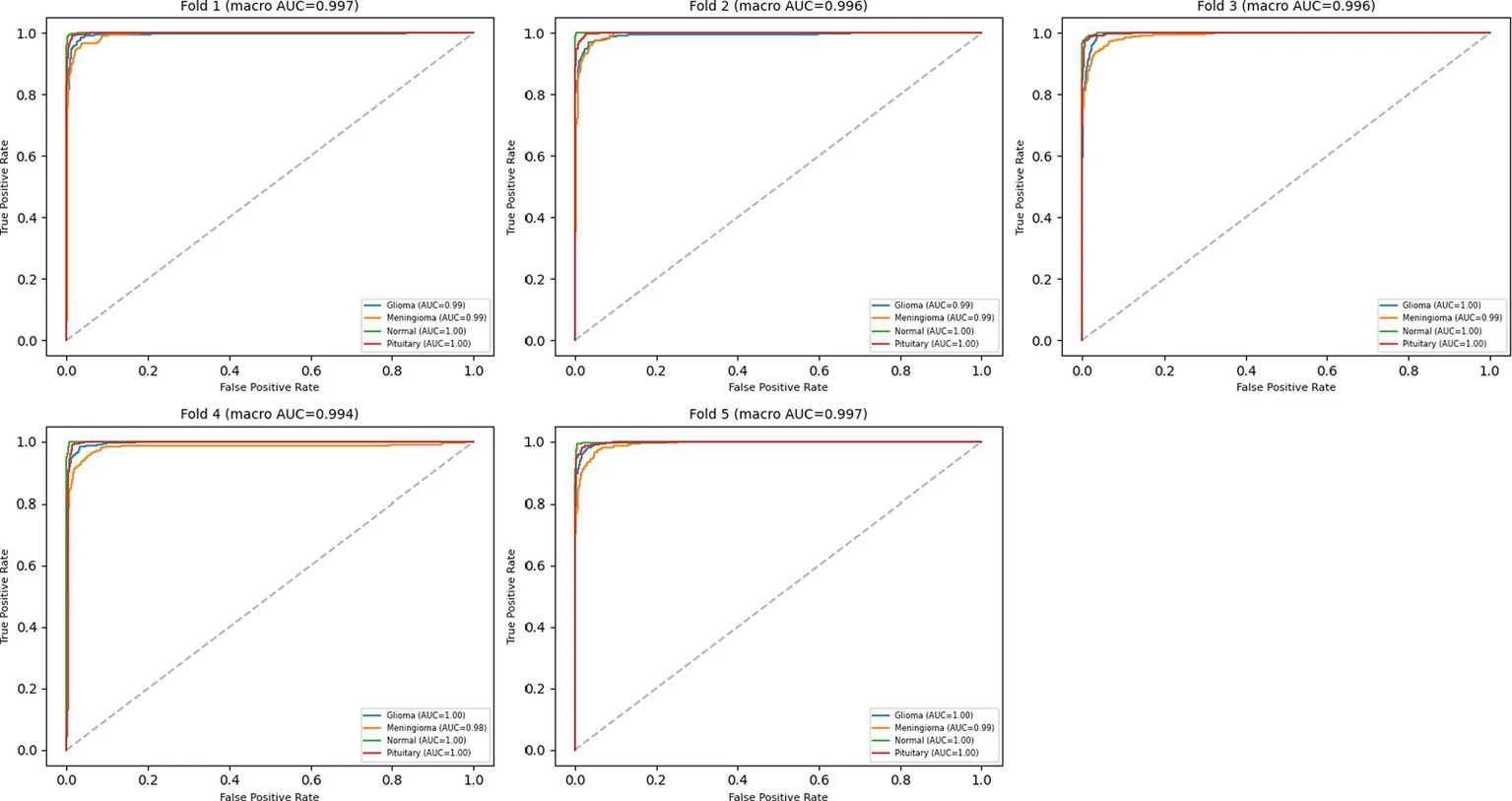
Fold-wise ROC curves of CNN-SVM under 5-fold cross-validation.

**Figure 24:**
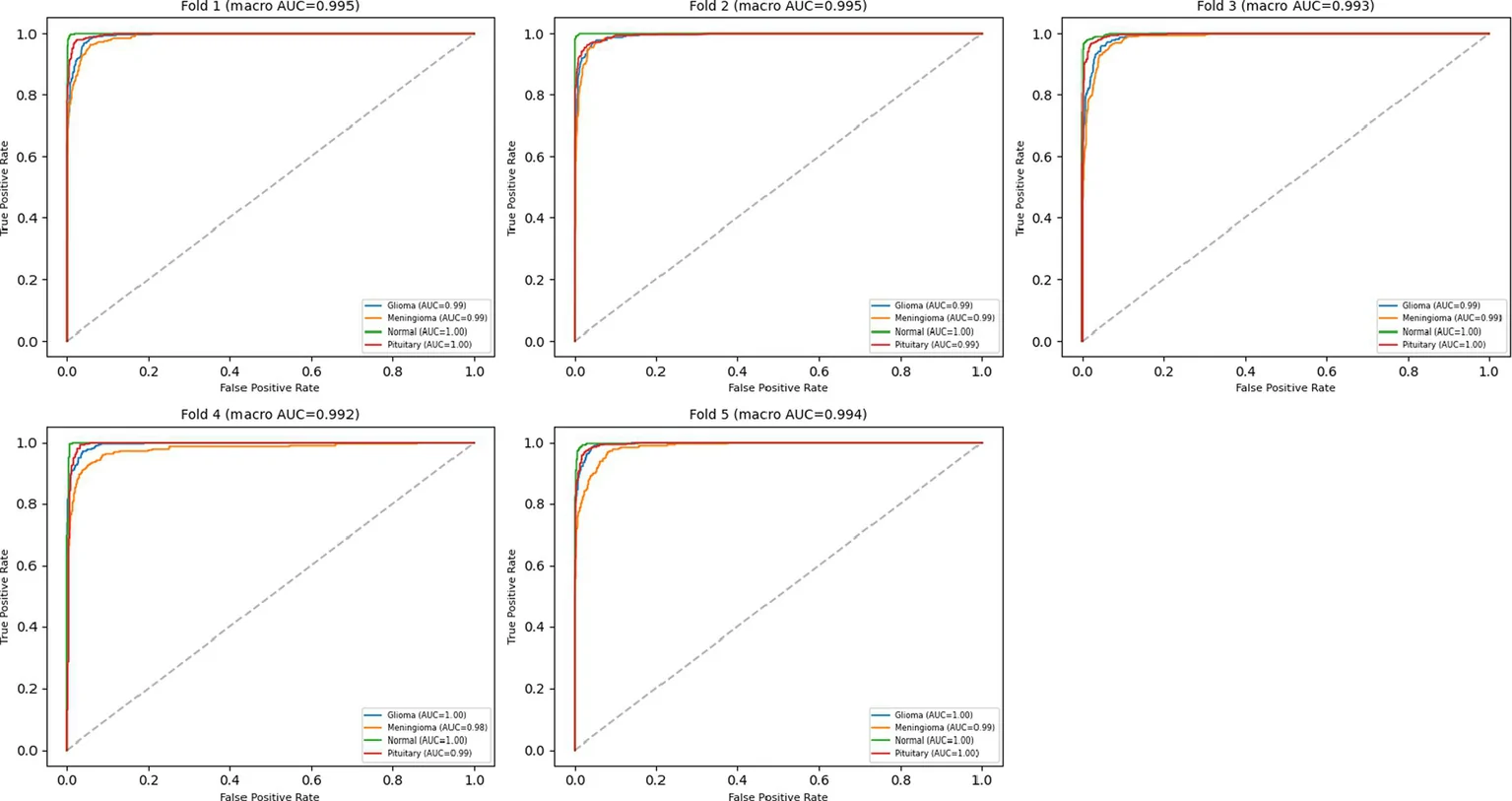
Fold-wise ROC curves of CNN-RF under 5-fold cross-validation.

Every configuration achieved a macro-averaged AUC above 98.9% in every fold, and all 20 curve families hugged the upper-left corner of the unit square, confirming that the MICA-enhanced embedding was close to linearly separable, irrespective of the decision rule applied on top of it. The ordering among configurations is nevertheless stable and informative: CNN-SVM occupies the highest band in every fold (99.4–99.7%), followed closely by CNN-RF and the softmax head (99.2–99.5%), and CNN-KNN (98.9–99.4%), despite leading to hard-label accuracy. The quantization effect analyzed in Section 4.4.1 is made visible in curve form because the KNN traces ascend in a small number of visible discrete steps, whereas the SVM traces are smooth.

Per-class decomposition is more diagnostic than macro averaging. Both normal and pituitary tissues achieved an AUC of 100.00% in nearly all folds and configurations, suggesting that the scores of the two classes could separate the populations into positive and negative classes with high accuracy. Meningioma has been the lowest curve in all panels, 98 to 99%; followed by glioma, which was the second lowest. The ranking disparity occurs in the two classes that make up the most dominant confusion channel in Table 10, which is significant because it shows that it is not an error specific to the threshold level; rather, it results from an actual intersection between the two distributions. (a) In clinical practice, even though a 98% AUC is high in absolute terms, there is still an option to use a threshold level that favors sensitivity for the meningioma class, which is another justification for a permissible operating point adjustment that is possible because of the continuous-score decision layer.

#### 4.4.4 Precision–recall (PR) curves

In this subsection, the ROC analysis is supplemented by precision-recall curves, which do not depend on the true-negative population size and thus react much more sharply to the precision that can be achieved at high recall, which determines practicality when tuning the model to miss as few lesions as possible. The precision-recall curves for each class are shown in Figures 25–28.

**Figure 25:**
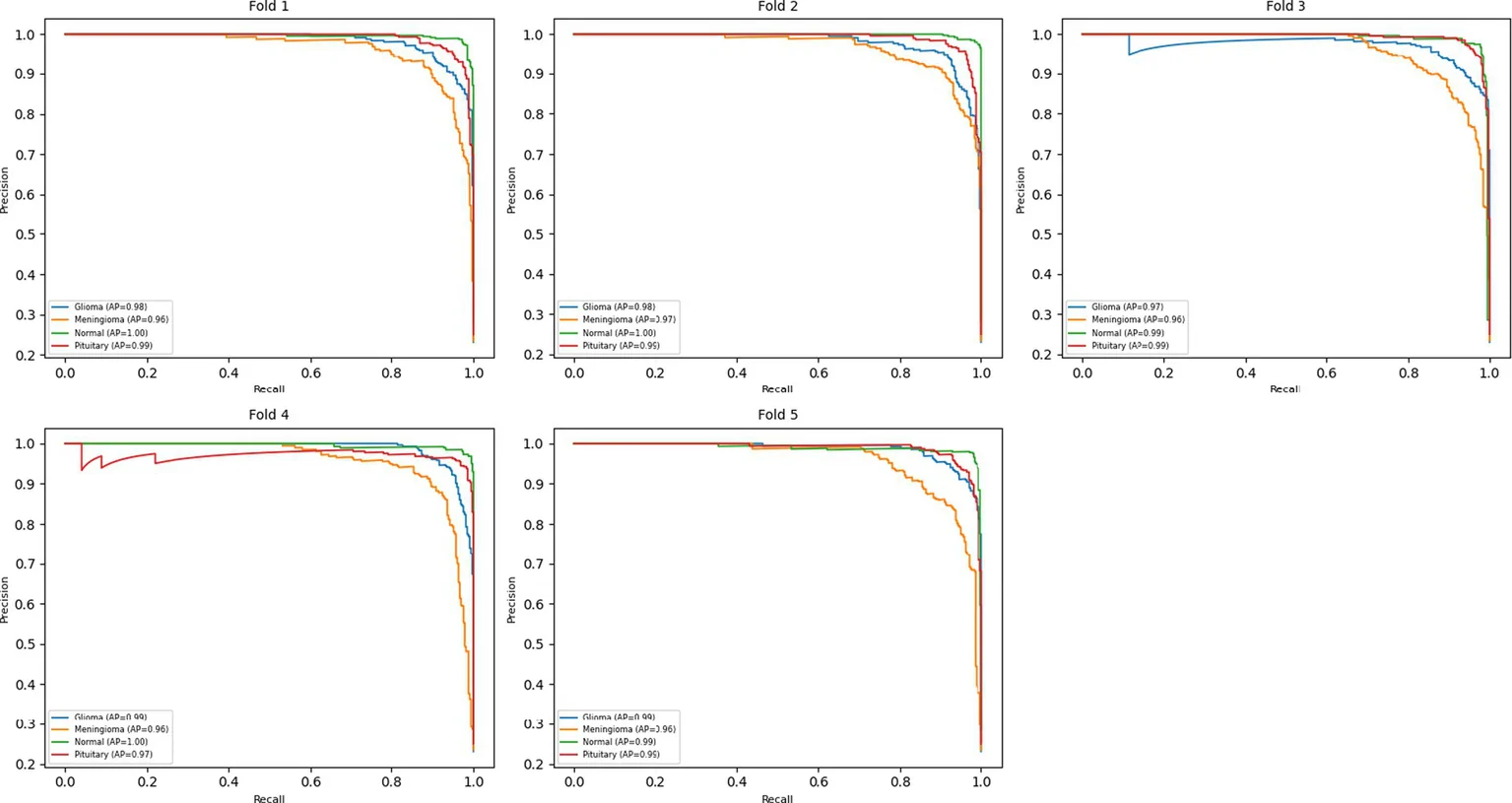
Fold-wise precision–recall curves of the CNN backbone with its native softmax head under 5-fold cross-validation.

**Figure 26:**
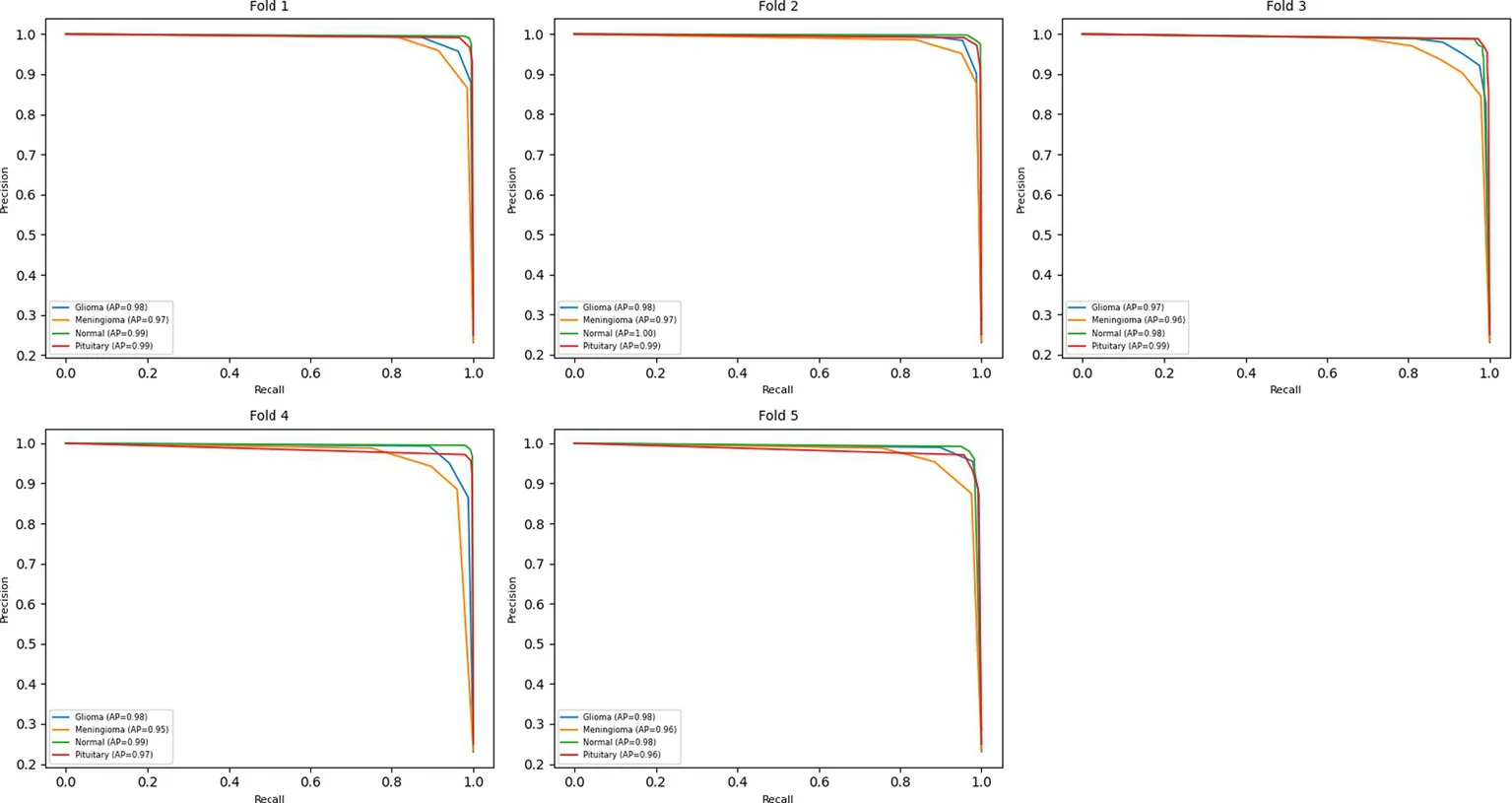
Fold-wise precision–recall curves of CNN-KNN under 5-fold cross-validation.

**Figure 27:**
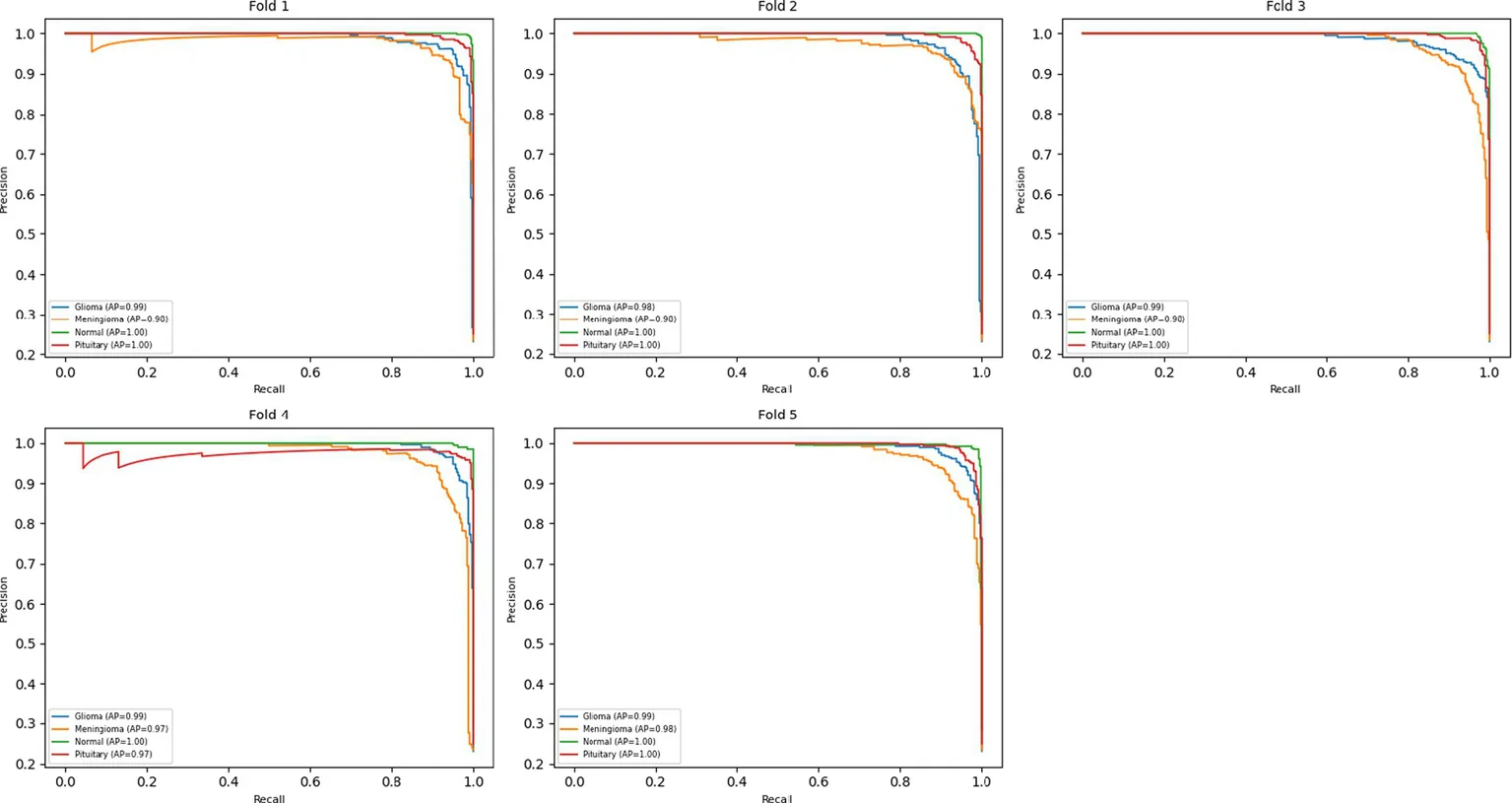
Fold-wise precision–recall curves of CNN-SVM under 5-fold cross-validation.

**Figure 28:**
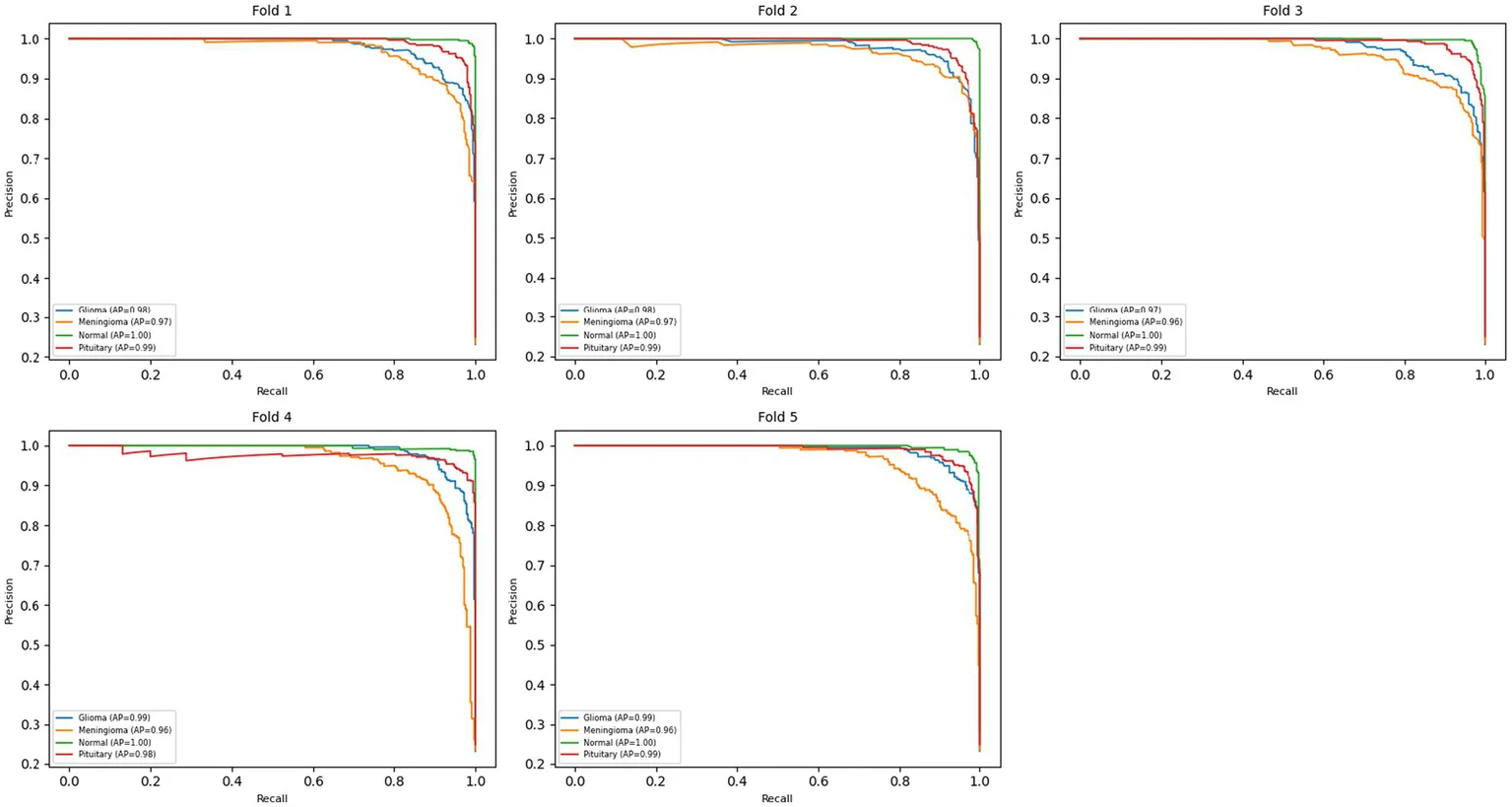
Fold-wise precision–recall curves of CNN-RF under 5-fold cross-validation.

The PR curves largely reproduce the AUC ranking of the classification results for a collection in which the imbalance of classes is only moderate but do so more discriminatively where it matters. Again, CNN-SVM scored the best, achieving an average precision of 97–98% for meningioma and 100% for the normal class in each fold. The softmax head and CNN-RF achieved 96 to 97% for meningioma, while CNN-KNN achieved the lowest score of 95% in the fourth fold. The shapes of the curves were as revealing as their areas. Normal and pituitary cases have precision that holds constant close to unity up to recall reaching approximately 90%, indicating that these classes may be operated at high sensitivity without paying a price in terms of additional false positives. In contrast, meningioma cases started to show a decline in precision below approximately 75% recall and then dropped dramatically above a 95% recall. This illustrates precisely the price of moving the operating point, as discussed at the end of the previous subsection: increasing the sensitivity of the meningioma class to the high nineties implies a sacrifice of precision, and the cases gained by this trade are mainly gliomas.

A further structural detail visible in the CNN-KNN panels is the near-vertical collapse of precision at maximum recall, which is an artifact of the discrete neighbor-vote score: once the threshold falls below the smallest attainable vote fraction, every remaining case is admitted simultaneously, and precision drops abruptly rather than degrading gracefully. The smooth decay of the CNN-SVM curves over the same interval is a practical manifestation of this distinction. Taken with the ROC evidence, the cross-validated PR analysis therefore converges on the conclusion already reached from the hold-out data, but now supported by five independent partitions: CNN-KNN is the preferred head when a single categorical diagnosis is required, whereas CNN-SVM is the preferred head wherever the prediction score itself has clinical meaning.

### 4.5 Computational complexity

This subsection profiles the computational demands of the shared CNN backbone—its parameter count, arithmetic cost, weight and activation memory, and inference latency on both CPU and GPU hardware—and considers what those figures imply for deployment in resource-constrained clinical environments.

With only 107,076 parameters—occupying a mere 0.20 MB in half precision—the proposed backbone is one to three orders of magnitude smaller than commonly used medical-imaging backbones of comparable depth (VGG16 ≈ 138 M parameters, ResNet-50 ≈ 25.6 M, MobileNetV2 ≈ 3.4 M), which is a direct consequence of the depth-wise separable convolutions used throughout every encoding stage. Despite this compact parameter budget, the arithmetic cost per image (≈0.88 GFLOPs) remains within the range typically associated with lightweight mobile-oriented architectures. Table 7 profiles the inference on both classes of hardware because the two correspond to different deployment scenarios. On CPU alone, the model sustains 23.7 images per second at a batch size of 16, with a median per-image latency of 42.2 ms, which is adequate for near-real-time, slice-by-slice screening at the point of care rather than for large-scale batch reprocessing; the per-image latency at this batch size exceeds that of smaller batches because CPU execution does not realize the parallelism-dependent efficiencies of batched inference. On an NVIDIA A100 GPU, the same network completes an image in 4.54 ms at the identical batch size, corresponding to 220.1 images per second and a 9.3-fold acceleration over the CPU configuration. The manner in which GPU efficiency depends on batch size is instructive: single-image inference is dominated by kernel-launch and host-to-device transfer overhead and therefore realizes only 16.5 images per second, whereas throughput rises to 117.9 images per second at a batch of 8, to 220.1 at 16, and saturates near 295.4 at 32, beyond which no further gain is observed. Batched execution is thus essential if the arithmetic frugality of the architecture is to be converted into wall-clock benefit, and a batch of 16 to 32 represents the point at which the latency and memory occupancy are jointly optimized. Expressed in workflow terms, a complete 25-slice MRI volume is classified in approximately 1.1 s on the CPU and 0.11 s on the GPU; thus, neither configuration imposes a clinically relevant delay. The CPU path establishes that the framework is deployable without dedicated accelerator hardware, whereas the GPU path additionally renders the whole-cohort retrospective analysis tractable. The gap between the largest single activation tensor (128.0 MB) and the actual peak resident memory of the process (≈1.36 GB) indicates that at moderate batch sizes, the practical deployment footprint of the model is governed substantially more by activation storage and framework-level overhead than by the intrinsically small parameters and FLOP budget of the network. The peak GPU memory of 4.93 GB recorded at the same batch size is likewise attributable to the workspace reservations and caching-allocator behavior of the accelerated runtime rather than to the model itself, and remains comfortably within the capacity of any contemporary clinical-grade accelerator. This distinction is relevant for selecting appropriate batch sizes for constrained clinical or edge deployments. This backbone cost is shared by all four classifier heads; however, the incremental inference cost of the decision layer differs qualitatively. KNN requires distance computations against the stored training embeddings, so its inference cost increases with the number of stored training samples. In contrast, SVM prediction depends primarily on the number of support vectors, while RF prediction depends on the number and depth of its trees. Therefore, KNN is particularly attractive for the present compact embedding and dataset size, whereas SVM or RF may become more computationally favorable as the number of stored training samples increases substantially in future deployments Table 11.

**Table 11:** McNemar’s test comparing the CNN baseline against each hybrid classifier (hold-out set).

Comparison (A vs. B)	b (A right, B wrong)	c (A wrong, B right)	*χ*^2^ statistic	*p*-value	Significant (*α* = 0.05)
CNN vs. CNN-KNN	16	57	21.92	2.85 × 10^−6^	Yes
CNN vs. CNN-SVM	7	43	24.50	7.43 × 10^−7^	Yes
CNN vs. CNN-RF	26	20	0.54	0.461	No

### 4.6 Statistical significance

This subsection establishes whether the accuracy differences reported above are large enough to be distinguished from sampling noise using a paired per-sample hypothesis test and a bootstrap confidence interval for the strongest hybrid configuration.

Accuracy points estimate alone do not indicate whether a CNN hybrid classifier is reliably better than the CNN-only baseline or whether the observed gap could plausibly be attributed to chance. Therefore, two complementary tests were applied to the hold-out test set. McNemar’s test, a paired test appropriate for two classifiers evaluated on the same samples, was applied to CNN versus each of CNN-KNN, CNN-SVM, and CNN-RF, comparing how often each pair of models is uniquely correct or incorrect on a per-sample basis. A paired bootstrap confidence interval was then constructed for the accuracy improvement of the strongest classifier, CNN-KNN, over the CNN baseline by resampling the hold-out set with replacement 2,000 times.

McNemar’s test confirms that both CNN-KNN and CNN-SVM correct significantly more CNN errors than they introduce (*p* < 0.001 in both cases), whereas the CNN-RF difference is not statistically significant (*p* = 0.461): CNN-RF flips almost as many previously correct predictions to incorrect (26) as it fixes (20), so its small apparent accuracy change is not distinguishable from chance at this sample size, corroborating the pattern already observed in the hold-out and ranking-based results. A complementary bootstrap analysis for the strongest classifier, CNN-KNN, reinforces this picture: resampling the hold-out set 2,000 times yields an observed accuracy improvement of 2.92 pp. over CNN, with a 95% confidence interval of [1.71, 4.13] pp. that excludes zero (Figure 29), confirming that CNN-KNN’s advantage is unlikely to be attributable to sampling noise in this particular hold-out split.

**Figure 29:**
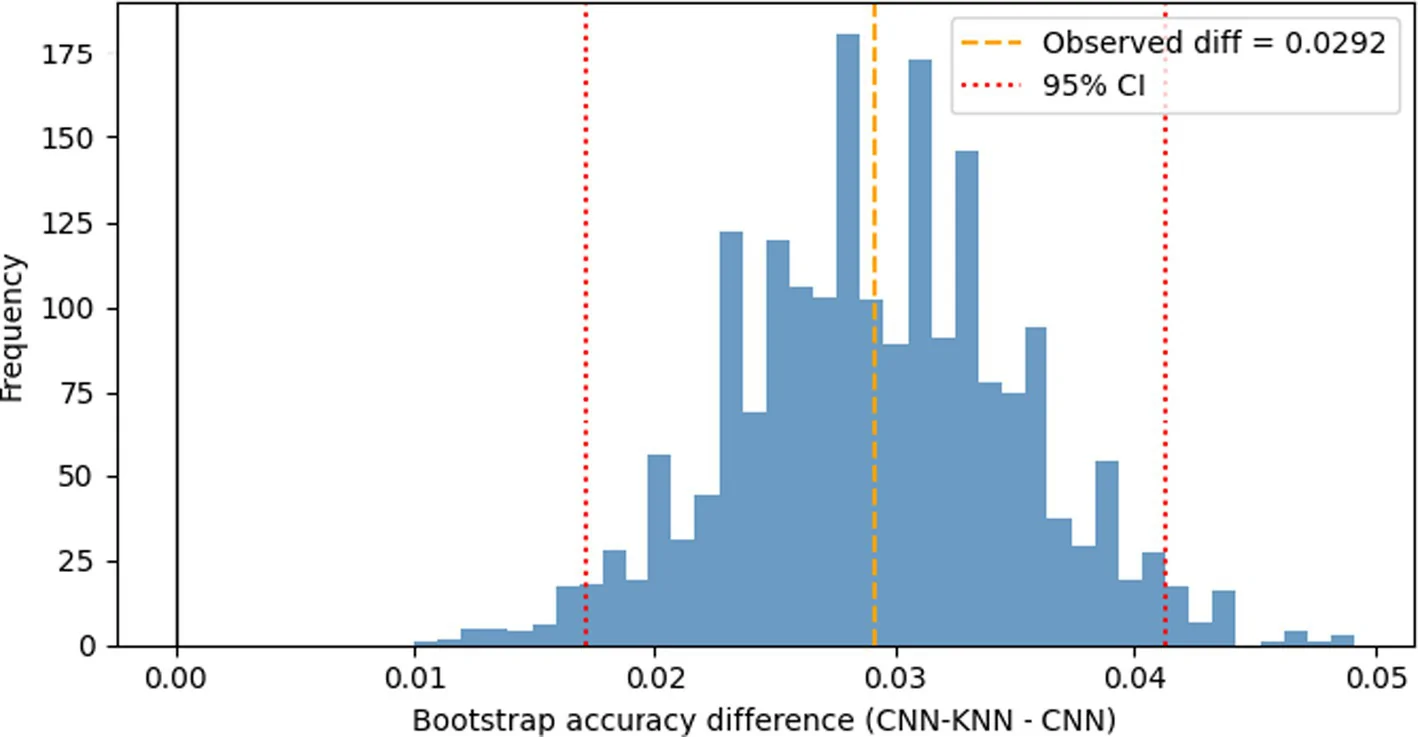
Bootstrap distribution of the hold-out accuracy difference between CNN-KNN and CNN (2,000 resamples).

Because a point estimate conveys no information about its own precision, two-sided 95% confidence intervals were additionally computed for the hold-out accuracy of every configuration using the Wilson score method, which is preferable to the normal approximation at proportions close to unity. On the 1,405-image hold-out partition, these intervals are 93.67% [92.27, 94.82] for the CNN softmax head, 96.58% [95.50, 97.41] for CNN-KNN, 96.23% [95.10, 97.10] for CNN-SVM, and 93.24% [91.80, 94.44] for CNN-RF. The corresponding 95% intervals for the fold-averaged cross-validated accuracies reported in Table 12, obtained from the Student t-distribution with four degrees of freedom, are [93.21, 94.69], [95.07, 97.13], [94.96, 96.38], and [93.10, 94.88], respectively.

**Table 12:** Agreement between the hold-out and 5-fold cross-validation estimates of classification accuracy.

Configuration	Hold-out accuracy (%)	5-FCV mean ± SD (%)	5-FCV fold range (%)	Δ (5-FCV—hold-out, pp)	Hold-out estimate within fold range
CNN	93.67	93.95 ± 0.60	93.38–94.66	+0.28	Yes
CNN-KNN	96.58	96.10 ± 0.83	95.30–97.22	−0.48	Yes
CNN-SVM	96.23	95.67 ± 0.57	94.95–96.44	−0.56	Yes
CNN-RF	93.24	93.99 ± 0.72	92.88–94.59	+0.75	Yes

Two observations were made. The interval for CNN-KNN overlaps with that for CNN-SVM under both protocols; therefore, the present analysis does not provide evidence that either hybrid head has a statistically established accuracy advantage over the other. In contrast, the intervals for both hybrid heads were disjoint from those of the softmax and RF heads on the hold-out partition. It should be emphasized that these marginal intervals are deliberately conservative for the purpose of comparing configurations, since they disregard the fact that all four heads are evaluated on the same images. The paired McNemar and bootstrap analyses reported above exploit that pairing and therefore constitute the appropriate basis for the significance claims made in this study. Throughout the remainder of the paper, accuracy differences smaller than approximately one percentage point are described as indistinguishable rather than as an advantage of one configuration over another.

### 4.7 Comparison of the hold-out and 5-fold cross-validation protocols

Two independent calculations for the same quantity were provided in the previous subsections using different resampling procedures. This subsection provides a comparison of both results to address a question that cannot be addressed independently using either of the procedures: whether the results obtained in Section 4.3 from the 80:20 split are due to the properties of the framework or the result of a particular advantageous split of the dataset. The comparison was performed numerically using the aggregated data in Table 12 and visually using the plots in Figure 30.

**Figure 30:**
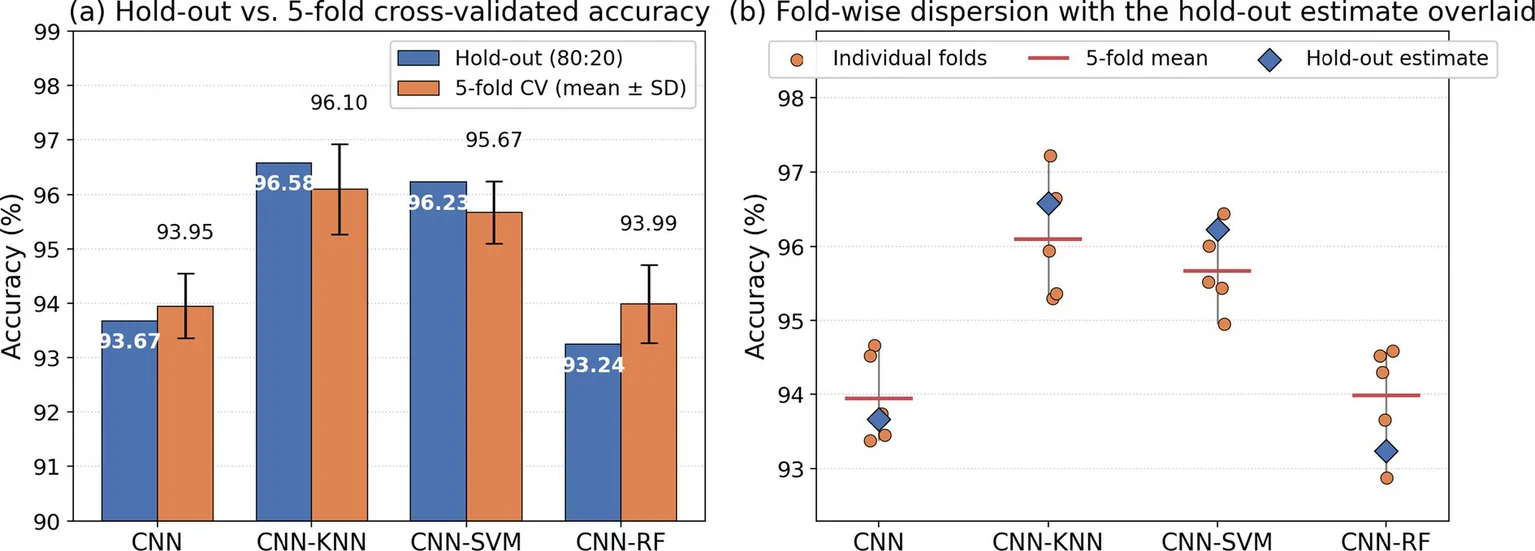
Comparison of the hold-out and 5-fold cross-validation protocols: **(a)** Hold-out accuracy against the cross-validated mean with standard-deviation error bars; **(b)** Fold-wise dispersion with the hold-out estimate overlaid.

The two methods yielded almost identical results. The greatest difference between the hold-out accuracy score and the respective fold mean was 0.75 percentage points, while each difference fell well within the range of one standard deviation from the respective fold distribution, and the hold-out score resided in the same range as that of the folds in all four configurations. Because both the hold-out split and the cross-validation splits were selected independently of each other, this similarity is not a matter of design but rather serves as a clear demonstration of the general nature of the observed performances.

A graphical depiction of the connection between the hold-out split and fold means is shown in panel (b) of Figure 30. In each of the four configurations, the point of the hold-out diamond lies within the range of the five-fold points and within half a standard deviation from the fold mean in three of the four cases. Moreover, this figure highlights a consistent and significant asymmetry in the direction of the discrepancy. The hold-out split shows a slight tendency to be overly optimistic when it comes to both hybrid heads (−0.48 pp. for CNN-KNN and −0.56 pp. for CNN-SVM) and is slightly pessimistic for the other two configurations (+0.28 pp. for the softmax head and +0.75 pp. for CNN-RF). The result was an increase in the substitution gain owing to the single-split assessment. Thus, the difference between the CNN-KNN method and the softmax head is 2.91 pp. using the hold-out approach and 2.15 points in cross-validation, and the corresponding CNN-SVM advantage decreases from 2.56 to 1.72 points. The RF comparison underwent an outright sign reversal, from −0.43 points on the hold-out set to +0.04 points under cross-validation, which is precisely the behavior expected of a difference that is genuinely null and reinforces the non-significant McNemar result of Section 4.6 from an entirely independent perspective.

Two further points of agreement deserve emphasis because they concern the qualitative conclusions of this study rather than its headline numbers. First, the ranking of the four configurations is identical under both protocols, with CNN-KNN first, CNN-SVM second, and CNN-RF and the softmax head statistically inseparable. Second, the dissociation between hard-label accuracy and threshold-independent ranking quality—CNN-KNN leading on accuracy while CNN-SVM leads on AUC—was reproduced without attenuation, with CNN-SVM recording a macro-AUC of 99.56% on the hold-out set and 99.59 ± 0.11% across folds. The recommendation that the decision layer be selected according to whether the downstream task consumes a label or score, therefore, rests on two independent evaluations rather than one.

Two limitations of this comparison should be stated. The cross-validated estimates carry a mildly optimistic bias because, in the absence of a third partition, each fold’s evaluation split also supplied the monitoring signal for learning-rate annealing and early stopping. The bias is small since only the stopping epoch rather than any model parameter was selected on that signal, and it was applied identically to all four heads; therefore, the between-configuration comparisons that carry the study’s conclusions are unaffected. Conversely, the cross-validated estimates are also conservative in one respect, since each backbone is trained on 80% of the corpus rather than the whole of it, and the learning curves in Section 4.4.2 suggest that the architecture had not yet saturated its data budget. The two effects act in opposite directions, and the close agreement documented in Table 12 indicates that neither dominates corpus size. Where a single figure must be quoted, the cross-validated mean is the more defensible estimate of expected performance on unseen data, whereas the holdout results retain their value as the setting in which the per-sample paired statistical tests of Section 4.6 and the component ablations of Section 4.8 were conducted on a common, untouched partition.

### 4.8 Ablation study

To quantify the contribution of each architectural and preprocessing component to the final CNN-KNN accuracy, eight variants of the pipeline were trained and evaluated on the same hold-out split, changing exactly one component at a time relative to the full model: removing MICA contrast enhancement entirely (raw resized images), retaining only a single MICA Gaussian scale (σ = 15, 30, or 60) instead of averaging all three, disabling the dilated depth-wise convolution (dilation rate = 1), removing the SE attention block, removing the residual skip connection, and a bare backbone with neither SE nor residual connections. Each variant was retrained with early stopping and paired with the same classifier configuration as the CNN-KNN. Table 13 presents these results in detail.

**Table 13:** Ablation study: contribution of each architectural and preprocessing component (hold-out set).

Variant	CNN accuracy (%)	CNN macro-F1 (%)	CNN-KNN accuracy (%)	CNN-KNN macro-F1 (%)
Full model (multi-scale MICA)	93.67	93.39	96.58	96.49
No SE attention	87.19	86.61	95.73	95.54
MICA *σ* = 60 only	87.33	86.88	94.73	94.53
MICA *σ* = 30 only	87.47	87.01	94.45	94.24
MICA *σ* = 15 only	87.69	87.30	94.38	94.16
No MICA	86.19	85.53	93.52	93.25
No residual connection	83.20	82.27	93.45	93.19
Bare backbone (no SE, no residual)	82.70	82.00	93.38	93.13
No dilation	85.77	85.04	93.31	93.06

Figure 31 shows the CNN-KNN accuracy achieved by each variant relative to the full model, making the relative contribution of each component easier to compare at a glance.

**Figure 31:**
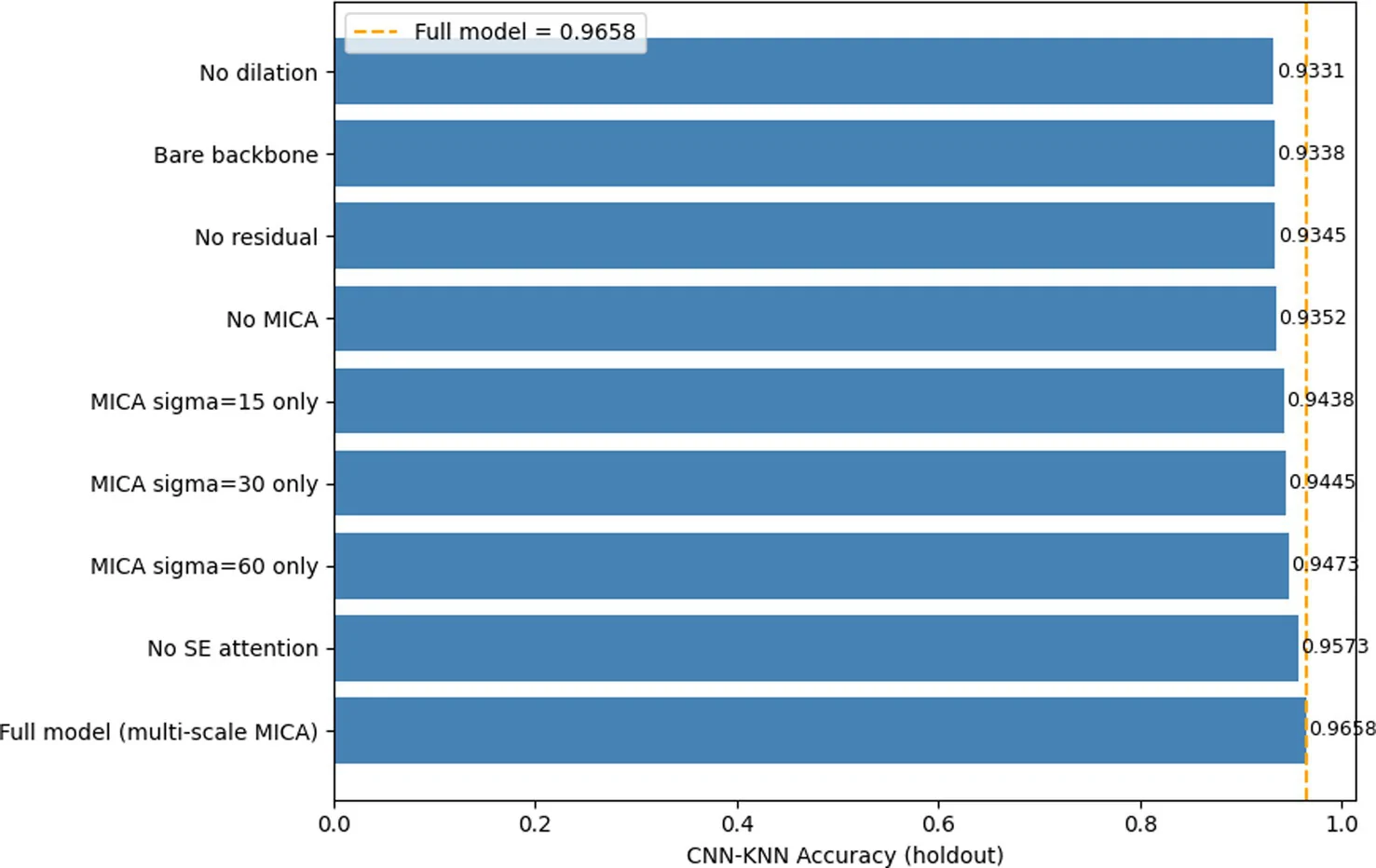
Ablation study: Contribution of each architectural and preprocessing component to CNN-KNN accuracy.

As shown in Figure 31 and Table 13, ranking the accuracy drop relative to the full model identified dilation as the single most consequential component (−3.27 pp. when disabled), narrowly ahead of the bare-backbone configuration (−3.20 pp) and residual connection (−3.13 pp). Removing MICA contrast enhancement entirely costs a comparable 3.06 points, whereas restricting MICA to any single Gaussian scale costs less (1.85–2.21 points), confirming that the three scales are complementary, rather than redundant. The individual-scale results also show that no single Gaussian scale reproduces the performance of the complete three-scale configuration, providing classification-level evidence that the selected scales contribute complementary information to the final prediction. SE attention is the least individually consequential component (−0.85 points), indicating that channel recalibration refines an already strong representation rather than being solely responsible for the discriminative capacity of the network. Because dilation, residual connections, and MICA each contribute more than any single MICA scale or the SE block individually, spatial receptive-field expansion and gradient-flow stability emerge as the primary architectural drivers of the backbone’s performance, with contrast normalization providing an independent and comparably sized contribution, and channel attention acting as a smaller refinement layered on top of both.

### 4.9 Feature interpretability

Interpretability is examined here at two complementary levels: the geometry of the feature space upon which every decision layer operates and the spatial evidence within the image from which the feature space is derived.

#### 4.9.1 Feature-space visualization

The comparison of decision layers undertaken in Sections 4.3 and 4.4 rests on the premise that the 256-dimensional embedding produced by the global max-pooling layer is already organized by diagnostic category, so that the residual differences between heads reflect the shape of their decision boundaries rather than the quality of the underlying representation. To examine this premise directly rather than inferring it from downstream accuracy, the hold-out embeddings were projected into two dimensions using t-distributed stochastic neighbor embedding (t-SNE; a 50-component PCA pre-reduction, perplexity = 30, 1,000 iterations, Euclidean metric, fixed random seed) and, as a safeguard against the known sensitivity of *t*-SNE to its perplexity setting, uniform manifold approximation and projection (UMAP; 15 neighbors, minimum distance 0.1), the latter additionally providing an out-of-sample transform that t-SNE does not support. Four complementary views of the resulting two-dimensional space were examined in turn: the effect of the backbone on class geometry relative to the raw input (Figure 32), the partition each classifier head imposes on that geometry (Figure 33), the subset of instances on which the four heads disagree (Figure 34), and the shape of the decision boundary each head constructs within the projected space (Figure 35).

**Figure 32:**
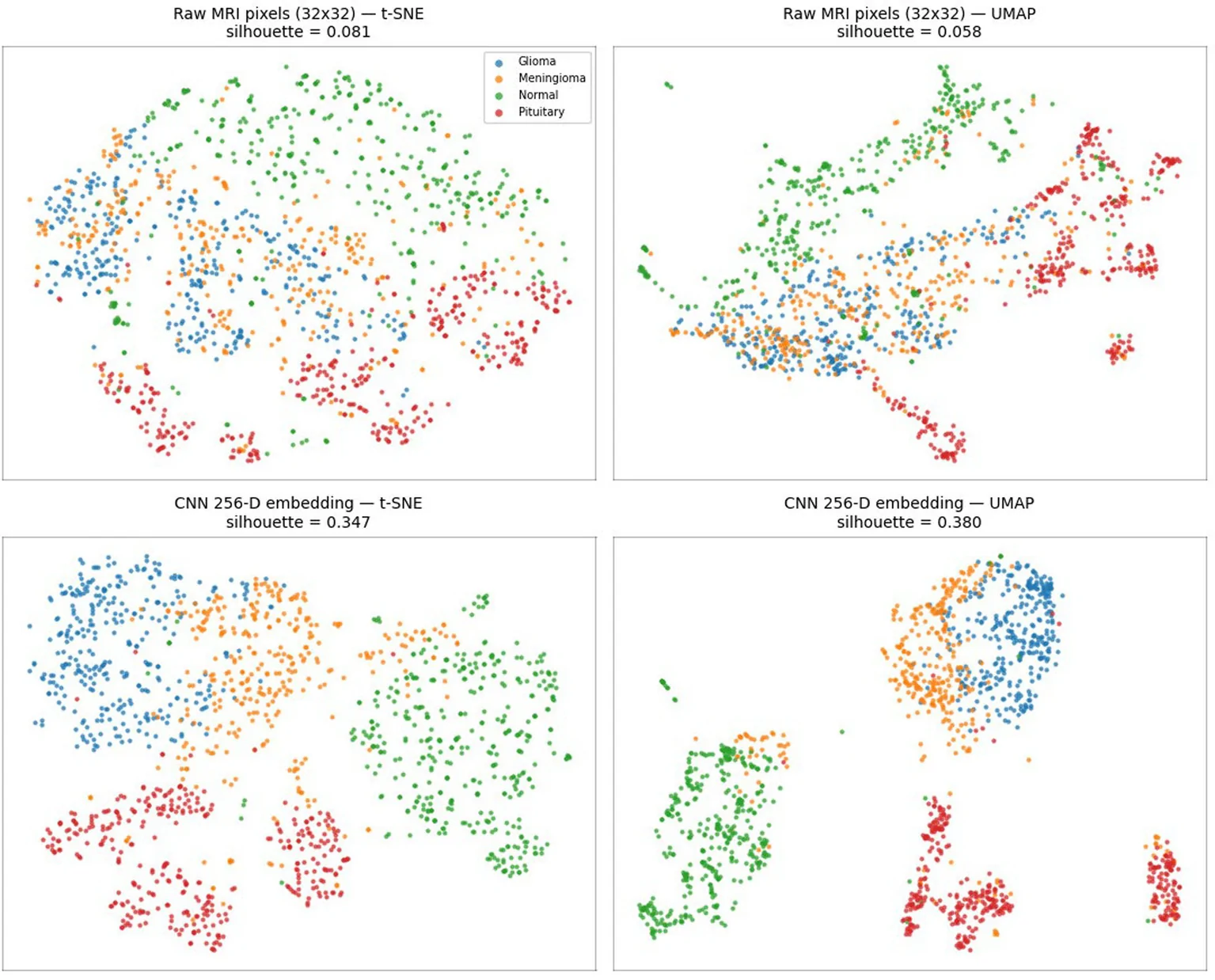
Test-set feature space before and after the MICA + SE-Residual CNN backbone: raw 32 × 32 MRI pixels (top row) versus the 256-D CNN embedding (bottom row), each projected with t-SNE (left) and UMAP (right) and coloured by ground-truth class.

**Figure 33:**
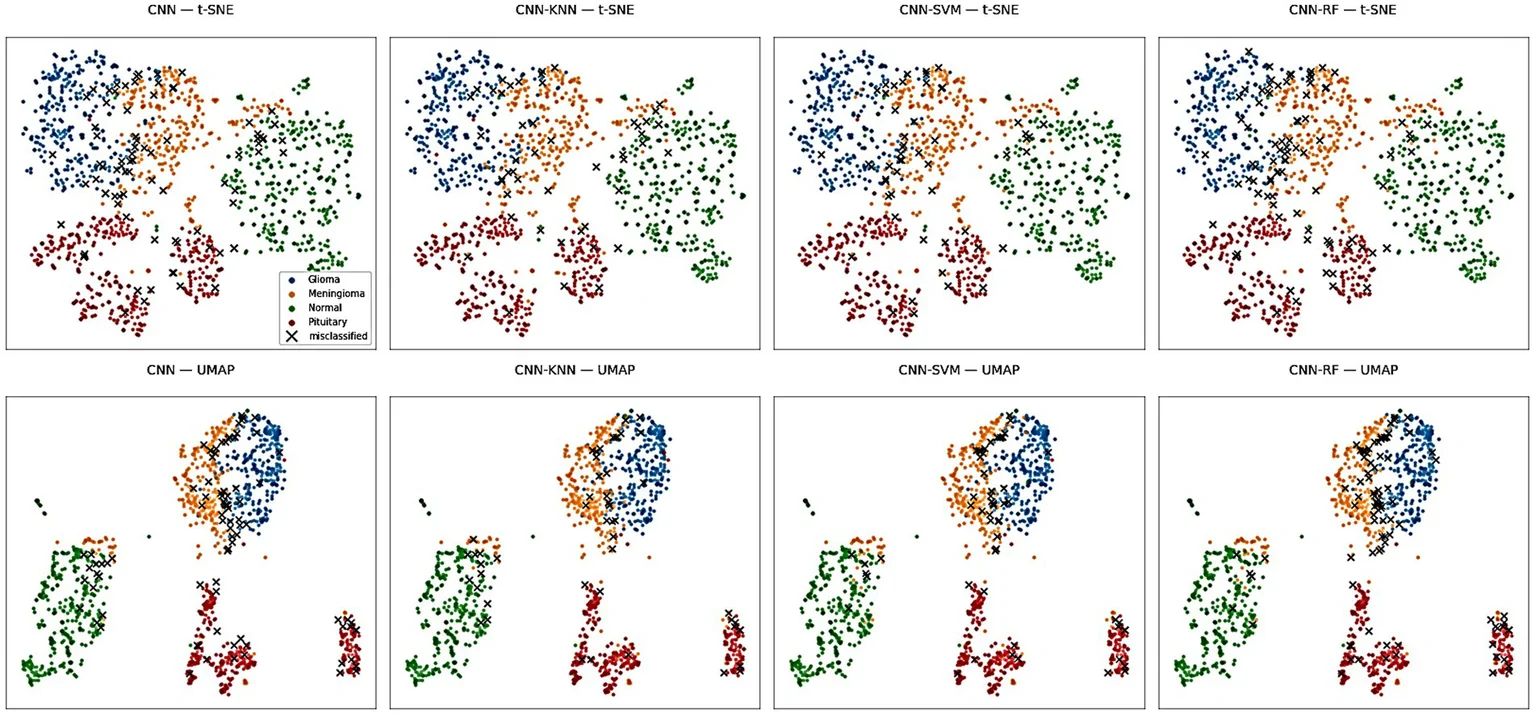
Same 2-D map as Figure 32 (CNN embedding only), recoloured by each classifier head’s predicted class — CNN, CNN-KNN, CNN-SVM, and CNN-RF, under t-SNE (top row) and UMAP (bottom row); black × marks a misclassified point.

**Figure 34:**
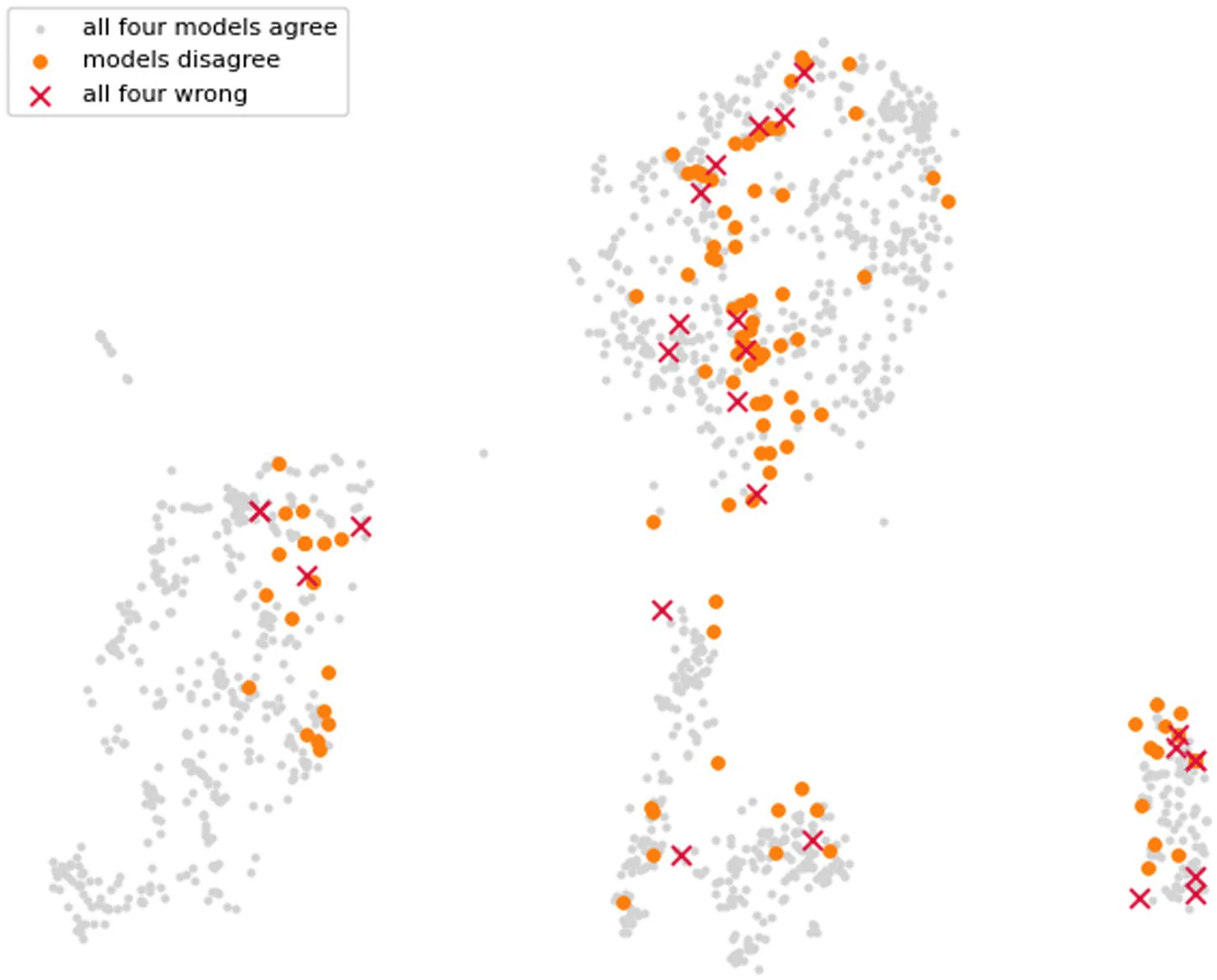
Disagreement map (UMAP): Grey points denote test scans on which all four classifier heads agree, orange points denote the 108 of 1,405 hold-out scans that split the four heads, and red × marks denote the 25 scans on which every head is wrong.

**Figure 35:**
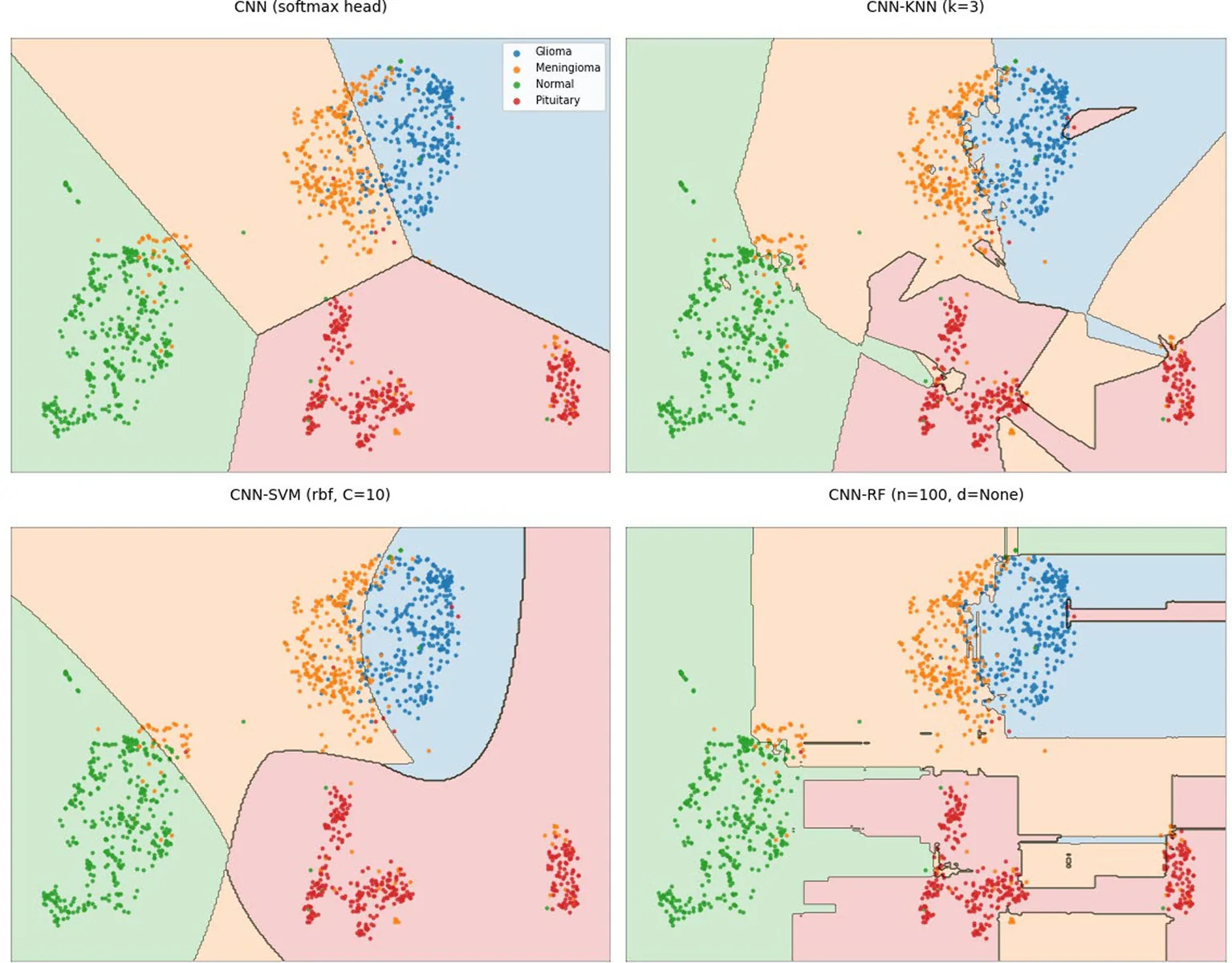
Decision regions in the 2-D UMAP embedding for each classifier head, fit on the projected training points and evaluated on the projected test points: CNN softmax head (multinomial logistic regression), CNN-KNN (*k* = 5), CNN-SVM (RBF kernel, *C* = 10), and CNN-RF (300 trees).

Figure 32 shows the representation learning performed by the backbone directly visible by projecting the same held-out scans twice: once from the raw 32 × 32 pixel grid (1,024-D) and once from the 256-D embedding the CNN produces from it. In the raw-pixel projections, silhouette coefficients of 0.081 (t-SNE) and 0.058 (UMAP) indicate that the four tumor categories form only a weakly separated, substantially entangled cloud; the pituitary class is a partial exception, forming a loosely coherent arm even at this stage, consistent with its stereotyped sellar location noted in Section 1. Passing the same scans through the trained backbone raised the silhouette coefficient to 0.347 under t-SNE and 0.380 under UMAP, and the embedding resolved into four visibly distinct clusters. This is consistent with the class-wise behavior documented quantitatively in Table 8 and the confusion matrices in Figure 8. The pituitary and normal categories occupy compact, well-separated regions of the embedding, in agreement with their recall of 99.15 and 98.00%, respectively, under CNN-KNN, whereas the glioma and meningioma populations remain adjacent and share a diffuse interface even after feature extraction, corresponding to the bidirectional glioma–meningioma confusion that, per the confusion matrices in Figure 8, accounts for 43.8% of CNN errors (39 of 89), 37.5% of CNN-KNN errors (18 of 48), 50.9% of CNN-SVM errors (27 of 53), and 56.8% of CNN-RF errors (54 of 95). Therefore, the embedding is structured by diagnostic category rather than by an incidental property of the acquisition, and the four-fold increase in the silhouette score attributable to the backbone quantifies, rather than merely illustrates, the representational gain that the classical heads in Sections 4.3 and 4.4 build upon.

Figure 33 recolors this same embedding by the predicted, rather than true, class for each of the four decision layers, so that the errors of each classifier can be read directly from the cluster geometry established in Figure 32. Cross-referencing these predictions against the confusion matrices in Figure 8 reproduces the ordering already established in Table 8: CNN-KNN misclassified the fewest scans (48 of 1,405; 96.58% accuracy), followed by CNN-SVM the next fewest (53; 96.23%), the bare CNN softmax head (89; 93.67%), and CNN-RF (95; 93.24%). This ordering is invariant to whether the underlying projection is t-SNE or UMAP. In every panel, the black × markers denoting misclassification are not scattered uniformly across the map but concentrate almost exclusively along the glioma–meningioma interface identified in Figure 32, with only a small number of additional errors near the boundary separating the normal and pituitary regions. The interior of each cluster was essentially error-free for every classifier. CNN-KNN and CNN-SVM visibly recover more of the interface region into the correct color than the CNN and CNN-RF panels do, which is the visual counterpart of their lower error counts and previews the decision region analysis of Figure 35.

Because the four heads share a common embedding, Figure 33 leaves open the question of whether the classifiers tend to err on the same or different instances. Figure 34 directly answers this by marking every test scan on which the four predictions are not unanimous. Of the 1,405 hold-out scans, 108 (7.7%) split the four models, and a further 25 (1.8%) defeated all four simultaneously. These points are not distributed randomly across the embedding but sit almost entirely along the same glioma–meningioma interface and, to a lesser extent, along the narrow seam between the normal and pituitary arms—precisely the regions already flagged as ambiguous in Figures 32, 33. The 25 scans defeating every classifier, irrespective of the shape of its decision rule, cluster in this same narrow region, indicating that this residual disagreement reflects a property of the underlying feature geometry itself—scans whose embeddings fall genuinely between two class manifolds—rather than an idiosyncrasy of any single classifier’s decision surface.

Figure 35 completes the analysis by refitting each decision rule inside the two-dimensional UMAP embedding itself, using the hyper parameters selected in Section 4.2, so that the shape of each classifier’s decision boundary, rather than only its errors, can be directly inspected. The softmax head of the CNN is exact in this reduced space because its classification layer is a single dense unit on the embedding, that is, multinomial logistic regression, and its boundaries are consequently linear by construction; this rigidity is visible as a single straight partition cutting directly through the glioma–meningioma interface, which is why it recovers the lowest 2-D-space accuracy of the four heads (87.83%). By contrast, CNN-KNN is a purely local, non-parametric rule, and its boundary conforms tightly and irregularly to the true cluster shapes, including the narrow neck between glioma and meningioma, yielding the highest 2-D-space accuracy (90.82%). CNN-SVM occupies an intermediate position: its RBF kernel produces a single smooth, curved boundary through the same interface that is more flexible than the softmax line but does not fragment into the small pockets KNN admits, giving a comparable accuracy (90.53%). CNN-RF partitions the space into an axis-aligned mosaic of small rectangular regions, a signature of its underlying decision-tree splits; the resulting boundary is the most locally accurate in this reduced space (91.25%) but is visibly the most fragmented and least smooth, consistent with the RF head’s comparatively weaker generalization once threshold-independent ranking is considered in Section 4.3. The higher RF accuracy in the reduced 2-D projection should not be interpreted as evidence of superior performance in the original 256-dimensional embedding; it reflects the behavior of the classifiers after projection and is therefore used only to illustrate differences in decision-boundary geometry. The quantitative classifier comparison remains that reported for the original 256-dimensional embedding in Table 8 These figures are fit and evaluated entirely within the projected 2-D space and not the original 256-D embedding; therefore, the accuracies reported here illustrate the qualitative shape of each decision rule rather than reproducing the exact hold-out figures of Table 8 and are systematically lower than the latter because two dimensions retain only a fraction of the class-discriminative information present in the full embedding. Taken together with Figures 33, 34, this geometric evidence explains why the choice of decision layer matters in the particular way that it does: because the classes form locally coherent clusters, the local, non-parametric KNN rule and the margin-maximizing SVM rule both adapt readily to the true, non-linear shape of the interface, whereas the axis-aligned partitioning of RF is poorly matched to a boundary that is oblique in the coordinate system of the embedding, and the linear softmax head is poorly matched for the same reason. Thus, the visualization provides a geometric account of the classifier ordering reported in Sections 4.3, 4.4, and 4.6, rather than merely restating it in another form.

#### 4.9.2 Feature interpretation

In addition to quantifiable performance, the practical value of an automated diagnostic system hinges on the anatomical soundness of its predictions rather than on false associations. In this regard, the use of SmoothGrad-CAM++ (Omeiza et al., 2019), an extension of the Grad-CAM++ high-order gradient importance weights by the smooth noise averaging of SmoothGrad for eliminating speckling artifacts, on the output feature map right before the GlobalMaxPooling layer was analyzed. As this layer processes a 256-channel tensor similar to the classifier head embedding layer, the results are shown in Figure 2 for one representative holdout instance per class.

For the meningioma case, the attention map focused narrowly on the enhancing extra-axial lesion rather than the brain parenchyma or skull around it, suggesting that the model’s attention was focused on the correct anatomical region rather than on irrelevant anatomical features. For the normal case, activation is spread out over the ventricular and midline structures rather than localized to a point of focus, suggesting that the model validated the absence of a lesion by checking correct anatomy instead of simply “not finding anything.” The localization of attention is a necessary but insufficient step toward clinically trusting the model’s predictions, thus motivating the need for interpretability validation in the future.

In terms of clinical workflow, there are three implications of such localization behavior, apart from the value of providing qualitative reassurance. First, because the visualization is based on the same 256-channel tensor fed into all the classification heads studied here, the radiologist who looks at the SmoothGrad-CAM++ overlay actually analyzes the evidence base of CNN, KNN, SVM, and RF classifications, not only a particular one but all. Thus, visualization can be used as a universal second-reader tool, regardless of the choice of classification head used in a particular case. Second, the class-specific attention patterns observed in these representative cases are anatomically plausible and provide qualitative support for the relevance of the highlighted regions. For meningioma, the attention is concentrated around the enhancing extra-axial mass, whereas in normal cases it is distributed across broader anatomical landmarks rather than being focused on a suspected lesion. These patterns are consistent with the regions that would ordinarily be considered when interpreting such cases, but they should be regarded as qualitative evidence rather than confirmation of the model’s clinical reasoning. Third, the continued presence of anatomically grounded attention to the meningioma class, which is the most commonly confused class in Table 8, shows that the network’s residual errors in that particular class have a higher probability of being associated with the intrinsic diagnostic difficulty arising from the radiological signal rather than the fact that the model attends to the wrong parts of the image. This is because of the implications it would have on the potential gains to be made by improving that class, as it shows that additional diagnostic information in the form of contrast sequences or dural contact priors is required. However, the above remains qualitative since it only shows that the attention of the network is anatomically correct based on the sample inspected; it does not yet quantify the level of agreement between radiologists’ marked regions of interest over the whole test set or show whether there is a quantifiable calibrated confidence in predictions by the model.

### 4.10 Comparative analysis

Table 14 compares the proposed framework with the state-of-the-art methods reviewed in Section 2. Accuracy is used as the common basis of comparison because it is the only metric reported by every referenced study, and the experimental protocol adopted by each study is listed alongside it so that the terms on which the figures were obtained remain visible to the reader. Two entries are provided for the proposed framework, namely 96.10% under stratified five-fold cross-validation and 96.58% under the 80:20 hold-out split, so that it may be read against studies adopting either resampling scheme rather than only against those that favor it.

**Table 14:** Comparison of the proposed and state-of-the-art methods.

Method	Experimental protocol (train/test ratio in %)	Accuracy (%)
ResNet-18 (Tang and Teoh, 2023)	80/20	88.33
CLM-DL (Woźniak et al., 2023)	80/20	95.09
GNN (Gürsoy and Kaya, 2024)	80/20	93.68
FL-efficientNetB0 (Zhou et al., 2024)	80/20	80.17
SE-denseNet-121 (Mao et al., 2025)	85/15	95.60
Customized CNN (Dewage et al., 2024)	85/15	94.51
CFLM-Grad-CAM (Latif et al., 2025)	—	94.24
3D-CNN-RF (Yin and Wang, 2025)	80/20	95.00
CNN-SVM (Suryawanshi and Patil, 2024)	80/20	95.16
VGG16-ANN (Castelino et al., 2024)	80/20	84.00
EWHO-SqueezeNet (Kanchanamala et al., 2025)	5-FCV	93.20
GTLO-LeNet (Aluri and Imambi, 2024)	80/20	89.60
SCAOA-SegNet (Geetha et al., 2024)	80/20	93.00
Proposed method (CNN-KNN)	5-FCV	96.10
	80/20	96.58

The competing methods are classified into five groups. Transfer-learning approaches built on ImageNet-pretrained backbones, comprising ResNet-18 (88.33%), FL-EfficientNetB0 (80.17%), VGG16-ANN (84.00%), and SE-DenseNet-121 (95.60%), formed the largest group and spanned the widest range of outcomes. Because these backbones acquire their representational priors from natural color images, they carry an inductive bias that transfers imperfectly to the grayscale intensity and texture statistics of MRI, which plausibly explains why even the strongest of them remains below the proposed model despite employing SE-based channel recalibration. The difference is that the SE modules of SE-DenseNet-121 refine a pretrained backbone, whereas those of the proposed model are trained end-to-end alongside depth-wise separable convolutions directly on MICA-enhanced MRI; that entry is moreover obtained under an 85/15 split, which allots it a larger training partition than the 80/20 protocol used here.

Optimization-augmented lightweight networks, namely EWHO-SqueezeNet (93.20%), GTLO-LeNet (89.60%), and SCAOA-SegNet (93.00%), improve upon plain transfer learning through meta-heuristic hyperparameter search, but their accuracy remains below 94%, which is consistent with the limited representational capacity of the underlying shallow backbones rather than with any deficiency in the search strategy itself. Relational methods, namely CLM-DL (95.09%) and GNN (93.68%), introduce explicit reasoning between image regions and narrow the gap relative to the first group, although at the cost of higher computational expense and reduced transparency compared with the compact and fully convolutional design used here. The fourth group dispenses with pre-trained weights altogether: the customized CNN (94.51%) and CFLM feature fusion model with Grad-CAM (94.24%) confirm that a task-specific architecture trained from scratch is competitive with transfer learning, but neither couples such an architecture to a contrast-domain front end.

The fifth group is the most directly comparable to the present work, since its members also delegate the final decision to a classical learner operating on convolutional features: 3D-CNN-RF reached 95.00% and CNN-SVM 95.16%. The proposed configuration exceeds these by 1.58 and 1.42 percentage points, respectively, and does so notwithstanding that 3D-CNN-RF has access to volumetric context unavailable to the present two-dimensional formulation. Because the decision-layer strategy is shared across this group, the margin is attributable to the feature representation on which the decision layer operates rather than the pairing of a convolutional extractor with a classical head, which is the same conclusion reached independently by the ablation study in Section 4.8.

Across all five groups, the proposed CNN-KNN configuration recorded the highest reported accuracy in Table 14; however, these values were obtained under different datasets and experimental protocols and therefore do not constitute a controlled ranking of the methods. Under the hold-out protocol, its margin over the reviewed methods ranges from 0.98 to 16.41 percentage points, and when the comparison is restricted to the two entries evaluated by five-fold cross-validation, its 96.10% exceeds the 93.20% of EWHO-SqueezeNet by 2.90 points. It achieves this with a substantially smaller and more transparent architecture than the graph-based and large pretrained alternatives, which is consistent with the ablation findings reported above. The internal ablation results indicate that the performance of the proposed framework is associated with the combined contribution of MICA-based contrast normalization, the SE-augmented depth-wise separable backbone, and the distance-based decision layer, although the cross-study comparison in Table 14 cannot independently establish the contribution of these components relative to previously published methods.

The architectural contributions that result in better performance can be described as follows. First, dilated convolution provides the ability to detect multi-scale spatial features using an increased receptive field without raising the computational costs. Thus, this architectural contribution forms the basis for detecting tumor patterns of various sizes in medical images. Second, residual connections help solve the problem of vanishing gradients, thus allowing the training of a deeper network (through four encoding layers). Third, SE allows channel importance refinement and thus helps distinguish different types of tumors owing to less important channel suppression. Table 14 is consistent with the view that it is the composition of these elements with a distance-based decision layer, rather than any one of them in isolation, that accounts for the position the proposed framework occupies in the table. However, the extent to which this consistency may be pressed depends on the terms under which the tabulated figures were obtained, which are examined next.

The accuracies attributed to the competing methods were reproduced as reported by their original authors and were not obtained using a common experimental protocol. The referenced studies differ in the corpora on which they were evaluated, the number and definition of the diagnostic classes, the preprocessing and augmentation applied prior to training, and the resampling scheme used to estimate performance. Several studies reported a single split rather than a cross-validated mean. Differences on the order of one percentage point, such as the 0.98-point margin separating the proposed configuration from SE-DenseNet-121, are of the same magnitude as the variation attributable to these protocol differences alone and are comparable in width to the confidence intervals reported in Section 4.6. Therefore, Table 14 should be read as situating the proposed framework within the range of results currently reported for this task and not as evidence of a controlled superiority over any individual method.

The comparisons that were genuinely controlled in this study were internal. In Tables 12, 13, the dataset, preprocessing, partitioning, training schedule, and evaluation metrics are identical across every entry, and only the component under examination is varied, whereas the paired significance tests in Section 4.6 quantify the resulting differences on a per-sample basis. The claims advanced in this paper concerning the contribution of MICA enhancement, the individual architectural components, and the decision layer rest upon these controlled comparisons, whereas Table 14 serves the narrower purpose of demonstrating that the framework is competitive with the published state-of-the-art. A fully standardized benchmark, in which the leading competing architectures are re-implemented and retrained on the present corpus under a shared protocol, would be required to convert the comparison in Table 14 into a controlled one, which was identified in Section 4.14 as a direction for future work.

With respect to practical clinical applicability, the framework is positioned as a screening and second-reader aid, rather than as an autonomous diagnostic instrument. Its computational profile (Section 4.5) permits deployment on commodity hardware without a dedicated accelerator, and the class-conditional attention maps in Section 4.9 allow a reporting radiologist to inspect the evidence underlying each prediction. The constraints upon that positioning are equally explicit: the framework operates on individual two-dimensional slices and cannot substitute for volumetric assessment; meningioma recall remains the limiting quantity at 92.40% under the best configuration, which is insufficient for use as a rule-out test in isolation; and, as set out in Section 4.11, no external multi-institutional cohort has yet been used to verify that the reported performance transfers across scanners, acquisition protocols, and patient populations.

### 4.11 Robustness considerations

This subsection examines the extent to which the reported performance is contingent upon the properties of the evaluation corpus, considering the class imbalance, heterogeneity of image acquisition, sensitivity to noise, and the prospect of domain shift, and explicitly states which of these have been evaluated in this study and which have not.

The dataset used for the analysis of the proposed model was only mildly imbalanced. The four categories comprised 1,621 glioma, 1,645 meningioma, 1,757 pituitary, and 2,000 normal images, corresponding to 23.08, 23.42, 25.02, and 28.48% of the total, respectively; the ratio of the largest class to the smallest was 1.23, and the normalized Shannon entropy of the class distribution was 0.997, where unity denotes a perfectly uniform distribution. Three measures were adopted to ensure that this residual asymmetry did not inflate the reported figures. Both partitioning protocols are class-stratified, so that the hold-out set contains 324, 329, 351, and 400 images of the four classes in the same proportions as the parent corpus, and every cross-validation fold reproduces those proportions. All headline metrics were macro-averaged, weighting each class equally regardless of its size, and per-class recall, specificity, precision, and F1-score are reported individually in Tables 8, 9 rather than in aggregate. The empirical evidence that imbalance does not drive the results is that the ordering of class difficulty does not follow the ordering of class size: meningioma, the second-smallest category, is consistently the hardest, whereas glioma, the smallest, is recognized more accurately than meningioma under every decision layer and in both validation regimes. Therefore, the limiting factor is radiological similarity rather than the number of available examples.

Regarding variability in acquisition, the corpus is not the product of a single scanner or protocol; it aggregates three independently assembled collections, namely Figshare, SARTAJ, and Br35H (Section 3.1), and consequently spans a range of field strengths, pulse sequences, in-plane resolutions, and reconstruction pipelines. This heterogeneity is one of the reasons why the MICA front-end was formulated over multiple scales in the first instance. The coarse scale (*σ* = 60), with a cutoff frequency of approximately 0.0027 cycles per pixel, is intended to attenuate low-frequency intensity variations associated with coil sensitivity and B1-field inhomogeneity (Section 3.2.1). Finer scales preserve lesion-scale structure. The observation in Section 4.8 that MICA enhancement contributes 3.06 accuracy points and that no single scale reproduces this contribution is consistent with an interpretation in which part of the benefit of the enhancement stage may arise from reducing low-frequency intensity variation in addition to improving local contrast. This interpretation remains indirect, however, because the present study did not separately quantify the contribution of scanner or acquisition-specific intensity variation.

Sensitivity to noise has only been indirectly addressed. The amplification step in Equation 3 increases the magnitude of the high-frequency residual and therefore may also amplify acquisition noise alongside anatomical detail. The fidelity analysis in Section 3.2.1 provides a quantitative assessment of this trade-off: the MSE, PSNR, and SSIM values in Tables 3, 4 show a marked deterioration at *α* = 2.0, whereas *α* = 1.5 provides the best fidelity scores among the tested amplification factors and was therefore selected for the proposed configuration. However, these results do not constitute a direct noise-robustness evaluation, because the images were not subjected to controlled noise perturbations. A dedicated degradation experiment in which the hold-out images are perturbed with additive Gaussian or Rician noise, simulated bias fields, or motion artifacts at graded severity levels, followed by measurement of the resulting change in classification performance, has not been performed. Such an experiment would provide a direct assessment of the robustness of the complete pipeline and is therefore identified as a limitation and future research direction in Sections 4.14 and 5.

The same qualification applies with greater force to the domain shift. All results reported here were obtained from partitions of a single aggregated corpus, and although the three constituent collections introduced a measure of acquisition diversity, they did not constitute an external validation set; no images originating from an institution, scanner, or patient population unrepresented in training were used for testing. Because the publicly available aggregated corpus does not provide reliable patient-level identifiers for all constituent collections, the present partitioning was performed at the image level rather than at the patient level. Consequently, the possibility of correlated slices originating from the same subject cannot be completely excluded, and this limitation should be considered when interpreting the reported generalization performance. The close agreement between the hold-out and cross-validated estimates documented in Section 4.7 establishes that the reported performance is not an artifact of one favorable split. Still, it cannot establish that the performance transfers to a distributionally distinct cohort, which is a stronger property that only external validation can demonstrate. Accordingly, the generalization claims advanced in this study are confined to data drawn from the same distribution as the training corpus, and cross-institutional evaluation is identified in Sections 4.14 and 5 as the principal prerequisite for clinical translation.

### 4.12 Discussion

This subsection steps back from the individual experiments to interpret the patterns that recur across them, considering what the results imply from technical, clinical, and deployment perspectives. Several patterns that recur across the results merit interpretation beyond the numerical values themselves.

From a technical perspective, the consistent gap between CNN-KNN/CNN-SVM and the softmax head of the CNN model, together with the statistically significant McNemar’s tests, indicates that the decision rule provides an additional source of performance beyond the learned feature representation. This interpretation is consistent with the ablation study, which shows that dilation, residual connections, and MICA contribute larger performance gains than SE attention, indicating that spatial receptive-field expansion, stable gradient propagation, and contrast normalization are the principal factors shaping the learned representation, while channel attention and the classifier head provide smaller refinements. The difference between the accuracy and ranking results further indicates that the preferred classifier head depends on the intended use: CNN-KNN is preferable when the primary requirement is a categorical prediction, whereas CNN-SVM is more suitable when prediction scores are used for case ranking or selection of an operating threshold.

In the clinical sense, meningiomas continue to stand out among the classes, being the least distinguishable class with all classifiers and in any of the validation settings used, which is consistent with their radiological diversity. Unlike pituitary adenomas, meningiomas are usually less specific in appearance and can appear as faintly enhanced broad-based dural tumors with an intensity and morphology similar to that of the surrounding parenchyma and vasculature. This interpretation is corroborated by the analysis of the interpretability of the features above, which indicates that the location of the lesion remains relevant to the network, even in a difficult class.

From a deployment perspective, CNN-SVM is preferable when inference cost should remain largely independent of subsequent growth of the stored training set, because its support-vector set is fixed after training, whereas CNN-KNN requires distance calculations against the stored training embeddings and therefore scales with the number of stored training samples. In contrast, CNN-RF offers no statistically distinguishable benefit over the softmax head of CNN and therefore does not provide a clear justification for the additional decision-layer complexity for this embedding. Taken together, the statistically confirmed but comparatively modest gain of the best hybrid classifier over the base CNN, set against the larger contributions of the architectural components isolated in the ablation study, indicates that the principal source of performance is the MICA-enhanced, SE-augmented convolutional feature extractor, with the classifier head providing a smaller but statistically meaningful refinement.

### 4.13 Summary of results

This subsection consolidates the findings distributed across Sections 4.3 to 4.12 into a single account, tracing what was established, by which line of evidence, and with what degree of confidence.

Regarding comparative performance, the four decision layers were evaluated under two independent resampling regimes and returned the same ordering under both. CNN-KNN achieved the highest hard-label accuracy of 96.58% on the hold-out partition and 96.10 ± 0.83% across the five cross-validation folds, followed by CNN-SVM at 96.23% and 95.67 ± 0.57%. CNN-RF and the backbone’s native softmax head were statistically inseparable in both regimes, differing by less than one-tenth of the fold-level standard deviation under cross-validation. McNemar’s test confirmed that the accuracy gains of CNN-KNN and CNN-SVM over the softmax head were significant at *p* < 0.001, while the CNN-RF difference was not (*p* = 0.461), and paired bootstrap resampling placed the CNN-KNN improvement at 2.92 pp. with a 95% confidence interval of [1.71, 4.13] that excluded the zero.

Regarding the metric that should govern the choice of the decision layer, the evaluation exposed a consistent dissociation between hard-label accuracy and threshold-independent ranking quality. CNN-SVM attained the strongest ranking behavior in both regimes, with a macro-AUC of 99.56% on the hold-out set and 99.59 ± 0.11% across folds, together with the highest average precision, whereas CNN-KNN led to accuracy while recording the lowest AUC among the four configurations. The cause is architectural rather than incidental: the discrete neighbor-vote score of a KNN head cannot order cases finely across thresholds, whereas the continuous margin of an SVM can. The operational conclusion is that a distance-based head suits tasks that consume a categorical label, and a margin-based head suits tasks that consume a score.

The ablation study provides evidence regarding the contribution of the individual components to the framework’s performance. Disabling dilation cost 3.27 accuracy points, the bare backbone without squeeze-and-excitation or residual connections cost 3.20 points, removing the residual connection cost 3.13 points, and removing MICA contrast enhancement entirely cost 3.06 points, whereas restricting MICA to any single Gaussian scale cost 1.85–2.21 points and removing the SE block cost 0.85 points. These reductions are larger than the 2.15-point cross-validated gain attributable to the best decision layer, indicating that the main performance contribution arises from the MICA-enhanced, SE-augmented convolutional feature extractor, while the classifier head provides a smaller but statistically meaningful refinement.

Regarding clinical behavior, meningioma emerged as the limiting class in every configuration, every fold, and both validation protocols, with recall never exceeding 92% under cross-validation. Bidirectional glioma–meningioma confusion accounted for 43 to 50% of all residual errors, and the invariance of this pattern across four structurally dissimilar decision rules indicates that the limitation resides in the radiological signal available from a single two-dimensional slice rather than in the classifier. Recast as a binary screening question, the framework missed between 0.58 and 1.04% of tumor-bearing studies, depending on the head, with CNN-SVM performing best on both the missed-tumor and over-called-normal axes. SmoothGrad-CAM++ visualization confirmed that the predictions were anchored to anatomically plausible regions, including the difficult meningioma class.

Regarding efficiency and standing relative to prior work, the shared backbone requires only 107,076 parameters and 0.20 MB of half-precision weight storage while sustaining 23.7 images per second on the CPU and 220.1 images per second on an NVIDIA A100 GPU at a batch size of 16, making it substantially smaller than the pretrained backbones commonly applied to this task. The best configuration exceeded every reviewed state-of-the-art method listed in Table 14 by 0.98 to 16.41 pp. of accuracy under the hold-out protocol. Taken together, these results support a compact, contrast-normalized, attention-augmented feature extractor paired with a decision layer chosen to match the downstream clinical task as an accurate, interpretable, and computationally frugal basis for computer-aided brain tumor screening, subject to the constraints listed in Section 4.14.

However, that margin over the reviewed state-of-the-art methods is reported as an indication of competitiveness rather than as a controlled measure of superiority, since the studies compiled in Table 14 differ from one another in terms of dataset, preprocessing, and validation protocol; the qualification is described in detail in Section 4.10. The internally controlled evidence, comprising the ablation of Section 4.8, protocol comparison of Section 4.7, and paired significance tests and confidence intervals of Section 4.6, is unaffected by this heterogeneity.

### 4.14 Limitations

This subsection sets out the constraints that bound the interpretation of the results reported above and identifies the direction in which each constraint would need to be addressed.

The implemented framework shows multiple benefits, which we found to be restricted by its existing limitations.

1. Our first finding showed that the proposed system cannot currently explain its functions beyond the qualitative Grad-CAM analysis presented above, which limits its use in clinical settings without further quantitative interpretability validation or improvement. This will be addressed in future work through explainability methods, including Shapley additive explanations (SHAP), local interpretable model-agnostic explanations (LIME), and quantitative saliency-localization metrics.
2. The suggested framework was analyzed on 2D MRI slices and did not consider the 3D volumetric context of brain tumors, which can be critical for precise localization and staging.
3. The current model was evaluated using a publicly available single-source-aggregated dataset, which may not fully represent the real-world diversity of MRI across institutions, scanners, and patient demographics. The absence of external multi-institutional validation prevents a definitive assessment of the generalization capability of this framework.
4. The inference cost and memory footprint scale of the KNN decision layer with the size of the stored training set, which may become a practical constraint if the framework is retrained on substantially larger datasets in the future.
5. The ablation study retrained each variant for a reduced number of epochs relative to the full model to keep the computation tractable, which may modestly understate the achievable accuracy of some variants; publication-quality ablation numbers would require training every variant for the full epoch budget used for the main model.
6. The robustness of the pipeline under controlled degradation has not yet been quantified. No experiment was conducted in which the hold-out images were perturbed by additive Gaussian or Rician noise, simulated bias fields, or motion artifacts at graded severities. Therefore, the resistance of the framework to such corruptions remains uncharacterized, as discussed in Section 4.11 of this paper. A graded stress test of this kind, together with an assessment of calibration under corruption, is a necessary precondition for deployment in settings in which acquisition quality cannot be guaranteed.
7. The comparison with the published state-of-the-art presented in Table 14 reproduces the accuracies obtained under heterogeneous datasets, preprocessing pipelines, and validation protocols and is therefore indicative rather than controlled. Re-implementing the leading competing architectures and retraining them on the present corpus under a shared protocol would be necessary to place the comparison on a strictly equivalent footing.

## 5 Conclusion and future scope

This study presented a contrast-enhanced, deep-learning-based framework for automated multiclass brain tumor identification that couples a Multi-scale Intensity Contrast Amplification (MICA) preprocessing stage with a compact, squeeze-and-excitation-augmented, depth-wise separable convolutional backbone, and systematically compared four classifier heads—the CNN’s native softmax layer, KNN, SVM, and RF—trained on the resulting feature embedding. Under an 80:20 hold-out evaluation on 7,023 MRI images spanning glioma, meningioma, pituitary tumor, and normal classes, the CNN-KNN configuration achieved the highest accuracy (96.58%), whereas CNN-SVM achieved the best ranking-based performance (macro-AUC = 99.56%, macro-AP = 98.81%). Both improvements over the base CNN’s own softmax head were confirmed to be statistically significant by McNemar’s test and paired bootstrap resampling, and stratified 5-fold cross-validation applied to all four configurations confirmed that the accuracy advantage was not an artifact of a single favorable data split: the fold-averaged accuracies of 96.10 ± 0.83% for CNN-KNN and 95.67 ± 0.57% for CNN-SVM reproduced the hold-out ranking without exception, and the hold-out estimate fell within the observed fold range for every configuration. Gradient-based visualization further indicated that the network’s predictions were focused on anatomically plausible regions rather than extraneous background structures in the representative cases examined. The computational complexity analysis showed that the 107,076-parameter backbone requires 0.20 MB of half-precision storage and supports measured inference throughputs of 23.7 images per second on the CPU and 220.1 images per second on the GPU. These measurements indicate that the framework is computationally lightweight and could support efficient screening workflows and large-scale retrospective analysis, although prospective clinical deployment would require validation under real-world operating conditions. Taken together, these results support the conclusion that the principal source of diagnostic performance in this framework is the MICA-enhanced, SE-augmented convolutional feature extractor, with the pairing of that extractor to a distance-based (KNN) or margin-based (SVM) decision layer providing a smaller, but statistically confirmed, additional refinement, while a tree-ensemble (RF) decision layer offers no consistent benefit over the network’s own softmax classifier for this task.

Future work will proceed in several directions, motivated directly by the limitations noted above. First, explainability will be extended beyond the qualitative Grad-CAM analysis presented here to include quantitative-saliency-based localization metrics and complementary feature-attribution methods, such as SHAP and LIME, to build the case-level interpretability required for clinical adoption. Second, the stratified cross-validation protocol reported here will be extended to external multi-institutional MRI cohorts to assess generalization across scanners, acquisition protocols, and patient demographics beyond the present single-source Kaggle-aggregated dataset. Third, given that meningioma remains the most persistently confused class across every classifier and validation regime examined here, targeted strategies, including boundary-case oversampling, location or dural-contact priors, and volumetric (3D) context beyond the current 2D-slice formulation, will be explored specifically for this class rather than relying on further generic architectural changes. Fourth, the framework will be extended toward edge-device deployment, informed by the CPU latency and memory profile reported earlier, and toward integration with real-time segmentation and longitudinal tumor-progression tracking, to move from single-slice classification to a more complete computer-aided diagnostic pipeline.

The four directions set out above extend this method. Two further directions, which follow from the qualifications recorded in Sections 4.10 and 4.11 and are consequently the more immediate priority, are concerned with strengthening the evidence on which any claim about the method must rest. The first is a graded robustness evaluation, in which the test images are corrupted by additive Gaussian and Rician noise, simulated bias fields, and motion artifacts at controlled severities, so that the degradation of accuracy and predictive calibration may be characterized quantitatively rather than inferred from the image-fidelity measures of Section 3.2.1 alone. The second is a standardized benchmark in which the leading competing architectures are re-implemented and retrained on a common corpus under a shared preprocessing and validation protocol, which would convert the indicative comparison of Table 14 into a controlled one and permit the accompanying differences to be tested for their statistical significance. Until both have been carried out, the contribution of this work is properly stated as a compact, contrast-normalized, and interpretable classification framework whose internal design choices have been validated under controlled conditions and whose standing relative to the published literature has been established indicatively rather than definitively.

## Data Availability

The datasets presented in this study can be found in online repositories. The names of the repository/repositories and accession number(s) can be found at: https://www.kaggle.com/datasets/masoudnickparvar/brain-tumor-MRI-dataset.
